# Flexible statistical methods for estimating and testing effects in genomic studies with multiple conditions

**DOI:** 10.1101/2023.04.14.536893

**Published:** 2024-02-10

**Authors:** Yuxin Zou, Peter Carbonetto, Dongyue Xie, Gao Wang, Matthew Stephens

**Affiliations:** 1 Department of Statistics, University of Chicago, Chicago, IL, USA.; 2 Regeneron Genetics Center, Regeneron Pharmaceuticals, Inc., Tarrytown, NY, USA.; 3 Department of Human Genetics, University of Chicago, Chicago, IL, USA.; 4 Gertrude. H. Sergievsky Center, Department of Neurology, Columbia University, New York, NY, USA.

## Abstract

We introduce mvSuSiE, a multi-trait fine-mapping method for identifying putative causal variants from genetic association data (individual-level or summary data). mvSuSiE learns patterns of shared genetic effects from data, and exploits these patterns to improve power to identify causal SNPs. Comparisons on simulated data show that mvSuSiE is competitive in speed, power and precision with existing multi-trait methods, and uniformly improves on single-trait fine-mapping (SuSiE) in each trait separately. We applied mvSuSiE to jointly fine-map 16 blood cell traits using data from the UK Biobank. By jointly analyzing the traits and modeling heterogeneous effect sharing patterns, we discovered a much larger number of causal SNPs (>3,000) compared with single-trait fine-mapping, and with narrower credible sets. mvSuSiE also more comprehensively characterized the ways in which the genetic variants affect one or more blood cell traits; 68% of causal SNPs showed significant effects in more than one blood cell type.

## Introduction

Genome-wide association analyses (GWAS) have been performed for thousands of traits and have identified many genomic regions associated with diseases and complex traits [1, 2, 3, 4]. Many statistical fine-mapping methods have been developed to prioritize putative causal SNPs for a single trait [5, 6, 7, 8, 9, 10, 11, 12, 13, 14] [15, 16], but much fewer methods are available to fine-map multiple traits simultaneously. A simple strategy to fine-map multiple traits is to fine-map each trait separately then integrate the results *post hoc*. However, integration of results is not straightforward; for example, it is difficult to say whether signals identified in different single-trait analyses likely correspond to the same underlying causal SNP. Further, analyzing each trait independently is inefficient in that it cannot exploit the potential for increased power of a multivariate analysis [17]. Therefore, it is desirable to fine-map the traits simultaneously—that is, to perform *multi-trait fine-mapping*.

Although several methods have been developed for multi-trait fine-mapping [18, 19, 20, 21, 22, 23, 24, 25, 26] (Table 1), these methods have important practical limitations. For example, several methods are computationally impractical for more than a small number of traits, and most methods make restrictive assumptions about how SNPs affect the traits, such as that the effects of causal SNPs are uncorrelated among traits (e.g., [18, 19]). These assumptions are easily violated in fine-mapping applications; for example, in the blood cell traits considered in this paper, some genetic effects are specific to subsets of the traits (e.g., red blood cell traits). There are also several methods developed for the problem of colocalization of two traits (e.g., [27, 28, 29, 30]), which has different analysis aims, but overlaps with multi-trait fine-mapping.

Here we introduce mvSuSiE, a fast and flexible method for multi-trait fine-mapping. The name “mvSuSiE” evokes its origins as an extension of the Sum of Single Effects (SuSiE) model [13] to the multivariate analysis setting. In particular, mvSuSiE combines the SuSiE model with ideas from [31] to learn, in a flexible way, the patterns of shared genetic effects among traits. In this way, mvSuSiE automatically adapts to the patterns of effect sharing in the particular traits being analyzed, making it widely applicable to fine-mapping any set of related traits. We also leverage ideas from [16] to allow the analysis of summary statistics generated from a genetic association study which are often more accessible than individual-level data [32, 33]. mvSuSiE is computationally practical for jointly fine-mapping many traits in “biobank scale” data. We demonstrate its effectiveness compared with existing methods in simulations and by fine-mapping 16 blood-cell traits in 248,980 UK Biobank samples.

## Results

### Methods overview.

Consider fine-mapping R traits in a region containing J SNPs (or other biallelic loci). For each individual i=1,…,N, let yir denote trait r measured individual i, and let xij denote the genotype of individual i at SNP j, encoded as the number of copies of the minor allele. We perform multi-trait fine-mapping using the following multivariate linear regression model:

(1) $$y_{ir}=\mu_{r}+\sum_{J}^{j=1}x_{ij}b_{jr}+e_{ir},$$

where μr reflects the mean of trait r,bjr is the effect of SNP j on trait r, and the eirs are normally-distributed error terms (which may be correlated among the R traits). Within this regression model, we frame fine-mapping as a “variable selection problem”: most SNPs are assumed to have no effect on any trait—that is, most effects bjr are zero—and the goal of multi-trait fine-mapping is to identify which SNPs have a non-zero effect on which traits, and to assess uncertainty in these inferences. (For brevity, we use the term “causal SNP” to mean a SNP with non-zero effect.)

Our mvSuSiE method achieves this goal by extending the *Sum of Single Effects* (SuSiE) model [13] to the multivariate setting. By using ideas from [16], it can perform fine-mapping using either individual-level data (genotypes and phenotypes) or summary data (e.g., LD matrix and marginal z-scores); see Online Methods for details.

Among existing approaches to fine-mapping, mvSuSiE is most closely related to CAFEH [23], which also extends SuSiE to perform multi-trait fine-mapping. Both CAFEH and mvSuSiE inherit much of the simplicity and benefits of single-trait SuSiE. Like SuSiE, both mvSuSiE and CAFEH require the user to specify an upper bound, L, on the number of causal SNPs in a region, and are robust to this upper bound being larger than needed. And both methods exploit SuSiE ‘s simple fitting procedure, Iterative Bayesian Stepwise Selection [13], which is similar to simple forward stepwise selection, but improves on it by (i) using Bayesian computations to take into account uncertainty in which SNPs are selected at each step; and (ii) iterating through selection events to allow errors in initial selections to be corrected as fitting progresses.

However, mvSuSiE improves on CAFEH in two key ways:

(a) mvSuSiE uses a flexible prior distribution—a mixture of multivariate normal distributions, as in [31]—to model effect sharing patterns across traits. Further, the parameters of this prior are estimated from the data, allowing mvSuSiE to adapt to each data set. This flexible approach allows that different causal SNPs may show different patterns of association; for example, in analyses of blood cell traits shown later, mvSuSiE learns that some SNPs may affect primarily red blood cell (erythrocyte) traits, some may affect primarily white blood cell (leukocyte) traits, and some may affect both, or a subset of one or the other. In contrast, CAFEH assumes a less flexible and less adaptive prior in which causal effects are independent across traits.

(b) mvSuSiE allows for correlations in measurements among traits, with these correlations again being estimated from the data. In contrast, CAFEH assumes measurements are independent across traits, which is often not the case because association studies often involve correlated traits.

For (a), estimating the prior distribution from the data involves combining information across many causal SNPs from many regions, which is an additional step compared with standard single-trait fine-mapping analyses. This additional step can be avoided by using a simpler fixed prior (see Online methods) but at potential loss of power.

We also introduce novel ways to summarize the inferences from multi-trait fine-mapping. Again, this builds on SuSiE, which summarizes single-trait results by reporting, for each SNP, a “posterior inclusion probability” (PIP) quantifying the probability that the SNP is causal, and by reporting “credible sets” (CSs) [7, 13] that are designed to capture, with high probability, at least one causal SNP. Informally, each CS represents an independent association signal in the data, and the size of a CS (*i.e.,* the number of SNPs in the CS) indicates how precisely one can pinpoint the causal SNP underlying this signal. For multi-trait analyses, it may seem natural to report PIPs and CSs separately for each trait. However, this raises tricky issues; for example, if the reported CSs for two traits overlap, do these represent the “same” signal, with a single underlying causal SNP, or different signals with multiple causal SNPs? To avoid this problem, we separate inference into two questions.

#### First question:

Which SNPs are causal for *at least one trait*? This question is answered by *cross-trait* PIPs and CSs that summarize the inferences across all traits.

#### Second question:

For each causal SNP (*i.e*., CS) identified, which traits does it affect? This is answered by computing a *trait-wise* measure of significance, the *local false sign rate* (*lfsr*) [31, 34], for each SNP in each trait (with small *lfsr* indicating high confidence in the *sign* of the effect). Because SNPs in a CS are typically in high LD, their trait-wise *lfsr* values are typically similar, and it is convenient to use a single number, the *average lfsr*, as a trait-wise measure of significance of each CS. If the average *lfsr* for trait r is small, this indicates high confidence in the sign of the effect, and we say the CS is “significant for trait r.”

**Figure 1 |:**
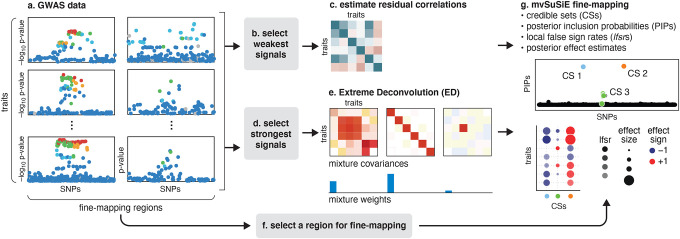
Overview of multivariate fine-mapping using mvSuSiE. mvSuSiE accepts as input traits and SNP genotypes measured in N individuals, R traits and M target fine-mapping regions. Alternatively, mvSuSiE-RSS accepts SNP-level summary statistics (a) computed from these data (see Online Methods, “mvSuSiE with summary data: mvSuSiE-RSS”). The weakest SNP association signals are extracted from these data (b), which are used in (c) to estimate correlations in the trait residuals (see Online Methods, “Estimating the residual variance matrix”). Separately, the strongest association signals are extracted (d) to estimate effect sharing patterns (e) using Extreme Deconvolution (ED) [35] (see Online Methods, “Specifying the prior”). Finally, the effect-sharing patterns estimated by ED, together with the estimated weights, are used to construct a prior for the unknown multivariate effects, and this prior is used in mvSuSiE to perform multivariate fine-mapping simultaneously for all SNPs in a selected region (g). Steps f and g are repeated for each fine-mapping region of interest. The key mvSuSiE outputs are: a list of credible sets (CSs), each of which is intended to capture a distinct causal SNP; a posterior inclusion probability (PIP) for each SNP giving the probability that the SNP is causal for at least one trait; average local false sign rates (*lfsr*s) summarizing significance of each CS in each trait; and posterior estimates of SNP effects on each trait. For example, if a region contains 3 distinct causal SNPs, mvSuSiE will, ideally, output 3 CSs, each containing a true causal SNP, with the average *lfsr*s indicating which traits are significant for each CS. These quantities are defined in the Online Methods.

In summary, the reported results from a mvSuSiE analysis are the *cross-trait* PIPs and CSs together with *trait-wise* measures of significance (*lfsr*) for each SNP and each CS in each trait. Fig. 1 summarizes the mvSuSiE analysis workflow for a typical genetic association study.

### Evaluation in simulations using UK Biobank genotypes.

We compared mvSuSiE with existing multi-trait fine-mapping methods and a single-trait fine-mapping method, SuSiE [13, 16], in simulations. Among available multi-trait fine-mapping methods (Table 1), MT-HESS [18] and BayesSUR [21, 36, 37] are similar to mvSuSiE in features and modeling assumptions, but are computationally impractical for large fine-mapping data sets. msCAVIAR [22] shares the ability of mvSuSiE to model effect sharing, but is designed for analyzing data from multiple studies, and therefore makes modeling assumptions that are less appropriate for analyzing multiple traits. MFM [24] is another multi-trait fine-mapping method, but is specific to multiple case-control traits with a shared set of controls. Therefore, we focussed our comparisons on CAFEH [23] which can handle large multi-trait fine-mapping data sets. We also compared with flashfm [20] and PAINTOR [19] on smaller fine-mapping data sets with two traits.

To make our simulations reflective of current large-scale genomic data sets, we obtained imputed genotype data from the UK Biobank [38] and simulated quantitative traits with 1–5 simulated causal SNPs in each fine-mapping region. We simulated from a variety of effect sharing patterns, with effect sizes scaled to roughly reproduce the distributions of z-scores observed in genome-wide association analyses of complex traits from UK Biobank data. The fine-mapping regions were drawn from autosomal chromosomes and varied in size (0.4–1.6 Mb), number of SNPs (1,000–5,000 SNPs) and LD patterns.

We simulated traits under two scenarios:

(a) “Trait-specific + Shared Effects,” in which the SNP effects on 20 independent traits were either specific to one trait, or shared among traits in simple ways (e.g., equal effects on a pair of traits and no effect on the remaining traits);

(b) “Complex Shared Effects,” in which the SNP effects on 16 correlated traits were generated from a variety of sharing patterns derived from the UK Biobank blood cell traits.

To compare with PAINTOR and flashfm, we also simulated a third smaller set of data with 2 independent traits and shared effects.

We compared methods in their detection of cross-trait causal SNPs—in which we define a cross-trait causal SNP as one that affects at least one trait—and trait-wise causal SNPs. We assessed the performance of both SNP-wise measures (e.g., PIPs) and Credible Sets (CSs) for these tasks. Since PIPs and CSs are typically used in combination in a fine-mapping analysis—CSs identify the independent causal signals then PIPs identify the strongest causal SNP candidates within each CS—this experiment evaluates the two main aspects of a typical fine-mapping analysis.

**Figure 2 |:**
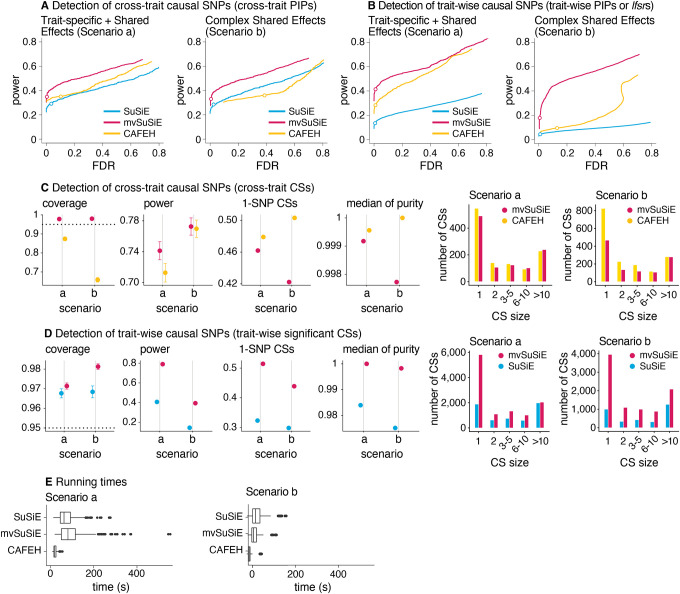
Comparison of fine-mapping methods in simulated data. Panels A and B show power vs. FDR in identifying causal SNPs, either cross-trait (A) or trait-wise (B), using SNP-wise measures. In each scenario, FDR and power were calculated by varying the measure threshold is varied from 0 to 1 (n=600 simulations). Open circles are drawn at a PIP threshold of 0.95 or *lfsr* threshold of 0.05. The specific SNP-wise measures used in A are PIP (mvSuSiE, CAFEH), max-PIP (SuSiE); in B, PIP (SuSiE), *min-lfsr* (mvSuSiE) and “study PIP” (CAFEH). FDR = FP/(TP + FP) and power = TP/(TP + FN), where FP, TP, FN, TN denote, respectively, the number of false positives, true positives, false negatives and true negatives. Panels C and D evaluate the estimated CSs using the following metrics: *coverage*, the proportion of CSs containing a true causal SNP; *power*, the proportion of true causal SNPs included in at least one CS; the proportion of CSs that contain a single SNP (“1-SNP CSs”); and *median purity*, where “purity” is defined as the smallest absolute correlation (Pearson’s *r*) among all SNP pairs in a CS. Histograms of CS sizes (number of SNPs in a 95% CS) are given for each scenario. Target coverage (95%) is shown as a dotted horizontal line. In D, mvSuSiE detected a trait-wise effect if the average *lfsr* in the CS was less than 0.05. Error bars show 2 times the empirical s.e. from the results in all simulations. Panel E summarizes runtimes; the SuSiE runtimes are for running SuSiE independently on all traits. The box plot whiskers depict 1.5 times the interquartile range, the box bounds represent the upper and lower quartiles (25th and 75th percentiles), the center line represents the median (50th percentile), and points represent outliers.

In all our comparisons, mvSuSiE improved power, coverage and resolution (purity and proportion of 1-SNP CSs) over the SuSiE single-trait analyses (Fig. 2A, B, D; n = 600 simulations). The greatest gains were in Scenario b, where mvSuSiE had the advantage that it accounted for correlations among traits. Comparing CAFEH and single-trait SuSiE in SNP-wise inferences (Fig. 2A, B), CAFEH improved performance in Scenario a, but performed slightly less well for detecting causal SNPs in Scenario b, where it produced poorly calibrated PIPs (Fig. 2A, B, Supplementary Fig. 9). Comparing CSs (Fig. 2C), CAFEH improved the purity of the CSs and the proportion of 1-SNP CSs, but these improvements were tempered by CAFEH’s reduced power and coverage, particularly in Scenario b. A partial explanation for these results is that Scenario b contradicts CAFEH’s assumptions of independent traits and independent causal effects. In support of this explanation, when we forced mvSuSiE to make the same independence assumptions as CAFEH, mvSuSiE’s performance was reduced and the PIPs were also poorly calibrated (see the “random effects prior” and “independent traits” results in Supplementary Figures 1, 2, 9). These results illustrate the benefits of having a flexible model that can adapt to different fine-mapping scenarios by learning effect-sharing patterns from the data (Supplementary Figures 1, 10–13). This flexibility comes at a computational cost—CAFEH was consistently faster than mvSuSiE (Fig. 2E, Supplementary Table 1)—but mvSuSiE was fast enough to handle the largest fine-mapping data sets we considered.

We also compared mvSuSiE with CAFEH, PAINTOR and flashfm in a variety of simpler fine-mapping data sets simulated in a similar way to above but with only two traits (Supplementary Figures 3–8). Even when the traits were simulated independently in accordance with PAINTOR’s modeling assumptions, PAINTOR had much lower power to detect causal SNPs than both SuSiE and mvSuSiE (Supplementary Fig. 3A). Both flashfm and mvSuSiE improved power over the SuSiE single-trait analyses, but mvSuSiE achieved much greater gains in power (Supplementary Figures 3, 5–8). mvSuSiE also had considerably lower computational cost than PAINTOR and flashfm (Supplementary Fig. 4, Supplementary Table 1). The performance of CAFEH in these simpler simulations was similar to mvSuSiE except when the two traits were highly correlated (Supplementary Fig. 5).

In summary, these simulations demonstrate the benefits of mvSuSiE as an efficient and flexible multi-trait fine-mapping method. In particular, mvSuSiE consistently increased power to detect causal SNPs, improved precision (reduced CS size) compared with fine-mapping each trait separately, and is the only method that provides both *cross-trait* and *trait-wise* significance measures.

### Multi-trait fine-mapping of blood cell traits from UK Biobank.

To illustrate mvSuSiE in a substantive application, we fine-mapped blood cell traits using data from the UK Biobank [38]. Previous analyses of these data include association analyses [39, 40] and single-trait fine-mapping [41, 42], but multi-trait fine-mapping using mvSuSiE has the potential to improve power and precision of fine-mapping. Multi-trait fine-mapping is also better for answering questions about shared genetic effects—which SNPs affect which traits—and hence provide insights into the underlying biology.

Focusing on a subset of 16 blood cell traits (Supplementary Table 2), we performed standard PLINK association analyses [43] with n=248,980 UK Biobank samples for which all 16 traits and imputed genotypes were available (Online Methods). We included covariates such as sex and age, as well as genotype principal components to limit spurious associations due to population structure. From the results of these association analyses, we obtained 975 candidate genomic regions for fine-mapping (Supplementary Table 3). We then applied the mvSuSiE analysis pipeline to these 975 candidate regions (Online Methods). To understand the benefits of a multi-trait fine-mapping, we also ran SuSiE on the same regions, separately for each trait.

### Genetic relationships among blood traits inform discovery of multi-trait causal SNPs.

From the 975 candidate regions, mvSuSiE identified 3,396 independent causal signals (95% cross-trait CSs). The median size of a CS was 7 SNPs. Among these CSs, 726 contained just one SNP (“1-SNP CS”); therefore, mvSuSiE identified 726 high-confidence candidate causal SNPs (PIP > 95%; Supplementary Table 4). Several of these 1-SNP CSs (36) were not identified in *any* of our single-trait (SuSiE) analyses, underscoring the benefits of combining evidence across genetically related traits. Reassuringly, 496 of the 726 SNPs were also identified as high-confidence causal SNPs (PIP > 95%) in the single-trait analyses of [42] (and 145 of 726 overlapped with [41]).

**Figure 3 |:**
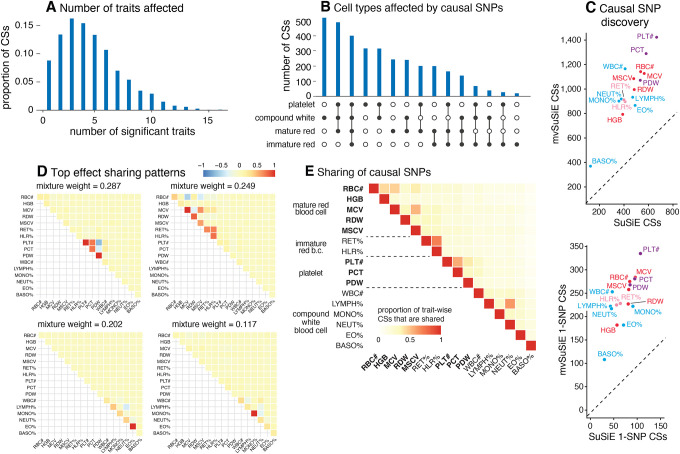
mvSuSiE fine-mapping and primary effect sharing patterns in UK Biobank blood cell traits. Panels A, B and E give summaries of the 3,396 mvSuSiE CSs identified from the 975 candidate fine-mapping regions: (A) number of significant (*lfsr* < 0.01) traits in each CS; (B) significant traits in CSs grouped by blood cell-type subsets; (E) pairwise sharing of significant CSs among the traits. In E, for each pair of traits we show the ratio of the number of CSs that are significant in both traits to the number of CSs that are significant in at least one trait. (C) Number of CSs and 1-SNP CSs for each trait identified by SuSiE and mvSuSiE (after removing CSs with purity less than 0.5). In C, each mvSuSiE count is the number of mvSuSiE CSs or 1-SNP CSs that are significant (*lfsr* < 0.01) for the given trait. (D) Covariance matrices in the mvSuSiE data-driven prior capturing the top sharing patterns (these are the covariance matrices with the largest mixture weights in the prior). The covariance matrices were scaled separately for each plot so that the plotted values lie between –1 and 1. See Supplementary Fig. 11 for the full set of 15 sharing patterns.

The number of CSs significant in each trait (*lfsr* < 0.01) ranged from 370 (basophil percentage) to 1,423 (platelet count), and the number of 1-SNP CSs ranged from 108 to 335 (Fig. 3C). (Note that 10 of the 3,396 CSs were not significant in any traits at *lfsr <* 0.01.) Notably, mvSuSiE increased fine-mapping discovery and resolution compared to SuSiE single-trait fine-mapping: the number of trait-wise significant CSs increased, on average, 2.2-fold compared with SuSiE, and the number of trait-wise significant 1-SNP CSs increased, on average, 3.5-fold (Fig. 3C).

The fine-mapped SNPs from mvSuSiE were generally slightly more enriched for genomic regulatory annotations than those for SuSiE (Supplementary Fig. 15), providing indirect support for the additional mvSuSiE findings being driven by real signals rather than false positives. For example, the mvSuSiE-fine-mapped SNPs had an enrichment odds ratio of 11.9 for being an eQTL compared to 9.7 from SuSiE. We also analyzed enrichment of the fine-mapped SNPs for accessible chromatin regions in hematopoietic cell-types [41] (Supplementary Figures 16, 17 and Supplementary Tables 6, 7). Similar to [42], both the SuSiE and mvSuSiE results showed some of the expected enrichments such as enrichment of SNPs affecting platelet-related traits for open chromatin in platelet-producing megakaryocytes.

mvSuSiE improved discovery and resolution over single-trait analysis by learning and exploiting patterns of shared (and not shared) genetic effects from the data. In these data, the most prominent learned patterns involved strong sharing of effects amongst traits for the same blood cell type (Fig. 3D). However, many other patterns were also identified (Supplementary Fig. 11), including both trait-specific and broad effects, suggesting that SNPs can affect blood cells in a wide variety of ways, presumably reflecting a wide variety of underlying biological mechanisms. By applying mvSuSiE with a prior that incorporates these learned sharing patterns, we obtain a genome-wide summary that underscores the diversity of genetic effects on blood cell traits (Fig. 3A, B, D). Genetic effects are more commonly shared among traits within the same blood cell type as one might expect (Fig. 3E), but SNPs affecting multiple blood cell types are also common (Fig. 3B).

### Multi-trait fine-mapping reveals highly heterogeneous genetic determination of blood traits.

**Figure 4 |:**
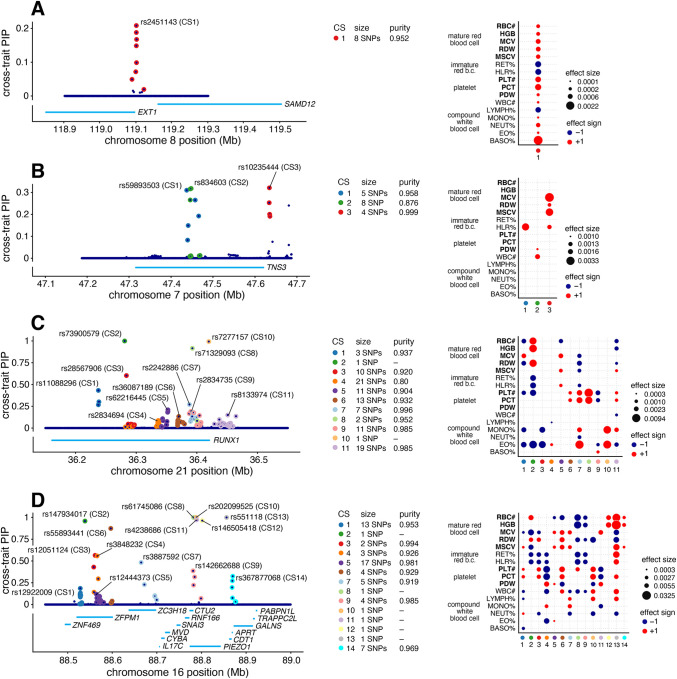
Examples of blood cell trait loci fine-mapped using mvSuSiE. The left-hand plots are “PIP plots” showing the cross-trait posterior inclusion probabilities (PIPs) for each SNP analyzed in the given fine-mapping region. The cross-trait PIP is an estimate of the probability that the SNP is causal for at least one trait. The labeled SNPs are the “sentinel SNPs”, the SNPs with the highest cross-trait PIP in each CS. “Purity” is defined as the minimum absolute pairwise correlation (Pearson’s r) among SNPs in the CS. The right-hand plots show the posterior effect estimates of the sentinel SNPs whenever the CS is significant for the given trait (*lfsr* < 0.01). All estimates and tests are from a data sample of size n=248,980.

To illustrate the potential for mvSuSiE to help dissect complex genetic association signals, we examine four example regions in more detail (Fig. 4).

Fig. 4A shows the mvSuSiE results for the *EXT1–SAMD12* locus. Single-trait association analysis of this region shows only one trait, basophil percentage, with a genome-wide significant association (PLINK two-sided t-test p-value < 5 × 10^−8^). Similarly, single-trait fine-mapping with SuSiE identified a single CS for basophil percentage containing 10 candidate SNPs and no CSs in other traits. From the SuSiE results one might conclude that the causal SNP is specific to basophil percentage. However, the mvSuSiE fine-mapping results assess the CS as significant in most traits, suggesting that in fact the causal SNP has broad effects across many traits. (Indeed, all traits had marginal association p- values less than 0.003 with the lead SNP, which in other settings might be considered “significant”.) The mvSuSiE CS is smaller than the single-trait CS (8 vs. 10 SNPs), illustrating the improved fine-mapping resolution that can come from combining information across traits.

Fig. 4B shows mvSuSiE results for the Tensin 3 (*TNS3*) locus. Vuckovic et al [42] used single-trait fine-mapping to identify causal signals for several red and white blood cell traits in this region. However, a single-trait analysis does not tell us whether these signals are due to one or a few causal SNPs affecting many blood cell traits, or many causal SNPs affecting individual traits. The multi-trait mvSuSiE analysis identified three causal signals (cross-trait CSs) with three distinct patterns of genetic effect: one mostly affects red blood cell traits (CS3); another has a detectable effect in HLR% only (CS1); and a third has smaller effects in both white blood cell and platelet traits (CS2). The three very different patterns suggest that the biological effects of these SNPs are also very different, and suggest a multi-faceted role for *TNS3* in affecting blood-cell traits. This example illustrates the flexibility of mvSuSiE, including its ability to capture different patterns of effect-sharing even within a single locus, and its ability to extract relatively simple inferences in quite complex situations.

Fig. 4C shows a more complex example involving many signals in and around the gene *RUNX1*. SNPs in the *RUNX1* locus have previously been associated with rheumatoid arthritis [44, 45] and other immune-related diseases (e.g., [46, 47]), and colocalization analyses have suggested that the causal SNPs are also associated with eosinophil proportions in blood [42]. Multi-trait fine-mapping results from mvSuSiE suggest a complex picture with 11 signals (cross-trait CSs), each with detectable effects in many different blood-cell traits, and some with no detectable effect on eosinophil proportions. These results suggest that the mechanisms by which this gene affects immune-related diseases may be more complex than just through eosinophils, possibly involving many platelet, red blood cell and other white blood cell traits.

Finally, Fig. 4D shows a more complex example still, where many causal signals are mapped to a region containing many genes, including *PIEZO1* and *ZFPM1*. This is a gene-dense region with well-studied connections to blood cell traits and blood-related diseases [48, 49, 50, 51, 52]. mvSuSiE identified 14 independent signals (cross-trait CSs) in the region. These 14 signals show a wide variety of effect patterns; for example, some are significant only in a few traits related to mature red blood cells (e.g., CS12, CS14), some are significant across a broader range of red blood cell traits (CS2), and some are significant across most traits (CS13). Regions of this complexity may take considerable additional investigation to fully understand. Although this is a complex example, we note that of the 14 CSs identified in this region, 7 contain a single SNP, demonstrating that even in complex regions mvSuSiE can identify high-confidence causal SNPs.

## Discussion

We have introduced mvSuSiE, a fast and flexible multi-trait fine-mapping method. mvSuSiE outperformed single-trait fine-mapping methods in both power and resolution. Unlike most available multi-trait fine-mapping methods, mvSuSiE can efficiently analyze dozens of correlated traits and can model complex patterns of effect size variation via a flexible data-driven prior distribution. The prior model also includes as special cases several simpler models that are commonly used in meta-analyses, such as the fixed effects model which assumes equal effects in all traits and the random effects model which allows for different effect sizes among traits [53]. These models can be used in place of the data-driven prior to speed computation if users desire, though at a potential loss of power.

The mvSuSiE model is “sparse” in that it assumes a small number of causal SNPs. However, the data-driven prior model for the effect sizes at these causal SNPs will not generally be sparse. That is, each causal SNP is effectively assumed to affect all traits, with some exceptions (such as when the prior includes sharing patterns reflecting an effect in one trait and no effects in the others). Instead of inducing trait-specific sparsity on the effects of causal SNPs, we focussed on *estimating* these effects and assessing their significance by computing the *lfsr*s. This approach simplifies computation and worked well in our examples. Indeed, in additional simulation studies we found that mixture models constructed using our data-driven approach could capture the predominant sparsity patterns reasonably well (Supplementary Fig. 12), and so mvSuSiE did not suffer from a loss of power to detect such sparse association signals. That being said, it is possible that explicitly modeling trait-specific sparsity of causal SNPs could be helpful in settings with large numbers of traits that are less related; with a large number of less related traits, the SNP effects may be shared primarily among small subsets of more related traits. This could perhaps be achieved by combining the mvSuSiE prior with indicator variables for each trait, similar to the strategy used by CAFEH.

mvSuSiE assumes a standard (Gaussian) multivariate linear model (1) and so is most applicable to quantitative traits. However, provided the effect sizes are small, there is good theoretical and empirical justification for applying standard linear models directly to binary traits [54, 55]. Thus, in genetic association studies where individual SNP effects are small, it would be reasonable to apply mvSuSiE directly to studies where some or all of the traits are binary (e.g., disease status). When fine-mapping with summary data, this would mean applying mvSuSiE-RSS to the summary statistics from a *linear regression analysis* of the binary traits. While it may seem more intuitive to generate the summary statistics from a *logistic regression analysis* of the binary traits, there seems to be less theoretical or empirical support for this approach, even in the univariate case. More generally, there has been little theoretical or empirical assessment of univariate fine-mapping methods using summary data from linear mixed models or from generalized linear models, or for that matter any model other than a simple linear regression model; more work in this area seems important.

The multivariate linear model (1) assumes that the traits are measured in the same samples, with no missing data. When the traits involved are not independent—or, more precisely, when the residual error terms are not independent—correctly dealing with missing data in this model is complicated. Indeed, even maximum-likelihood estimation under a simple “missing-at-random” assumption becomes quite involved [56]. In cases with small amounts of missing data, we suggest imputing the missing values before fine-mapping. (This suggestion applies to both a full-data analysis and a summary-data analysis. Analyses of summary data computed with large or unknown amounts missingness should proceed with caution, if at all.) This “pre-imputation” approach may lose power compared with more rigorous approaches, but is more straightforward. If there are larger amounts of missing data, then it may be necessary to focus on a subset of samples with more overlap in available measurements. This may result in a tradeoff between sample size and number of traits analyzed; for example, with a large amount of missing data, it may be more powerful to analyze a subset of the traits at a larger sample size.

Some multivariate association analyses involve traits measured in non-overlapping sets of individuals. Examples include colocalization of expression QTLs and GWAS traits in different samples [28]; multi-ancestry [22] and meta-analysis fine-mapping [32]; and fine-mapping of multiple non-overlapping diseases with a common control set. Formally, these could be treated as versions of (1) with extensive missing trait data. However, in practice these special cases yield important simplifications of the problem, and therefore it may be preferable to treat these special cases separately, with dedicated software implementations. Indeed, other groups have already successfully developed versions of SuSiE for some of these settings [57, 58, 59].

## Online Methods

### Multivariate multiple regression.

mvSuSiE is based on a basic multivariate multiple regression model for R quantitative traits observed in N individuals,

(2) $$Y\sim MN_{N\times R}\left(XB,I_{N},V\right),$$

where $Y\in R^{N\times R}$ is a matrix storing N observations of R traits, $X\in R^{N\times J}$ is a matrix of N genotypes at J SNPs, $B\in R^{J\times R}$ is a matrix of regression coefficients (“effects”) for the J SNPs and R traits, V is an R×R covariance matrix (we assume V is invertible), $I_{N}$ is the N×N identity matrix, and ${MN}_{N\times R}\;(M,\Sigma^{\text{row}},\Sigma^{\text{col}}$) denotes the matrix normal distribution [1, 2] with mean $M\in R^{N\times R}$ and covariance matrices $\Sigma^{\text{row}},\Sigma^{\text{col}}$ (of dimension N×N and R×R, respectively).

### Intercept.

We do not explicitly include an intercept in (2). Instead, we account for an intercept implicitly by “centering” the columns of X and the columns of Y so that the mean of each column is zero. From a Bayesian perspective, centering the columns of X and Y is equivalent to integrating with respect to an (improper) uniform prior on the intercept. (This is a multivariate generalization of the result for univariate regression given in [3]. See the Supplementary Note for a more formal proof of this result.) In short, centering eliminates the need to explicitly include an intercept in (2), and we proceed with mvSuSiE assuming that X and Y have been centered.

### The mvSuSiE model.

mvSuSiE generalizes the “Sum of Single Effects” (SuSiE) model [4] to the multivariate setting:

(3) $$B=\sum_{l=1}^{L}B^{\left(l\right)}$$

(4) $$B^{\left(l\right)}=\gamma^{\left(l\right)}⊗b^{\left(l\right)},$$

where $\gamma^{(l)\;}\in \;{\left\{0,\;1\right\}}^{J}$ is a vector of indicator variables in which exactly one of the J elements is one and the remaining are zero, $b^{\left(l\right)}\in R^{R}$ is a vector of regression coefficients, and $u\;⊗\;v=uv^{⊤}$ denotes the outer product of (column) vectors u and v. The coefficients B defined in this way are a sum of L “single effects” $B^{\left(l\right)}$ In particular, matrix $B^{\left(l\right)}\in R^{J\times R}$ has at most one row containing non-zero values, and these non-zero values are determined by $b^{\left(l\right)}$. We therefore refer to $B^{\left(l\right)}\;$ as a “single effect matrix” because it encodes the effects of a single SNP. The final set of coefficients, B, is a matrix with at most L rows containing non-zero values.

Similar to SuSiE, we introduce priors for the indicator variables $\gamma^{\left(l\right)}$ and regression coefficients $b^{\left(l\right)}$,

(5) $$\gamma^{\left(l\right)}\sim \text{Multinom}\left(1,\pi \right)$$

(6) $$b^{\left(l\right)}\sim g_{l},$$

in which Multinom(1, π) denotes the multinomial distribution for m random multinomial trials with category probabilities $\pi =(\pi_{1},\;\ldots ,\;\pi_{J})$, such that $\pi_{j}\geq 0,\sum_{j=1}^{J}\pi_{j}=1$. The πj's are the prior inclusion probabilities. By default, we assume a uniform prior; that is, $\pi_{j}=1/J$, for j=1, …, J. (All the results in this paper use this default prior.) Our software implementation of mvSuSiE also support for other choices of π; for example, π could be determined by external biological information about the SNPs (e.g., [5]).

The prior distribution $g_{l}$ for each single effect $b^{\left(l\right)}$ should capture the variety of effect sharing patterns we expect from the multiple traits. To this end, we use a prior similar to the mixture of multivariate normals prior introduced in [6],

(7) $$g_{l}\left(b\right)=\sum_{k=1}^{K}\omega_{k}N_{R}\left(b;0,\sigma_{0l}^{2}U_{k}\right),$$

in which each $U_{k}$ is a (possibly singular) covariance matrix, $\sigma_{0l}^{2}\geq 0$ scales the prior for each single effect l, $\omega =(\omega_{1},\;\ldots ,\;\omega_{K})$ is a vector of mixture weights, such that $\omega_{k}\;\geq \;0,\sum_{k=1}^{K}\omega_{k}=1$, and $N_{d}(x;\;\mu ,\;\Sigma )$ denotes the multivariate normal distribution on $x\in R^{d}$ with mean $\mu \in R^{d}$ and d×d covariance Σ. The covariance matrices $𝒰=\{U_{1},\;\ldots ,\;U_{K}\}$ and the mixture weights ω must be chosen beforehand, whereas prior scaling parameters $\sigma_{01}^{2},\ldots ,\sigma_{0L}^{2}$ are treated as unknown, and are estimated from the data.

In summary, mvSuSiE is a multivariate regression model with a flexible mixture-of-normals prior on the “single effects,” $b^{\left(l\right)}$. The unknowns of primary interest are the single effect matrices $B^{\left(l\right)}$. As we explain in more detail below, we compute a posterior distribution of the single effects, which is then used to compute key fine-mapping statistics, specifically the posterior inclusion probabilities (PIPs) and credible sets (CSs). The scaling factors $\sigma_{0l}^{2}$ are not of primary interest to the fine-mapping (“nuisance parameters”), and are estimated from the data to aid in better posterior estimation of the single effects. Other model parameters, such as the residual covariance matrix V, are assumed to be known, or should have been estimated previously. Below we give guidance on choosing these parameters or estimating them from data.

### Posterior computation approach.

Here we outline our approach to estimating the posterior distribution for the unknowns of primary interest, the single effect matrices $B^{\left(1\right)},\;\ldots ,\;B^{\left(L\right)}$, building on the ideas introduced in [7]. A more formal mathematical development of the mvSuSiE algorithms is given in the Supplementary Note.

In this section, we assume that the model parameters V, π, ω and 𝒰, as well as L, the maximum number of single effects, are known, or have been estimated in earlier steps in the analysis. We also assume in this section that the scaling factors $\sigma_{01}^{2},\ldots ,\sigma_{0L}^{2}$ are known (in the Supplementary Note we describe how the scaling factors are estimated).

The posterior distribution of $B^{\left(1\right)},\;\ldots ,\;B^{\left(L\right)}$, as in other Bayesian variable selection models, is intractable, and therefore we must resort to numerical approximations. A key consideration is that we would like these computations to scale well to large genetic data sets, which makes intensive Monte Carlo techniques such as Markov chain Monte Carlo (e.g., [8, 9, 10, 11, 12, 13, 14, 15] less attractive. Another key consideration is that we would like accurate estimates of posterior quantities which can be difficult to achieve when many variables (the SNPs) are highly correlated, or correlated in complicated ways, which is typically the case in genetic fine-mapping. These considerations, as well as others, motivated us to develop an alternative posterior computation approach for SuSiE based on variational approximation ideas [4]. The algorithm for performing the approximate posterior computations in SuSiE was called “Iterative Bayesian Stepwise Selection” (IBSS). In this paper, we have extended the SuSiE variational approach to the mvSuSiE model. Therefore, applying the ideas developed in [4] leads to an IBSS algorithm for fitting the mvSuSiE model (Algorithm 1 in the Supplementary Note).

### Choice of L.

The number of effects, L, is typically not known in advance. However, mvSuSiE is generally robust to misspecification of L so long as L is chosen to be larger than the (true) number of effects. That’s because mvSuSiE prunes single effects when they are not needed by estimating the scaling factors $\sigma_{0l}^{2}$ in the prior (7) as zero or close to zero. This approach to estimating the number of single effects, L, by adapting the prior is closely related to “automatic relevance determination” [16, 17], and this same approach was used in SuSiE [4].

### Extension of posterior computation approach to work with summary data.

The strategy used in [7] to extend SuSiE to summary data is quite general, and we take this same here: first, in “mvSuSiE with sufficient statistics,” we describe an algorithm that uses *sufficient statistics*, and produces the same result as running the mvSuSiE IBSS algorithm on the individual-level data; in “mvSuSiE with summary data: mvSuSiE-RSS”, we consider summary data that approximate the sufficient statistics, and therefore yield results that do not exactly reproduce mvSuSiE with individual-level data; and since many genetic association studies provide z*-*scores, or other summary statistics that can be used to compute z-scores, we describe mvSuSiE-RSS with z-scores in greater detail (“Special case when X, Y are standardized: mvSuSiE-RSS with z-scores”).

### mvSuSiE with sufficient statistics.

The data enter the mvSuSiE model only through the likelihood, which from (2) is

(8) $$l\left(B;X,Y\right)=|2\pi V{|}^{-\frac{N}{2}}\text{exp}\left\{-\frac{1}{2}\text{tr}\left[V^{-1}\left(Y^{⊤}Y-2B^{⊤}X^{⊤}Y+B^{⊤}X^{⊤}XB\right)\right]\right\}.$$

Here we treat V as a fixed quantity so we do not explicitly mention this dependency in the notation for the likelihood. It is clear from this expression that the data influence the likelihood only through the quantities $X^{⊤}Y$ and $X^{⊤}X$. Therefore, $X^{⊤}Y$ and $X^{⊤}X$ are *sufficient statistics* for B. Thus, by rearranging the computations, we obtain a variant of the IBSS algorithm that fits the mvSuSiE model using only sufficient statistics. We call this algorithm “IBSS-ss”, and it is outlined in Algorithm 2 in the Supplementary Note.

We use IBSS(X, Y) to denote the result of applying the IBSS algorithm (Algorithm 1 in Supplementary Note) to the individual-level data, and we use $\text{IBSS-ss}(X^{⊤}X,\;X^{⊤}Y)$ to denote the result of applying the IBSS-ss algorithm (Algorithm 2 in Supplementary Note) to the sufficient statistics. These two algorithms will give the same result, $\text{IBSS}(X,\;Y)=\text{IBSS-ss}(X^{⊤}X,\;X^{⊤}Y)$. However, the computational complexity of the two approaches is different. The computational complexity of one iteration of the IBSS algorithm is $O\;(L\times (NJR+{KJR}^{3}))$, whereas the complexity of a single iteration of the IBSS-ss algorithm is $O\;(L\times (J^{2}R+{KJR}^{3}))$. Therefore, when N ≫ J, which is often the case in fine-mapping studies, IBSS-ss will usually be faster. We note, however, that computing the J×J matrix $X^{⊤}X$ can be expensive, and potentially more expensive than running mvSuSiE itself. So IBSS-ss will be more computationally attractive than IBSS if N is much larger than J and if $X^{⊤}X$ can be computed efficiently using a software such as PLINK [18] or LDStore [19].

### mvSuSiE with summary data: mvSuSiE-RSS.

We define mvSuSiE-RSS as the application the IBSS-ss algorithm to the sufficient statistics or approximations to these statistics (e.g., an LD estimate obtained from different genotype data than the genotype data used to obtain the other statistics). Conceptually, this approach combines the mixture prior (7) with an approximation to the likelihood (8). To formalize this, we write the likelihood as a function of the sufficient statistics,

(9) $$l_{SS}\left(B;S_{xx},S_{xy},N\right)=|2\pi V{|}^{-\frac{N}{2}}\text{exp}\left(-\frac{N}{2}\text{tr}\left[V^{-1}\left(\frac{Y^{⊤}Y}{N}-2B^{⊤}S_{xy}+B^{⊤}S_{xx}B\right)\right]\right),$$

so that

(10) $$l_{SS}\left(B;\frac{X^{⊤}X}{N},\frac{X^{⊤}Y}{N},N\right)=l\left(B;X,Y\right).$$

Replacing $S_{xx}=\frac{X^{⊤}X}{N}$ with an estimate ${\hat{S}}_{xx}\approx S_{xx}$ is therefore the same as replacing the sufficient-statistics likelihood (9) with

(11) $$l_{RSS}\left(B\right)=l_{SS}\left(B;{\hat{S}}_{xx},\frac{X^{⊤}Y}{N},N\right).$$

Note that when ${\hat{S}}_{xx}=S_{xx}$, the approximation is exact; that is, $l_{RSS}(B)=l(B;\;X,\;Y)$.

In summary, applying mvSuSiE with $S_{xx}$ is equivalent to using the individual-data likelihood (8), and applying mvSuSiE with $S_{xx}$ is equivalent to using the approximate likelihood (11).

### Special case when X, Y are standardized: mvSuSiE-RSS with z-scores.

Now we consider the special case when X and Y are standardized, which is common in genetic association studies. By “standardized”, we mean that the columns of X and Y have been scaled to have unit variance; $\sum_{i=1}^{N}x_{ij}^{2}=N,j=1,\ldots ,J$, and $\sum_{i=1}^{N}y_{ir}^{2}=N,r=1,\ldots ,R$. (See [7] for exact definitions of the z-scores and the LD matrix R.) This is in addition to the assumption, mentioned earlier, that the columns of X and Y are centered to have means of zero. See [20, 21] for a discussion on the choice to standardize.

With standardized X and Y, the sufficient statistics $X^{⊤}Y$ and $X^{⊤}X$ can be recovered from the sample size, N, the (in-sample) LD matrix, R, and the marginal association z-scores, ${\overset{ˆ}{z}}_{jr}$, which are obtained from simple linear regressions between the traits r and the SNPs j. (The z-scores should ideally be computed using the same samples for each trait so that the correlations among SNPs are same for all traits.) In particular, the sufficient statistics are recovered by the following two equations,

(12) $$X^{⊤}X=N\times R$$

(13) $$X^{⊤}Y=\sqrt{N}\times \tilde{Z},$$

in which $\tilde{\text{Z}}$ denotes the J×R matrix of “adjusted z-scores”,

(14) $${\tilde{z}}_{jr}=\frac{N}{N+{\hat{z}}_{jr}}\times {\hat{z}}_{jr}.$$

Note that, when the effects are small, ${\tilde{z}}_{jr}\approx {\overset{ˆ}{z}}_{jr}$.

Substituting equations (12–13) into the sufficient-statistics likelihood (9) gives

(15) $$l_{SS}\left(B;S_{xx},S_{xy},N\right)=l_{SS}\left(B;R,\frac{\tilde{z}}{\sqrt{N}},N\right).$$

When the in-sample LD matrix R is not available, and is replaced with $\hat{R}\approx R$, the mvSuSiE-RSS likelihood (11) becomes

(16) $$l_{RSS}\left(B\right)=l_{SS}\left(B;\hat{R},\frac{\tilde{z}}{\sqrt{N}},N\right).$$

In summary, when X and Y are standardized, applying mvSuSiE with R is equivalent to using the individual-data likelihood (8), and applying mvSuSiE with $\hat{R}$ is equivalent to using the approximate likelihood (16).

### mvSuSiE posterior statistics.

Here we describe the posterior statistics used in an mvSuSiE fine-mapping analysis.

### Basic posterior quantities.

We start with two basic posterior statistics that are used to calculate other statistics. The first posterior quantity is the posterior probability that the lth single effect is nonzero for SNP j,

(17) $$\alpha_{j}^{\left(l\right)}≔\text{Pr}\left(\gamma_{j}^{\left(l\right)}=1|X,Y\right).$$

The second posterior quantity, denoted $\text{clfs}r_{jr}^{\left(l\right)}$, is the *local false sign rate* [6, 22] for SNP j in trait r and single effect l *conditioned on* SNP j having a nonzero effect in single effect l,

(18) $$\text{clfs}r_{jr}^{\left(l\right)}≔1-\text{max}\left\{\text{Pr}\left(b_{jr}^{\left(l\right)}>0|X,Y,\gamma_{j}^{\left(l\right)}=1\right),\text{Pr}\left(b_{jr}^{\left(l\right)}<0|X,Y,\gamma_{j}^{\left(l\right)}=1\right)\right\}.$$

Intuitively, the *clfsr* (“conditional lfsr”) measures how confident we can be in the sign of the effect of SNP j in trait r and single effect l given that SNP j has a nonzero effect in single effect l. A small *lfsr* indicates high confidence in the sign of an effect. The *lfsr* is robust to modeling assumptions [22] which is helpful for reducing sensitivity to the choice of prior.

### Cross-trait PIP.

The posterior inclusion probability (PIP) is a standard quantity reported by most fine-mapping methods, so PIPs are convenient for comparing performance of different fine-mapping methods. PIPs are also useful for visualizing the fine-mapping signal within a candidate fine-mapping region. For mvSuSiE, we define the PIP for SNP j as the posterior probability that at least one of the regression coefficients for the jth SNP is not zero,

(19) $${\text{PIP}}_{j}≔\text{Pr}\left(b_{j}\neq 0|X,Y\right)\;=1-\text{Pr}\left(b_{j}=0|X,Y\right)\;=1-\prod_{l=1}^{L}\left(1-\alpha_{j}^{\left(l\right)}\right),$$

in which $\alpha_{j}^{\left(l\right)}$ is defined in (17).

### min-lfsr.

The PIP tells us whether or not a SNP has an effect on at least one trait, but it does not tell us *which traits* are affected by the SNP. To quantify this, we calculate a *minimum lfsr* (*min-lfsr*), which we define as the smallest lfsr among the L single effects,

(20) $$lfsr_{jr}≔\underset{l\in \left\{1,\ldots ,L\right\}}{\text{min}}lfsr_{jr}^{\left(l\right)},$$

in which $lfsr_{jr}^{\left(l\right)}$ is the (unconditional) lfsr for SNP j in outcome r and single effect l,

(21) $$\text{lf }sr_{jr}^{\left(l\right)}≔1-\text{max}\left\{\text{Pr}\left(b_{jr}^{\left(l\right)}>0|X,Y\right),\text{Pr}\left(b_{jr}^{\left(l\right)}<0|X,Y\right)\right\}$$

(22) $$=1-\alpha_{j}^{\left(l\right)}\left(1-\text{clfs}r_{jr}^{\left(l\right)}\right),$$

and we use the definition of $\text{clfs}r_{jr}^{\left(l\right)}$ from (18). In other words, SNP j is considered “significant” in trait r if and only if it is significant in at least one of the L effects.

### Credible sets.

A cross-trait credible set $\text{CS}(\alpha^{\left(l\right)};\;\rho )$ is defined as a set of SNPs that has probability at least ρ of containing an effect SNP [23]. The calculation of cross-trait CSs is described in [4].

A CS does not indicate *which traits* are affected by the SNPs. To assess significance of a CS for a specific trait r, we compute the *average lfsr*, defined as a weighted average of the conditional *lfsr*’s for all SNPs,

(23) $$lfsr_{r}^{\left(l\right)}≔\sum_{l=1}^{L}\alpha_{j}^{\left(l\right)}\text{clfs}r_{jr}^{\left(l\right)}.$$

If the *average lfsr* for trait r is small, this indicates high confidence in the sign of the effect, and so we say the effects of the SNPs in the CS are *significant for trait r* (“trait-wise CS”).

### Specifying V and g.

In order to run mvSuSiE or mvSuSiE-RSS, we must first specify the R×R residual variance-covariance matrix, V, and the prior on the regression coefficients, g. In the next two sections, we describe the steps that were taken to specify these model parameters in the simulations and the UK Biobank blood traits case study. Since we always applied mvSuSiE to summary data (“mvSuSiE-RSS”), and specifically z-scores, we describe estimation of V and g using the z-scores.

### Estimating the residual variance matrix.

When the traits are measured in the same samples, it is important to account for possible correlations among the measurements of the different traits; failure to do so can result in miscalibrated fine-mapping statistics (Supplementary Figures 2 and 9). The residual covariance matrix V is used to account for correlations among the measurements; the special case of independent measurements can be modeled by setting V to a diagonal matrix.

To estimate V, we adapted the approach described in [6], in which V was estimated from z*-*scores (e.g., z-scores obtained from marginal association tests). Specifically, we took the following steps. First, we pooled the z-scores from all the fine-mapping regions considered. Then we filtered out large (in magnitude) z-scores; specifically, we only considered SNPs in which the largest z-score magnitude for any trait was less than 2. This improved the estimate of V by removing SNPs that might affect one or more of the traits. Denoting the number of SNPs used in this calculation by J′, and letting ${\hat{\text{z}}}_{\text{j}}$ denote the vector of z-scores obtained from the R association tests for SNP j, we estimated V as

(24) $$\hat{V}=\frac{1}{J\prime }\sum_{j=1}^{J\prime }{\hat{z}}_{j}{\hat{z}}_{j}^{⊤}.$$

To verify this estimator, in the simulations we compared mvSuSiE using the estimate (24) to mvSuSiE with the sample covariance of Y (Supplementary Fig. 2). Although genetic effects should also ideally be removed before estimating V, in the simulations the genetic effects were all very small, and so should have little impact on this estimate.

We estimated V for the UK Biobank blood cell trait data using $J^{\prime }=1,950$ SNPs (2 SNPs with small z-scores were selected from each of the 975 fine-mapping regions). This estimate of V is given in Supplementary Table 5 (n=1,950). Since the blood cell traits were standardized, we scaled the estimate so that the final V used in the fine-mapping analyses was a correlation matrix.

### Specifying the prior.

The prior (7) can accommodate many different effect sharing patterns. However, to maximize the benefit of using this prior, it should capture the effect sharing patterns that are actually present in the data. Following [6], we considered three approaches to obtaining *g* (in the simulations, we assessed the advantages of each of these approaches; see Supplementary Fig. 1):

- The simple “random effects prior” that assumes the effects are independent across traits. This is a special case of (7) in which the mixture consists of a single mixture component $\left(K=1,\;\omega_{1}=1\right)$ with covariance matrix $U_{1}=I_{R}$. Although simple, this prior is used—implicitly or explicitly—by methods that assume the effects are independent across traits.
- Prior with a mixture of “canonical” sharing patterns. (See “Canonical prior” below for details.) This prior is not as flexible as the “data-driven” prior described next, but has the advantage of being easy to implement because it does not involve any separate estimation steps.
- A “data-driven” prior in which the covariances and weights are estimated from the data. The basic idea behind this prior is to adapt the sharing patterns $U_{k}$ and corresponding mixture weights $\omega_{k}$ to be consistent with the data. (See “Data-driven prior” below for details.) This requires more work to design but has a potentially greater payoff.

### Canonical prior.

The generation of the canonical covariance matrices for the prior g is implemented by the create_cov_canonical function in the mvsusieR package. Following [6], this function generates the following covariance matrices: the R×R identity matrix, $I_{R}$, modeling the case when all effects are independent (the same as the “random effects” prior); the “equal effects” matrix, an R×R matrix of all ones, which models the case in which all effects are the same; rank-1 matrices modeling trait-specific effects of the form $e_{r}e_{r}^{⊤}$, in which $e_{r}$ is a vector of length R containing all zeros except for a 1 in the rth position; and matrices modeling uniformly heterogeneous effects, with ones on the diagonal and σ on the off-diagonal, where σ is 0.25, 0.5 or 0.75. In total, this resulted in R+5 covariance matrices, where R is the number of traits.

Note that the canonical covariance matrices are all at the same scale (each matrix has entries spanning the range 0 to 1), and none of the matrices allow for negatively correlated effects (all of the entries in these matrices are non-negative).

To complete the canonical prior, we assigned uniform weights $\omega_{k}=1/K$ to the K=R+5 mixture components.

### Data-driven prior.

We also took an approach similar to [6] to generate the covariance matrices $U_{k}$ and mixture weights $\omega_{k}$ in the “data-driven” prior.

First, we prepared a data set to learn the prior. For each candidate fine-mapping region, we identified the top z-score which was defined as the vector of association z-scores for the R traits containing the largest (in magnitude) z-score among all SNPs in the given fine-mapping region. Letting M denote the number of fine-mapping regions, we formed an M×R matrix containing the top z-scores. Here we denote this matrix by Z.

Next, we generated *initial estimates* of covariance matrices using a variety of approaches:

- R+5 canonical covariance matrices (see “Canonical prior” above).
- The empirical covariance matrix $Z^{⊤}Z/M$.
- Three rank-1 matrices of the form $v_{r}v_{r}^{⊤},r=1,2,3,r=1,\;2,\;3$, in which $v_{r}$ is the rth right singular vector of the reduced singular value decomposition (SVD) of $Z,Z=\sum_{r=1}^{R}\sigma_{r}w_{r}v_{r}^{⊤}$, in which $\sigma_{r}$ is the r-th singular value and $w_{r}$ is the rth left singular vector.
- A rank-3 approximation of Z based its SVD, $U\approx \sum_{r=1}^{3}\sigma_{r}^{2}v_{r}v_{r}^{⊤}/M$.
- A sparse, low-rank approximation of Z obtained using the R package flashr [24] (version 0.6–8), $U\;\approx \;{FL}^{⊤}{LF}^{⊤}/M$, where L is the M×R’ loadings matrix and F is the R×R' matrix of estimated factors, and R’ ≤ R is the rank of the approximation. The rank, R’, was automatically determined by flashr.
- R’ rank-1 matrices of the form $f_{r}f_{r}^{⊤},r=1,\ldots ,R^{\prime }$, in which $f_{r}$ denotes the rth column of F.

After completing these steps, we had initial estimates for K=R+R’+1 covariance matrices.

Next, we ran Extreme Deconvolution (ED) [25] to refine the initial estimates of the $U_{k}$ and simultaneously estimate the mixture weights $\omega_{k}$. (We used the ED algorithm implemented in the cov_ed function from the mashr R package, version 0.2.59, which was adapted from [25].) The mixture weights initialized to uniform values, $\omega_{k}=1/K,\;k=1,\;\ldots ,\;K$.

Finally, to avoid poor estimation of the lfsr that can happen when the prior covariances are singular (*i.e*., not invertible), we added a small positive constant to the diagonals of all the covariance matrices $U_{k}$ in the data-driven prior. This step ensured that these matrices were all invertible.

The data-driven prior obtained by running the above procedure on the UK Biobank data is shown in Supplementary Fig. 11. The data-driven priors obtained by running the above procedure separately in Scenarios a and b of the simulations are shown in Supplementary Figures 12 and 13, respectively.

### UK Biobank data.

The UK Biobank is a prospective cohort study with detailed phenotype and genotype data collected from approximately 500,000 participants recruited in the United Kingdom, with ages between 40 and 69 at time of recruitment [26, 27]. For fine-mapping, we focused on a subset of 16 blood cell traits from the UK Biobank haematology data collection [28]. These blood cell traits were also the focus of a recent association analysis [29, 30] and fine-mapping studies [31, 32]. Several of the UK Biobank blood cell traits are based on the same measured quantities and are therefore highly correlated so we did not include all the blood cell traits in our analyses. For example, relative volume of erythrocytes, also known as “hematocrit” (HCT), is calculated from mean corpuscular volume (MCV) and red blood cell count (RBC#), so to avoid including highly correlated traits we did not include HCT. The blood cell traits used in our fine-mapping analyses are summarized in Supplementary Table 2.

The UK Biobank imputed genotypes feature a high density of available SNPs, so they are well-suited for fine-mapping [26, 27]. We used a subset of the 502,492 available UK Biobank genotypes (version 3), removing samples that met one or more of the following criteria for exclusion: mismatch between self-reported and genetic sex; pregnant; one or more data entries needed for the analysis or data preparation steps are missing; and, following [29, 32], a blood-related disease was reported in the hospital in-patient data (blood-related diseases included were leukemia, lymphoma, bone marrow transplant, chemotherapy, myelodysplastic syndrome, anemia, HIV, end-stage kidney disease, dialysis, cirrhosis, multiple myeloma, lymphocytic leukemia, myeloid leukemia, polycythaemia vera, haemochromatosis). Additionally, we excluded outlying genotype samples based on heterozygosity and/or rate of missing genotypes as defined by UK Biobank (data field 22027), and we removed any individuals having at least one relative in the cohort based on UK Biobank kinship calculations (samples with a value other than zero in data field 22021). Finally, to limit confounding due to population structure, we included only genotype samples marked as “White British” (based on a principal components analysis of the genotypes [26] stored in data field 22009). After filtering genotype samples according to these criteria, 257,605 samples remained.

We applied quantile normalization to the 16 blood cell traits measured in the 257,605 samples, separately for each trait, to transform each trait to the standard normal distribution. Since ultimately we aimed to jointly model the 16 blood cell traits, we removed outlying phenotypes according to a simple multivariate normal distribution of the phenotypes. Specifically, after quantile normalization, we measured the Mahalanobis distance $y_{i}^{⊤}{\hat{\Sigma }}^{-1}y_{i}$ for each individual i, where $y_{i}$ is the vector of 16 blood cell traits measured in individual i, and $\hat{\Sigma }$ is the sample covariance matrix estimated from the 257,605 UK Biobank samples. We discarded samples with Mahalanobis distance falling within the [0.99, 1] quantile of the chi-square distribution with 16 degrees of freedom. This step removed 8,625 samples, for a final total of 248,980 UK Biobank samples.

Base-pair positions of the SNPs in the UK Biobank genotype data are reported using Genome Reference Consortium human genome assembly 37 (hg19).

### Association analyses of UK Biobank blood cell traits.

Using the UK Biobank genotype and phenotype data prepared as described above, we computed association statistics for each of the 16 blood cell traits and for all available biallelic SNPs on autosomal chromosomes meeting the following criteria: minor allele frequency of 0.1% or greater; information (“INFO”) score of 0.6 or greater (the INFO score quantifies imputation quality). The same criteria were used in [33] to filter the SNPs.

Association statistics were computed using the --glm function in PLINK (version 2.00a2LM, 64-bit Intel, Feb 21, 2009) [18] with hide-covar no-x-sex omit-ref –vif 100. Following [34, 32], we included the following covariates in the association analyses: sex (data field 31), age at recruitment (21022), age × age, assessment center (54), and genotype measurement batch (22000). To limit inflation of spurious associations due to population structure, we also included the top 10 genotype PCs as covariates following previous association analyses of UK Biobank data (e.g., [35]). (These PCs were previously computed by UK Biobank [26] and stored in data field 22009.) The covariates input file for PLINK was prepared by calling the model.matrix function in R and standardizing quantitative covariates (age, PCs) to have mean 0 and variance 1.

The summary data provided as input to SuSiE and mvSuSiE were the z-scores and p-values extracted from the T_STAT and P columns in the plink2 --glm outputs. The association statistics computed using PLINK have been made available in a Zenodo repository (see URLs).

### Selection of regions for fine-mapping.

To select regions for fine-mapping, we adapted the approach used in [32] to the multivariate setting. In brief, we began by identifying regions separately for each trait. For each significant association (PLINK two-sided t-test p-value less than 5 × 10^−8^), we defined the fine-mapping region as all SNPs within ±250 kb of the significant association. Next, any regions overlapping by one or more SNPs were combined into a larger region. We repeated combining regions until no regions overlapped. This resulted in a set of fine-mapping regions for each of the 16 blood cell traits, similar to [32]. To form a single set of fine-mapping regions for all 16 traits, we then merged two regions from different traits whenever they overlapped. The end result of this procedure was a set 975 of disjoint fine-mapping regions satisfying these properties: all “significant SNPs” (with PLINK p-value for two-sided t-test less than 5 × 10^−8^) belong to exactly one region; all SNPs within 250 kb of a significant SNP belong to exactly one region. This procedure generated fine-mapping regions that varied considerably in size: their lengths ranged from 411 kb to 8.73 Mb (average size: 961 kb; median size: 686 kb); and the number of SNPs ranged from 93 SNPs to 36,605 SNPs (average number of SNPs: 4,776; median number of SNPs: 3,514). A listing of all 975 regions is given in Supplementary Table 3. These same regions were used in both single-trait and multi-trait fine-mapping.

Note that we did not fine-map the extended MHC [36], defined as base-pair positions 25−−36 Mb on chromosome 6. The MHC is particularly challenging to analyze and interpret, and therefore is typically analyzed separately [37, 38, 39].

### Simulations using UK Biobank genotypes.

We evaluated the fine-mapping methods on data sets generated using real genotypes X and simulated phenotypes Y. For the genotypes, we used the UK Biobank imputed genotypes. We simulated Y from different mvSuSiE models.

The genotype data were curated following the data preparation steps described above, so N=248,980 in all our simulations. To clarify, these data preparation steps included removing outlying blood cell trait observations (see above). Even though this particular filtering step was not needed since we did not use the UK Biobank phenotype data in the simulations, for convenience we used the data prepared with this filtering step.

### Simulation scenarios.

We implemented three fine-mapping scenarios in the simulations.

In the simplest simulations, which we used to compare all of the methods (SuSiE, mvSuSiE, CAFEH, PAINTOR and flashfm), we simulated 2 traits under a variety of conditions: (i) independent traits with independent effects; (ii) independent traits with correlated effects; and (iii) correlated traits with independent effects. This simpler scenario was intended mainly for comparisons with PAINTOR and flashfm so as to not unfairly disadvantage these methods: flashfm cannot handle a large number of traits; PAINTOR cannot handle a large number of causal SNPs, and assumes independent traits and independent effects (Table 1). However, for completeness we also compared with SuSiE and CAFEH in this simulation scenario.

For comparing other fine-mapping methods (SuSiE, mvSuSiE, CAFEH), we simulated data sets under two more complex scenarios, which we refer to as “Scenario a” and “Scenario b”.

In Scenario a, we simulated 20 independent traits in which the SNP effects were either specific to one trait or shared among traits in simple ways (equal effects among 2 traits, equal effects among half of the traits, or correlated equally among all 20 traits). In the results, we call Scenario a the “Trait-specific + Shared Effects” scenario.

Scenario b was intended to capture a combination of factors that one might more realistically encounter in fine-mapping studies. It is also more challenging because the traits are correlated and the effects are shared among the traits in complex ways. Specifically, we simulated using a residual covariance matrix V and sharing patterns $U_{k}$ obtained from our analyses of the UK Biobank blood cell traits. In the results, we refer to Scenario b as the “Complex Shared Effects” scenario.

### Simulation procedure.

Let X denote the N×J genotype matrix for a given fine-mapping region, where J is the number of SNPs in the region, and N=248,980.

The procedure we used to simulate an N×R matrix Y was the following.

1. Center and scale the columns of X so that each column has a mean of 0 and a variance of 1.
2. Choose S, the number of causal SNPs. For Scenarios a and b, set S to 1, 2, 3, 4 or 5 with probabilities 0.3, 0.3, 0.2, 0.1, 0.1, respectively. For the 2-trait simulations, set S=2.
3. Sample the indices of the S causal SNPs uniformly at random from {1, …, J}. Denote the set of causal SNPs by 𝒞.
4. For each SNP j ∈ 𝒞, simulate the R effects, $b_{j}\in R^{R}$, from the mixture of multivariate normals (7), in which $\sigma_{0l}^{2}=1$. In the 2-trait simulations, we set $K=1,\;\omega_{1}=1,U_{1}=\left[\begin{array}{ll} 1 & \rho \\ \rho & 1 \end{array}\right]$,, in which the correlation ρ between the two effects was 0, 0.5 or 1. We also simulated 2-trait data sets in which the effects were simulated from a mixture of R+5=7 “canonical” covariance matrices (see “Canonical prior” above). To draw each effect $b_{j}$ from this mixture, the mixture component probabilities were specified as follows: one of the two trait-specific covariances was chosen each with probability 0.2; or one of the remaining canonical covariances was chosen each with probability 0.12. In Scenario a, we simulated the effects of the causal SNPs $b_{j}$ using a mixture of 19 covariance matrices (Supplementary Fig. 10). For Scenario b, we simulated the effects using the mixture of 15 covariance matrices estimated from the UK Biobank data (Supplementary Fig. 11).
5. For each SNP j ∉ 𝒞, set $b_{j}=0$.
6. Choose the residual variance $\sigma^{2}$. To set $\sigma^{2}$ to a realistic value, we set $\sigma^{2}$ so that the greatest proportion of variance in a trait explained by the SNPs was 0.05%, which roughly corresponds to the proportion of variance explained in the mvSuSiE fine-mapping analyses of the UK Biobank blood cell traits. In particular, we solved for $\sigma^{2}$ satisfying $\frac{\sigma_{g}^{2}}{\sigma^{2}+\sigma_{g}^{2}}=0.0005$ where $\sigma_{g}^{2}≔\text{Var}\left({\hat{y}}_{r}\right)$ is the variance in the rth trait explained by the SNPs, in which Var(θ) denotes the sample variance, ${\hat{y}}_{r}≔x_{1}b_{1r}+\cdots +x_{J}b_{Jr}$, and $r≔{\text{argmax}}_{r^{\prime }\in \{1,\ldots ,R\}}\text{Var}\left({\hat{y}}_{r}\right)$.
7. Specify the R×R residual correlation matrix C, then set $V=\sigma^{2}C$. For Scenario a and the 2-trait scenario, $C=I_{R}$. For Scenario b, the 16 × 16 covariance matrix C was set to the correlation matrix estimated from the 16 blood cell traits after removing the linear effects of covariates (Supplementary Table 5). (Note that, although this correlation matrix was estimated from the UK Biobank data, for the simulations, the V used in the mvSuSiE analyses of the simulated data was estimated using the simulated data and so the V used by mvSuSiE differed from the V used to simulate Y.) In the 2-trait simulations, we set $C=\left[\begin{array}{cc} 1 & c_{12}\\ c_{21} & 1 \end{array}\right]$, in which the correlation $c_{12}$ between the two effects was 0, 0.4 or 0.8.
8. Simulate Y using model (1).
9. Center and scale the columns of Y so that each column has a mean of zero and a variance of 1.
10. Compute the summary statistics—effect estimates ${\overset{ˆ}{\beta }}_{jr}$, standard errors ${\overset{ˆ}{s}}_{jr}$, z-scores $z_{jr}$ and the in-sample LD matrix R—using PLINK [18] and LDstore [19]. For these summary statistics, we extracted the BETA, SE, T_STAT and P columns from the plink2 --glm output (see “Association analyses of UK Biobank blood cell traits” for more details on how PLINK was called). Note PLINK was applied to the raw genotypes without centering or scaling. We computed the J×J in-sample LD matrix $\hat{R}=D^{-1/2}X^{⊤}XD^{-1/2}$, where $D≔\text{diag}(X^{⊤}X)$, using LDstore version 1.1.

This procedure produced an empirical distribution of z-scores roughly similar to the z-scores seen in association analyses of the blood cell traits; in our simulations, the largest z-score magnitude in each fine-mapping region had a median of 11.05, mean of 10.97 and a third quantile of 11.71, whereas the corresponding statistics for the UK Biobank blood cell traits were 8.01, 10.85 and 12.18.

For each of the three scenarios (Scenario a, Scenario b, and the 2-trait scenario), we simulated 600 data sets for 600 fine-mapping regions selected from the curated set of 975 regions for the UK Biobank blood cell traits (Supplementary Table 3). All selected regions had at least 1,000 SNPs and no more than 5,000 SNPs, and were at least 400 kb in size and at most 1.6 Mb. In total, we simulated 600 × 3 = 1,800 fine-mapping data sets.

### Details of the methods compared.

In this section we describe how we ran the methods on the simulated data sets.

SuSiE, mvSuSiE and PAINTOR were run using the z-scores and in-sample LD. CAFEH and flashfm were run using the effect estimates, standard errors of these effect estimates, and in-sample LD. Some methods, including mvSuSiE, also accepted an additional input, the sample size (N), in which case we provided this as well. flashfm also required the “reference” allele frequencies, which in all our analyses were the minor allele frequencies.

### PAINTOR.

We ran PAINTOR [40] in only the 2-trait simulations. PAINTOR was designed to work with functional genomic annotation data, so to run PAINTOR we created a single “dummy” annotation in which all SNPs were assigned to this annotation (that is, all entries of the annotation matrix were set to 1). For all data sets, we asked PAINTOR to enumerate all possible configurations up to 2 causal SNPs. (In the 2-trait simulations, the true number of causal SNPs was always 2.) We did not use the “mcmc” option (-mcmc) because the outputted PIPs when using this option were all zero in our tests. (The same issue was reported in https://github.com/gkichaev/PAINTOR_V3.0/issues/5.) All other PAINTOR options were kept at their default settings. Note that PAINTOR does not accept N (the sample size) as input. Also note that PAINTOR assumes that both traits and effects are independent across traits (Table 1).

### flashfm.

We ran flashfm [41] in only the 2-trait simulations. We ran flashfm by calling function FLASHFMwithFINEMAP from R package flashfm (version 0.0.0.9000). This function internally calls FINEMAP [19, 42] (we used FINEMAP 1.4.1) with settings --sss --n-configs-top 1000 --n-causal-snps 10, which allows configurations of up to 10 causal SNPs. We ran flashfm with 4 CPUs (NCORES = 4). All other flashfm settings were kept at their defaults. The inputs to FLASHFMwithFINEMAP were the effect estimates, the standard errors of these effect estimates, minor allele frequencies, vector of trait means, and sample size N. Since Y was centered and standardized in the simulations, the vector of trait means was simply a vector of zeros of length R.

### CAFEH.

We ran CAFEH [43] on all data sets in Scenarios a and b. Specifically, we used the fit_cafeh_summary interface in CAFEH 1.0 installed with Python 3.7.4. The fit_cafeh_summary function accepts the following data inputs: effect estimates, standard errors of those estimates, LD matrix, and sample size N. When calling fit_cafeh_summary, all optional arguments were kept at the software defaults. CAFEH’s default setting for the upper limit on the number of single effects (denoted as “K” in the CAFEH model), is 10, which is the same default used in SuSiE and mvSuSiE. Note that CAFEH assumes that both traits and effects are independent across traits (Table 1).

For assessing performance of CAFEH PIPs and trait-wise PIPs (in CAFEH, these are called “study PIPs”), we called get_pip and get_study_pip.

CAFEH outputs credible sets without any filter on the purity of the CSs. Therefore, to make the CAFEH credible sets comparable to SuSiE and mvSuSiE credible sets, we filtered out CSs with purity less than 0.5.

Note that the two CAFEH summary data interfaces—fit_cafeh_summary and fit_cafeh_z—produce the same or very similar results when X is standardized, so we expect that both CAFEH summary-data interfaces would perform similarly when X is standardized. Both functions internally call function CAFEHSummary with the same LD matrix, but provide different effect estimates and standard errors of the effect estimates. Let $\hat{\beta }$ denote the vector of effect estimates (with one entry per SNP) and let $\overset{ˆ}{s}$ denote the vector of standard errors (also with one entry per SNP). If fit_cafeh_summary calls CAFEHSummary with inputs $\hat{\beta },\overset{ˆ}{s}$, and assuming X is standardized, then it can be shown that fit_cafeh_z calls CAFEHSummary with inputs $\sqrt{N}\hat{\beta },\sqrt{N}\overset{ˆ}{s}$. Since CAFEHSummary is invariant to rescaling of $\hat{\beta },\overset{ˆ}{s}$—that is, CAFEHSummary generates the same result with inputs $a\hat{\beta }$ and $a\hat{s}$ for any choice of scalar a > 0—it follows that fit_cafeh_summary and fit_cafeh_z also produce the same result when X is standardized. In practice, this invariance does not hold exactly since it requires that the prior on the effects also be appropriately rescaled, but empirically we have found that the CAFEH PIPs and posterior effect estimates are almost the same for different choices of a > 0. (See https://github.com/karltayeb/cafeh/blob/current_working_branch/notebooks/CAFEHS_scale_invariance.ipynb.)

### SuSiE.

We ran SuSiE in all simulations by calling function susie_rss from the susieR R package [4] (version 0.12.12). In each data set, we ran susie_rss once per trait. The susie_rss interface accepts different types of summary data; we provided z-scores, in-sample LD, and sample size N. For all simulations, we set L, the maximum number of non-zero effects, to 10. We also set L=10 for the 2-trait simulations even though there were never more than 2 causal SNPs in these simulations. We estimated the residual variance (estimate_residual_variance = TRUE), which is the recommended setting when the LD is estimated from the “in-sample” data. We set the maximum number of IBSS iterations to 1,000 (max_iter = 1000). The remaining optional arguments were kept at their defaults.

Since SuSiE analyzes each trait separately, it does not directly provide a systematic way to quantify evidence for a SNP being a cross-trait causal SNP. To quantify performance in this task and compare with mvSuSiE, we quantified the evidence for a cross-trait causal SNP using an *ad hoc* metric, the “maximum PIP”, defined as

(25) $$\text{max}-{\text{PIP}}_{j}≔\underset{r\in \left\{1,\ldots ,R\right\}}{\text{max}}{\text{PIP}}_{jr},$$

where ${\text{PIP}}_{jr}$ is the trait-wise PIP for SNP j obtained from the SuSiE analysis of trait r.

### mvSuSiE.

We ran mvSuSiE using the mvsusie_rss interface from the mvsusieR R package (version 0.0.3.0518, git commit id 9f28916). While susie_rss accepts a *vector* of z-scores, mvsusie_rss accepts a *matrix* of z-scores (specifically, a J×R matrix). In the simulations, we compared several mvSuSiE variants using different prior choices; for more details, see “Specifying the prior” above and Supplementary Fig. 1. (In the 2-trait simulations, we only used the canonical prior. This was a mixture of multivariate normals with K=7 components.) We also compared mvSuSiE with different settings of the residual covariance V; see “Estimating the residual variance matrix” above, and Supplementary Figures 2 and 9. In all cases, we ran mvsusie_rss with the following settings: L = 10, max_iter = 1000, estimate_prior_variance = TRUE, estimate_prior_method = “EM”, precompute_covariances = TRUE and n_thread = 4. We set L=10 for the 2-trait simulations even though there were never more than 2 causal SNPs in these simulations. All other options were kept at the default settings.

### Computing environment.

All analyses of the simulated data sets were run on Linux machines (Scientific Linux 7.4) with 4 Intel Xeon E5–2680v4 (“Broadwell”) processors, and with R 4.1.0 [44] linked to the OpenBLAS 0.3.13 optimized numerical libraries. At most 10 GB of memory was needed to perform a fine-mapping analysis of a single simulated data set using one of the methods. We used DSC version 0.4.3.5 to perform the simulations.

### Fine-mapping of UK Biobank blood cell traits using SuSiE and mvSuSiE.

We fit a mvSuSiE model to each fine-mapping data set—specifically the prepared z-scores matrix Z and LD matrix R—by calling mvsusie_rss from the mvsusieR package with the following settings: L = 10, N = 248980, precompute_covariances = TRUE, estimate_prior_variance = TRUE, estimate_prior_method = “EM”, max_iter = 1000 and n_thread = 1. We ran SuSiE on each data set {Z, R} separately for each trait (*i.e*., column of Z) by calling susie_rss with the following settings: n = 248980, L = 10, max_iter = 1000, estimate_prior_variance = TRUE, refine = TRUE. Any CSs returned by susie_rss or mvsusie_rss with purity less than 0.5 were removed.

### Enrichment analysis of regulatory annotations using GREGOR.

We performed enrichment analyses of the SuSiE and mvSuSiE blood cell trait fine-mapping results using GREGOR [45] (we used GREGOR version 1.4.0). In brief, GREGOR performs an enrichment analysis for given “positive set” of SNPs by calculating overlap with the given regulatory annotation, then estimates the probability of the observed overlap against its expectation using a set of “matched control SNPs”. We ran GREGOR with the following settings: pop = ‘EUR’, r2_threshold = 0.7, ld_window_size = 10000, min\_neighbor = 10, job_number = 10.

Although GREGOR provides p-values, we found some issues with these p-values (e.g., some exceeded 1). Therefore, for each annotation, we extracted the intermediate GREGOR outputs to get a 2 × 2 table of the SNP counts of inside and outside the annotation intersected with positive set and matched control set. We then used this 2 × 2 table to perform Fisher’s exact test and this was the final p-value reported. Additional details about the GREGOR analysis can be found in the 20231106_GREGOR_functional_enrichment.ipynb Jupyter notebook in the “mvarbvs” Zenodo repository (see URLs).

We assessed enrichment for a total of 19 regulatory genomic annotations, including: enhancer promoter regions and transcription factor binding sites [46]; genomic structural elements [47]; eQTLs in multiple tissues (based on different false discovery rates) [48]; RNA polymerase II binding in EPC-treated HESCs [49]; and binding intervals for specific transcription factors. The specific transcription factors included were promyelocytic leukemia zinc finger protein (PLZF), *FOSL2, NR2F2 and FOXO1* [49]. The BED annotation files are included in the Zenodo repository (see URLs).

Using these regulatory genomic annotations, we performed two sets of GREGOR enrichment analyses, one using the SuSiE fine-mapping results, and another set using the mvSuSiE fine-mapping results. We performed these enrichment analyses separately for the fine-mapping results for each blood cell trait, as well as the “global” (cross-trait) results. For mvSuSiE, we included a SNP in the cross-trait positive set if the SNP was included in at least one 95% CS and/or the global PIP was greater than 0.7. We included a SNP in the positive set for a given blood cell trait if the SNP was included in at least one 95% CS and the *lfsr* for the given trait was less 0.01.

For SuSiE, we included a SNP in the positive set for a given trait if the SNP was included in at least one 95% CS and/or the PIP was greater than 0.7. The SuSiE cross-trait positive set was defined as the union of the 16 positive sets from the SuSiE analyses of each of the 16 traits.

### Enrichment analysis of hematopoietic cell-types using gchromVAR.

We performed additional enrichment analyses of the SuSiE and mvSuSiE blood cell trait fine-mapping results using gchromVAR [31]. In brief, gchromVAR assesses overlap of the fine-mapped SNPs and regions of accessible chromatin, separately in different hematopoietic cell types. (This is actually a weighted overlap in which we have defined the weights as the SuSiE or mvSuSiE PIPs.) Each enrichment analysis was performed following the steps described in the gchromVAR R package vignette (we used version 0.3.2 of the gchromVAR R package). The z-scores returned by the computeWeightedDeviations function were then refined using the adaptive shrinkage method [22] (ashr package 2.2–57). The adaptive shrinkage posterior z-scores (posterior means divided by posterior standard deviations) and *lfsr* values were used to report the final enrichment results.

Similar to the GREGOR enrichment analyses, we performed two separate enrichment analyses with gchromVAR, one using the SuSiE results, and another using the mvSuSiE results. For SuSiE, we included a SNP for a given trait if the PIP > 0.01 for that trait. A total of 100,090 SNPs had PIP > 0.01 in at least one of the blood cell traits. For mvSuSiE, we included a SNP for a given trait if the global PIP > 0.01 and if the CS was significant for the given trait (*lfsr* < 0.01). A total of 39,884 SNPs had a global PIP > 0.01.

## Supplementary Material

Supplement 1

Supplement 2

Supplement 3

Supplement 4

Supplement 5

Supplement 6

Supplement 7

Supplement 8

## Figures and Tables

**Table 1 |:** Overview of available statistical methods for multi-trait fine-mapping.

method	upper limit on number of causal SNPs	data accepted			allows correlated traits	models effect sharing	sample runtimes			version
		summary	sufficient	CSs			2 traits	20 traits	software	
mvSuSiE	user-specified	yes	yes	yes	yes	Yes	41 s	2 min	R	9f28916
flashfm^†^ [20]	10	yes	yes	yes	yes	Yes	5 min	–	R	0.0.0.9000
MT-HESS [18]	no limit	no	no	no	yes	Yes	>1 day	–	R	1.99
BayesSUR [21]	no limit	no	no	no	yes	Yes	7 h	–	R	2.0-1
msCAVIAR [22]	user-specified	yes	no	no	no	Yes	>1 day	–	command-line	0.1
CAFEH [23]	user-specified	yes	yes	yes	no	No	20 s	37 s	Python	1.0
PAINTOR [19]	user-specified	yes	no	no	no	No	30 min	–	R	3.1
MFM^§^ [24]	user-specified	no	no	yes	no	No	–	–	R	0.2-1
HyPrColoc [25]	1	yes	no	yes^‡^	no	No	<1 s	<1 s	R	1.0
moloc [26]	1	yes	no	yes^‡^	no	No	<1 s	–	R	0.1.0

Sample runtimes were obtained by running on data sets with J=5,000 SNPs, N=250,000 individuals (relevant for methods that do not accept summary data), and R=2 or 20 traits. When possible, the upper limit on the number of causal SNPs, L, was set to 10. In our tests, PAINTOR ran for a very long time when allowing 3 or more causal SNPs, so we set L=2. (This was without the “MCMC” option, because at the time of our experiments the “MCMC” option produced unreasonable results.) moloc was computationally impractical with more than 4 traits [25]. See the Supplementary Note for details.

§ MFM is specific to multiple case-control traits with a shared set of controls.

† flashfm’s properties depend on the single-trait fine-mapping method; to illustrate, the properties shown here are for flashfm with FINEMAP [10]. flashfm with FINEMAP was limited to at most 5 traits. (Another flashfm interface allows up to 6 traits.)

‡ Calculation of CSs is trivial when limiting to at most 1 causal SNP.

## Data Availability

The genotype and phenotype data used in our analyses are available from UK Biobank. Association test statistics for the UK Biobank blood cell traits, results of the simulations, results of the blood cell trait fine-mapping analyses, and other results needed to reproduce the figures in the paper are also available online (see URLs).
