# Supplementary material for: Fast and flexible joint fine-mapping of multiple traits via the Sum of Single Effects model
Source: bioRxiv. 2024 Jun 18:2023.04.14.536893. Preprint. [Version 5] doi: 10.1101/2023.04.14.536893 (PMC10327118; doi:10.1101/2023.04.14.536893)
Supplement: Supplement 1 [file media-1.pdf]

Fast and flexible joint fine-mapping of multiple traits via the Sum of Single Effects model

Yuxin Zou
Department of Statistics, University of Chicago, Chicago, IL, USA
Regeneron Genetics Center, Regeneron Pharmaceuticals, Inc., Tarrytown, NY, USA
Peter Carbonetto
Department of Human Genetics, University of Chicago, Chicago, IL, USA
Dongyue Xie
Department of Statistics, University of Chicago, Chicago, IL, USA
Gao Wang*
Gertrude. H. Sergievsky Center, Department of Neurology, Columbia University, New York, NY, USA
Matthew Stephens*
Departments of Statistics and Human Genetics, University of Chicago, Chicago, IL, USA
**e-mail: wang.gao@columbia.edu; mstephens@uchicago.edu*

Supplementary Note

More details about Table 1

Here are more details about the columns in Table 1 in which we compared different statistical methods for multivariate fine-mapping:

- Upper limit on number of causal SNPs: "User-specified" means that the method requires the user to specify an upper limit on the number of causal SNPs allowed. "No limit" means that the method does not constrain the number of causal SNPs.

- Data accepted, summary and sufficient. Methods marked "yes" under "sufficient" should reproduce the same result as if the methods were provided with the full data. By contrast (non-sufficient) summary data approximate the summary statistics, and therefore yield results that are not expected to exactly reproduce the results that would be obtained with the full data. (All methods implicitly allow full data because summary statistics can always be computed from full data.) See [1] or the Online Methods ("Extension of posterior computation approach to work with summary data") for definitions of "summary data" and "sufficient data".

- CSs. Does the method compute credible sets (CS)? Note that calculation of CSs is trivial when limiting to at most 1 causal SNP.

- Allows correlated traits. Methods marked as "no" assume measurement error is independent across traits. Assuming independent errors is appropriate if different, non-overlapping samples were used to measure the different traits.

- Models effect sharing. Methods with a "no" in the column (implicitly or explicitly) assume that the effects of a SNP on different traits are independent (conditioned on the SNP having a nonzero effect on one or more traits). A "yes" means that the method can model correlations among the effects on different traits.

- Sample runtimes. Sample runtimes were obtained by running the method on a simulated data set with J = 5,000 SNPs, N = 250,000 individuals, and $R = 2$ or $R = 20$ traits. When the method accepted either full data or summary data, the summary-data version of the method was used. Note the sample size, N, should only affect the running time for methods that only

accept full data; it should not affect runtime of summary-data methods. When the method limits the number of causal SNPs, the upper limit was always set to 10 or the the largest acceptable value if this was less than 10. For PAINTOR, the upper limit was set to 2 because, in our tests, PAINTOR ran for a very long time when allowing 3 or more causal SNPs. See "Computing environment" for details about the computing environment used to obtain these runtimes.

- Software and version. The name of the software and the version number of the software that was used in our evaluations. For mvSuSiE, the git commit id was given instead of the version number.

Conventions for mathematical expressions

Here we summarize the notational conventions used in the main text and supplement. Matrices are written using bold, uppercase letters (e.g., $\boldsymbol{A}$), column vectors are written as bold, lowercase letters (e.g., $\boldsymbol{a}$), and scalars are written in plain font (e.g., a, A). For indexing, we usually use a capital letter to denote the total number of elements, and we use the corresponding lowercase symbol to denote the index; e.g., $j = 1, \ldots, J$. We use $\mathbb{R}$ to denote the real numbers and $\mathbb{R}^d$ for the set of real vectors of dimension d. We use Δ^d to denote the simplex on $\mathbb{R}^d$; that is, all $x \in \mathbb{R}^d$ such that $x_1 + \cdots + x_d = 1$, $x_i \geqslant 0$, $i = 1, \ldots, d$. We use $\mathbb{R}^{m \times n}$ to denote the set of all $m \times n$ matrices with real entries, $\mathbb{S}^n_{++}$ for the set of all $n \times n$ real, symmetric positive definite matrices, and $\mathbb{S}^n_{+}$ for the set of all $n \times n$ real, symmetric positive semi-definite matrices (this set may include matrices that are singular, or not invertible). We write the matrix transpose of $\boldsymbol{A}$ as $\boldsymbol{A}^\intercal$. For a square matrix $\boldsymbol{A}$, its inverse is $\boldsymbol{A}^{-1}$, its determinant is $\det \boldsymbol{A}$, also written as $|\boldsymbol{A}|$, the trace is $\mathrm{tr}(\boldsymbol{A})$, and $\boldsymbol{A}^\dagger$ denotes the Moore–Penrose inverse ("pseudoinverse") of $\boldsymbol{A}$. We use $\boldsymbol{I}_n$ for the $n \times n$ identity matrix, and we use $\mathbf{1}_n$ as a shorthand for a column vector of ones of length n. We use $\boldsymbol{a}^\intercal$ to denote a row vector. We denote the outer product of (column) vectors $\boldsymbol{a}$ and $\boldsymbol{b}$ as $\boldsymbol{a} \otimes \boldsymbol{b} := \boldsymbol{a}\boldsymbol{b}^\intercal$, and we denote the elementwise product of matrices $\boldsymbol{A}$ and $\boldsymbol{B}$ as $\boldsymbol{A} \circ \boldsymbol{B}$. Finally, we typically denote ordered or unordered sets using calligraphic letters (e.g., $\mathcal{A}$).

The multivariate simple regression model

The mvSuSiE model is based on a simple multivariate regression model with one variable which we refer to as the "multivariate simple regression model." This model is

$$\boldsymbol{Y} \sim MN_{N \times R}(\boldsymbol{x}\boldsymbol{b}^\intercal, \boldsymbol{I}_N, \boldsymbol{V}). \tag{1}$$

where $\boldsymbol{Y} \in \mathbb{R}^{N \times R}$ is a matrix of R observed responses in N samples, $\boldsymbol{x}$ is a vector of N observations for a single explanatory variable, $\boldsymbol{b} \in \mathbb{R}^R$ is the (unknown) vector of regression coefficients for the R responses, $\boldsymbol{V} \in \mathbb{S}^R_{++}$ is an invertible covariance matrix, and $MN_{n \times m}(\boldsymbol{M}, \boldsymbol{U}, \boldsymbol{V})$ is the matrix normal distribution [2, 3] with mean $\boldsymbol{M} \in \mathbb{R}^{n \times m}$ and covariance matrices $\boldsymbol{U}, \boldsymbol{V} \in \mathbb{S}^{m \times m}_{+}$. For now, this model does not include an intercept.

In the following, we give the expression for the likelihood for (1) and relate it to the more familiar multivariate normal distribution. The likelihood for the multivariate regression model (1) is

$$\begin{aligned} \ell(\boldsymbol{b}; \boldsymbol{x}, \boldsymbol{Y}, \boldsymbol{V}) &:= p(\boldsymbol{Y} \mid \boldsymbol{x}, \boldsymbol{b}, \boldsymbol{V}) \\ &= |2\pi \boldsymbol{V}|^{-N/2} \exp\big\{ -\tfrac{1}{2} \mathrm{tr}\big[\boldsymbol{V}^{-1}(\boldsymbol{Y} - \boldsymbol{x}\boldsymbol{b}^\intercal)^\intercal(\boldsymbol{Y} - \boldsymbol{x}\boldsymbol{b}^\intercal)\big]\big\}. \end{aligned} \tag{2}$$

Given $\boldsymbol{V}$, the least-squares estimate of $\boldsymbol{b}$, denoted by $\hat{\boldsymbol{b}}$, and its variance-covariance matrix, $\hat{\boldsymbol{S}}$, are

$$\hat{\boldsymbol{b}} = \tfrac{\boldsymbol{Y}^\intercal \boldsymbol{x}}{\boldsymbol{x}^\intercal \boldsymbol{x}} \tag{3}$$

$$\hat{\boldsymbol{S}} = \tfrac{\boldsymbol{V}}{\boldsymbol{x}^\intercal \boldsymbol{x}}. \tag{4}$$

Note that $\hat{\boldsymbol{b}}$ is also the value of $\boldsymbol{b}$ maximizing the likelihood (2). Using these quantities, the likelihood (2) can be rewritten as

$$\ell(\boldsymbol{b}; \boldsymbol{x}, \boldsymbol{Y}, \boldsymbol{V}) = |2\pi \boldsymbol{V}|^{-N/2} \exp\big\{ -\tfrac{1}{2}[\mathrm{tr}(\boldsymbol{V}^{-1}\boldsymbol{Y}^\intercal\boldsymbol{Y}) + (\boldsymbol{b} - \hat{\boldsymbol{b}})^\intercal \hat{\boldsymbol{S}}^{-1}(\boldsymbol{b} - \hat{\boldsymbol{b}}) - \hat{\boldsymbol{b}}^\intercal \hat{\boldsymbol{S}}^{-1}\hat{\boldsymbol{b}}]\big\}. \tag{5}$$

This expression is convenient because the terms involving $\boldsymbol{b}$ are multivariate normal up to a constant of proportionality, and in particular we have that

$$\ell(\boldsymbol{b}; \boldsymbol{x}, \boldsymbol{Y}, \boldsymbol{V}) \propto N_R(\boldsymbol{b}; \hat{\boldsymbol{b}}, \hat{\boldsymbol{S}}), \tag{6}$$

where $N_n(\boldsymbol{\theta}; \boldsymbol{\mu}, \boldsymbol{\Sigma})$ denotes the multivariate normal density at $\boldsymbol{\theta} \in \mathbb{R}^n$ with mean $\boldsymbol{\mu} \in \mathbb{R}^n$ and covariance $\boldsymbol{\Sigma} \in \mathbb{S}_+^n$.

Remark 1. Calculation of $\hat{\boldsymbol{b}}$ and $\hat{\boldsymbol{S}}$ only requires the summary statistics $\boldsymbol{x}^\intercal \boldsymbol{x}$, $\boldsymbol{Y}^\intercal \boldsymbol{x}$. Also, since the likelihood (6) only involves $\hat{\boldsymbol{b}}$ and $\hat{\boldsymbol{S}}$, the likelihood up to a constant of proportionality can also be computed with only these summary statistics.

The multivariate simple regression model with a normal prior

In the following proposition, we apply the above results for the multivariate simple regression model to a Bayesian multivariate simple regression model with a normal prior.

Proposition 1 (Bayesian multivariate simple regression with a normal prior). Consider the multivariate simple regression model (1) with a multivariate normal prior on the regression coefficients,

$$\boldsymbol{b} \mid \boldsymbol{S}_0 \sim N_R(\boldsymbol{0}, \boldsymbol{S}_0), \tag{7}$$

where $\boldsymbol{S}_0 \in \mathbb{S}_+^R$ is a (possibly singular) covariance matrix. The posterior of $\boldsymbol{b}$ is

$$\boldsymbol{b} \mid \boldsymbol{x}, \boldsymbol{Y}, \boldsymbol{V}, \boldsymbol{S}_0 \sim N_R(\boldsymbol{b}_1, \boldsymbol{S}_1), \tag{8}$$

where

$$\boldsymbol{b}_1 = \boldsymbol{S}_1 \hat{\boldsymbol{S}}^{-1} \hat{\boldsymbol{b}} \tag{9}$$

$$\boldsymbol{S}_1 = (\boldsymbol{S}_0^{-1} + \hat{\boldsymbol{S}}^{-1})^{-1}. \tag{10}$$

The Bayes Factor (BF) comparing this model against the null model ($\boldsymbol{b} = \boldsymbol{0}$) is

$$\begin{aligned} \mathrm{BF}(\boldsymbol{x}, \boldsymbol{Y}, \boldsymbol{V}, \boldsymbol{S}_0) &= \frac{p(\boldsymbol{Y} \mid \boldsymbol{x}, \boldsymbol{V}, \boldsymbol{S}_0)}{p(\boldsymbol{Y} \mid \boldsymbol{x}, \boldsymbol{V}, \boldsymbol{b} = \boldsymbol{0})} \\ &= \frac{\int \ell(\boldsymbol{b}; \boldsymbol{x}, \boldsymbol{Y}, \boldsymbol{V})\, p(\boldsymbol{b} \mid \boldsymbol{S}_0)\, d\boldsymbol{b}}{\ell(\boldsymbol{b} = \boldsymbol{0}; \boldsymbol{x}, \boldsymbol{Y}, \boldsymbol{V})}, \end{aligned} \tag{11}$$

reduces to

$$\mathrm{BF}(\boldsymbol{x}, \boldsymbol{Y}, \boldsymbol{V}, \boldsymbol{S}_0) = \frac{|\hat{\boldsymbol{S}}|^{1/2}}{|\boldsymbol{S}_0 + \hat{\boldsymbol{S}}|^{1/2}} \exp\{\tfrac{1}{2} \hat{\boldsymbol{b}}^\intercal \hat{\boldsymbol{S}}^{-1} \boldsymbol{S}_1 \hat{\boldsymbol{S}}^{-1} \hat{\boldsymbol{b}}\} \tag{12}$$

$$= \frac{|\hat{\boldsymbol{S}}|^{1/2}}{|\boldsymbol{S}_0 + \hat{\boldsymbol{S}}|^{1/2}} \exp\{\tfrac{1}{2} \boldsymbol{b}_1^\intercal \boldsymbol{S}_1^{-1} \boldsymbol{b}_1\}. \tag{13}$$

The same BF can also be equivalently expressed as a ratio of two multivariate normal densities,

$$\mathrm{BF}(\boldsymbol{x}, \boldsymbol{Y}, \boldsymbol{V}, \boldsymbol{S}_0) = \frac{N_R(\hat{\boldsymbol{b}}; \boldsymbol{0}, \boldsymbol{S}_0 + \hat{\boldsymbol{S}})}{N_R(\hat{\boldsymbol{b}}; \boldsymbol{0}, \hat{\boldsymbol{S}})}. \tag{14}$$

Remark 2. Since the data $\boldsymbol{x}, \boldsymbol{Y}$ only enter the expressions for the posterior mean $\boldsymbol{b}_1$ and posterior covariance $\boldsymbol{S}_1$ through $\hat{\boldsymbol{b}}$ and $\hat{\boldsymbol{S}}$, it follows that calculation of the posterior mean and covariance only requires summary statistics $\boldsymbol{x}^\intercal \boldsymbol{x}, \boldsymbol{Y}^\intercal \boldsymbol{x}$. Similarly, the BF (14) can be computed with only $\boldsymbol{x}^\intercal \boldsymbol{x}, \boldsymbol{Y}^\intercal \boldsymbol{x}$.

Updating a scaling factor in the prior. Suppose the prior covariance is parameterized as $\boldsymbol{S}_0 = \sigma_0^2 \boldsymbol{U}$, in which $\boldsymbol{U} \in \mathbb{S}_+^R$, $\sigma_0 \geqslant 0$. Here we assume $\boldsymbol{U}$ is a fixed parameter and we would like to estimate the scaling factor σ_0 by maximizing the likelihood,

$$\hat{\sigma}_0^2 := \underset{\sigma_0^2}{\operatorname{argmax}}\; p(\boldsymbol{Y} \mid \boldsymbol{x}, \boldsymbol{V}, \sigma_0^2 \boldsymbol{U}), \tag{15}$$

or, equivalently, by maximizing the Bayes Factor, which may be more convenient to compute,

$$\hat{\sigma}_0^2 = \underset{\sigma_0^2}{\operatorname{argmax}}\; \mathrm{BF}(\boldsymbol{x}, \boldsymbol{Y}, \boldsymbol{V}, \sigma_0^2 \boldsymbol{U}). \tag{16}$$

When $\boldsymbol{U}$ is invertible, the maximum-likelihood estimate $\hat{\sigma}_0^2$ can be computed using a simple EM algorithm [4], in which the M-step update is

$$\sigma_0^2 = \mathrm{tr}(\boldsymbol{U}^{-1} \mathbb{E}[\boldsymbol{b}\boldsymbol{b}^\intercal])/R. \tag{17}$$

The E-step then consists of computing the posterior second moment,

$$\mathbb{E}[\boldsymbol{b}\boldsymbol{b}^\intercal] = \boldsymbol{b}_1 \boldsymbol{b}_1^\intercal + \boldsymbol{S}_1, \tag{18}$$

in which the posterior mean $\boldsymbol{b}_1$ and posterior covariance $\boldsymbol{S}_1$ are given by (9, 10). The maximum-likelihood estimate $\hat{\sigma}_0^2$ is then recovered by iterating the E-step (18) and M-step (17) until convergence.

The update (17) requires that $\boldsymbol{U}$ be invertible. To allow for singular matrices, a more general M-step update is

$$\sigma_0^2 = \mathrm{tr}(\boldsymbol{U}^\dagger \mathbb{E}[\boldsymbol{b}\boldsymbol{b}^\intercal])/R'. \tag{19}$$

in which $R' \leqslant R$ is the rank of $\boldsymbol{U}$. Note (17) and (19) are equivalent when $\boldsymbol{U}$ is invertible; that is, when $R' = R$.

The multivariate simple regression model with an intercept

Now we extend the multivariate simple regression model (1) to include an intercept. We show that including an intercept in the model is equivalent to "centering" $\boldsymbol{x}$ and the columns of $\boldsymbol{Y}$ so that they all have means of zero. More precisely, centering is equivalent to integrating out the intercept with respect to an (improper) uniform prior on the intercept. This is the multivariate generalization of the result for univariate regression given in [5]. This result is summarized in Proposition 2.

The multivariate simple regression model with an intercept is

$$\boldsymbol{Y} \sim MN_{N \times R}(\boldsymbol{1}_N \boldsymbol{\mu}^\intercal + \boldsymbol{x}\boldsymbol{b}^\intercal, \boldsymbol{I}_N, \boldsymbol{V}), \tag{20}$$

in which $\boldsymbol{\mu} \in \mathbb{R}^R$ is the (unknown) intercept. The likelihood of $\boldsymbol{\mu}, \boldsymbol{b}$ under this model is

$$\begin{aligned} \ell(\boldsymbol{\mu}, \boldsymbol{b}; \boldsymbol{x}, \boldsymbol{Y}, \boldsymbol{V}) &:= p(\boldsymbol{Y} \mid \boldsymbol{x}, \boldsymbol{\mu}, \boldsymbol{b}, \boldsymbol{V}) \\ &= |2\pi \boldsymbol{V}|^{-N/2} \exp\{-\tfrac{1}{2}\mathrm{tr}[\boldsymbol{V}^{-1}(\boldsymbol{Y} - \boldsymbol{1}\boldsymbol{\mu}^\intercal - \boldsymbol{x}\boldsymbol{b}^\intercal)^\intercal(\boldsymbol{Y} - \boldsymbol{1}\boldsymbol{\mu}^\intercal - \boldsymbol{x}\boldsymbol{b}^\intercal)]\}. \end{aligned} \tag{21}$$

Proposition 2 (Multivariate simple regression with an intercept). Consider the multivariate simple regression model with an intercept (20). The least-squares estimate of $\boldsymbol{\mu}$—which is also the value of $\boldsymbol{\mu}$ maximizing the likelihood (21)—and its covariance matrix are

$$\hat{\boldsymbol{\mu}} = \bar{\boldsymbol{y}} - \bar{x}\boldsymbol{b} \tag{22}$$

$$\hat{\boldsymbol{S}}_\mu = \tfrac{1}{N}\boldsymbol{V}, \tag{23}$$

in which $\bar{x} := \frac{1}{N}\boldsymbol{x}^\intercal \boldsymbol{1}_N = \frac{1}{N}\sum_{i=1}^N x_i$ is the sample mean of $\boldsymbol{x}$, and $\bar{\boldsymbol{y}} := \frac{1}{N}\boldsymbol{Y}^\intercal \boldsymbol{1}_N$ is the vector containing the column means of $\boldsymbol{Y}$.

The profile likelihood for $\boldsymbol{b}$ is

$$\begin{aligned} \ell^*(\boldsymbol{b}; \boldsymbol{x}, \boldsymbol{Y}, \boldsymbol{V}) &:= \max_{\boldsymbol{\mu}} \ell(\boldsymbol{\mu}, \boldsymbol{b}; \boldsymbol{x}, \boldsymbol{Y}, \boldsymbol{V}) \\ &= \ell(\boldsymbol{b}; \tilde{\boldsymbol{x}}, \tilde{\boldsymbol{Y}}, \boldsymbol{V}), \end{aligned} \tag{24}$$

in which $\tilde{\boldsymbol{x}} := \boldsymbol{x} - \bar{x}\mathbf{1}_N$ and $\tilde{\boldsymbol{Y}} := \boldsymbol{Y} - \mathbf{1}_N\bar{\boldsymbol{y}}^\intercal$ are the centered $\boldsymbol{x}$ and $\boldsymbol{Y}$. In other words, the profile likelihood for the multivariate simple regression with an intercept is the same as the likelihood for the multivariate simple regression *without* an intercept if we first center $\boldsymbol{x}$ and $\boldsymbol{Y}$. Centering $\boldsymbol{x}$ and $\boldsymbol{Y}$ is therefore equivalent to including an intercept in the multivariate regression and estimating the intercept by maximum-likelihood.

Next, consider Bayesian calculations for $\boldsymbol{\mu}$ with a multivariate normal prior, $\boldsymbol{\mu} \mid \boldsymbol{S}_{0\mu} \sim N_R(0, \boldsymbol{S}_{0\mu})$, in which $\boldsymbol{S}_{0\mu} \in \mathbb{S}_+^R$ is a (possibly singular) covariance matrix. The posterior for $\boldsymbol{\mu}$ conditioned on $\boldsymbol{b}$ is

$$\boldsymbol{\mu} \mid \boldsymbol{x}, \boldsymbol{Y}, \boldsymbol{V}, \boldsymbol{S}_{0\mu}, \boldsymbol{b} \sim N_R(\boldsymbol{\mu}_1, \boldsymbol{S}_{1\mu}), \tag{25}$$

where

$$\boldsymbol{\mu}_1 = \boldsymbol{S}_{1\mu}\hat{\boldsymbol{S}}_\mu^{-1}\hat{\boldsymbol{\mu}} \tag{26}$$
$$\boldsymbol{S}_{1\mu} = (\boldsymbol{S}_{0\mu}^{-1} + \hat{\boldsymbol{S}}_\mu^{-1})^{-1}. \tag{27}$$

The marginal likelihood obtained by averaging over the intercept is

$$\begin{aligned}\ell^\star(\boldsymbol{b}; \boldsymbol{x}, \boldsymbol{Y}, \boldsymbol{V}, \boldsymbol{S}_{0\mu}) &:= \textstyle\int \ell(\boldsymbol{\mu}, \boldsymbol{b}; \boldsymbol{x}, \boldsymbol{Y}, \boldsymbol{V})\, p(\boldsymbol{\mu} \mid \boldsymbol{S}_{0\mu})\, d\boldsymbol{\mu} \\ &= |2\pi\boldsymbol{V}|^{-N/2}|\boldsymbol{S}_{0\mu}^{-1}\boldsymbol{S}_{1\mu}|^{1/2}\exp\{\tfrac{1}{2}\boldsymbol{\mu}_1^\intercal\boldsymbol{S}_{1\mu}^{-1}\boldsymbol{\mu}_1 - \tfrac{1}{2}\mathrm{tr}[\boldsymbol{V}^{-1}(\boldsymbol{Y} - \boldsymbol{x}\boldsymbol{b}^\intercal)^\intercal(\boldsymbol{Y} - \boldsymbol{x}\boldsymbol{b}^\intercal)]\}.\end{aligned} \tag{28}$$

In the special case of an (improper) uniform prior on $\boldsymbol{\mu}$, defined as $\boldsymbol{\mu} \sim N_R(0, \boldsymbol{S}_{0\mu})$ with $\boldsymbol{S}_{0\mu}^{-1} \to 0$, the posterior mean reduces to the least-squares estimate $\boldsymbol{\mu}_1 = \hat{\boldsymbol{\mu}}$, with covariance matrix $\boldsymbol{S}_{1\mu} = \hat{\boldsymbol{S}}_\mu$, and the marginal likelihood (28) simplifies to

$$\begin{aligned}\ell^\star(\boldsymbol{b}; \boldsymbol{x}, \boldsymbol{Y}, \boldsymbol{V}, \boldsymbol{S}_{0\mu}) &= |2\pi\boldsymbol{V}|^{-N/2}|\boldsymbol{S}_{0\mu}^{-1}\hat{\boldsymbol{S}}_\mu|^{1/2}\exp\left\{\tfrac{1}{2}\hat{\boldsymbol{\mu}}^\intercal\hat{\boldsymbol{S}}_\mu^{-1}\hat{\boldsymbol{\mu}} - \tfrac{1}{2}\mathrm{tr}\big[\boldsymbol{V}^{-1}(\boldsymbol{Y} - \boldsymbol{x}\boldsymbol{b}^\intercal)^\intercal(\boldsymbol{Y} - \boldsymbol{x}\boldsymbol{b}^\intercal)\big]\right\} \\ &= |\boldsymbol{S}_{0\mu}^{-1}\hat{\boldsymbol{S}}_\mu|^{1/2} \times \ell(\boldsymbol{b}; \tilde{\boldsymbol{x}}, \tilde{\boldsymbol{Y}}, \boldsymbol{V}),\end{aligned} \tag{29}$$

in which $\tilde{\boldsymbol{x}} := \boldsymbol{x} - \bar{x}\mathbf{1}$ and $\tilde{\boldsymbol{Y}} := \boldsymbol{Y} - \mathbf{1}\bar{\boldsymbol{y}}^\intercal$ are the centered $\boldsymbol{x}$ and $\boldsymbol{Y}$. In other words, the marginal likelihood for multivariate simple regression with an intercept (20), when we use an improper uniform prior for the intercept, is the same (up to a constant of proportionality) as the likelihood for multivariate simple regression *without* an intercept (1) after first centering $\boldsymbol{x}$ and $\boldsymbol{Y}$.

See below for a proof of this result.

Remark 3. To account for an intercept when computing posterior quantities and BFs for the multivariate simple regression model (1), $\boldsymbol{x}$ and $\boldsymbol{Y}$ should be centered before computing the summary statistics; that is, the summary statistics should be $\tilde{\boldsymbol{x}}^\intercal\tilde{\boldsymbol{x}}$ and $\tilde{\boldsymbol{Y}}^\intercal\tilde{\boldsymbol{x}}$. See [1] for how to center summary statistics if they are not centered.

The Bayesian multivariate simple regression model with a mixture prior

Here we extend the Bayesian multivariate simple regression model with a normal prior to a model with a mixture-of-normals prior,

$$\boldsymbol{b} \mid \mathcal{S}_0, \boldsymbol{\omega} \sim \sum_{k=1}^K \omega_k N_R(\mathbf{0}, \boldsymbol{S}_{0k}), \tag{30}$$

in which $\mathcal{S}_0 := \{\boldsymbol{S}_{01}, \ldots, \boldsymbol{S}_{0K}\}$, each $\boldsymbol{S}_{0k} \in \mathbb{S}_+^R$ is a (possibly singular) covariance matrix, and $\boldsymbol{\omega} := (\omega_1, \ldots, \omega_K) \in \Delta^K$ are the mixture weights. The normal prior (7) is as a special case of (30) when $K = 1$.

To facilitate derivation of the posterior computations, we introduce the following data augmentation that recovers (30) after integrating over a latent random variable $\xi \in \{1, \ldots, K\}$,

$$\begin{aligned}[m]p(\xi = k \mid \boldsymbol{\omega}) &= \omega_k \\ \boldsymbol{b} \mid \mathcal{S}_0, \xi = k &\sim N_R(\mathbf{0}, \boldsymbol{S}_{0k}).\end{aligned} \tag{31}$$

This augmented model allows us to reuse the posterior computations from the simpler models; in particular, posterior computations conditioned on ξ reduce to computations for the Bayesian multivariate regression model with a normal prior, which we state formally in the following proposition.

PROPOSITION 3. Given $\mathcal{S}_0$ and $\boldsymbol{\omega}$, the Bayes factor comparing this model against the null model ($\boldsymbol{b} = \boldsymbol{0}$) is

$$\begin{aligned}\mathrm{BF}^{\mathsf{mix}}(\boldsymbol{x}, \boldsymbol{Y}, \boldsymbol{V}, \mathcal{S}_0, \boldsymbol{\omega}) &= \frac{p(\boldsymbol{Y} \mid \boldsymbol{x}, \boldsymbol{V}, \mathcal{S}_0, \boldsymbol{\omega})}{p(\boldsymbol{Y} \mid \boldsymbol{x}, \boldsymbol{V}, \boldsymbol{b} = \boldsymbol{0})} \\ &= \sum_{k=1}^{K} \omega_k \mathrm{BF}(\boldsymbol{x}, \boldsymbol{Y}, \boldsymbol{V}, \boldsymbol{S}_{0k}), \end{aligned} \tag{32}$$

where the expressions for the individual BFs in the sum are given in Proposition 1. The posterior distribution of $\boldsymbol{b}$ is a mixture of normals,

$$\boldsymbol{b} \mid \boldsymbol{x}, \boldsymbol{Y}, \boldsymbol{V}, \mathcal{S}_0, \boldsymbol{\omega} \sim \sum_{k=1}^{K} \omega_{1k} N_R(\boldsymbol{b}_{1k}, \boldsymbol{S}_{1k}), \tag{33}$$

in which $\boldsymbol{b}_{1k}$ and $\boldsymbol{S}_{1k}$ are the posterior mean and covariance of $\boldsymbol{b}$ conditioned on $\xi = k$, given by (9) and (10), respectively, after substituting $\boldsymbol{S}_0$ with $\boldsymbol{S}_{0k}$,

$$\boldsymbol{b}_{1k} := \boldsymbol{b}_{1k}(\boldsymbol{x}, \boldsymbol{Y}, \boldsymbol{V}, \mathcal{S}_0, \boldsymbol{\omega}) = \boldsymbol{S}_{1k} \hat{\boldsymbol{S}}^{-1} \hat{\boldsymbol{b}} \tag{34}$$

$$\boldsymbol{S}_{1k} := \boldsymbol{S}_{1k}(\boldsymbol{x}, \boldsymbol{Y}, \boldsymbol{V}, \mathcal{S}_0, \boldsymbol{\omega}) = (\boldsymbol{S}_{0k}^{-1} + \hat{\boldsymbol{S}}^{-1})^{-1}, \tag{35}$$

and the posterior mixture assignment probabilities ("responsibilities") are

$$\begin{aligned}\omega_{1k} &:= \omega_{1k}(\boldsymbol{x}, \boldsymbol{Y}, \boldsymbol{V}, \mathcal{S}_0, \boldsymbol{\omega}) \\ &= p(\xi = k \mid \boldsymbol{x}, \boldsymbol{Y}, \boldsymbol{V}, \mathcal{S}_0, \boldsymbol{\omega}) \\ &= \frac{\omega_k \mathrm{BF}(\boldsymbol{x}, \boldsymbol{Y}, \boldsymbol{V}, \boldsymbol{S}_{0k})}{\sum_{k'=1}^{K} \omega_{k'} \mathrm{BF}(\boldsymbol{x}, \boldsymbol{Y}, \boldsymbol{V}, \boldsymbol{S}_{0k'})}. \end{aligned} \tag{36}$$

The posterior mean and covariance of $\boldsymbol{b}$ are

$$\boldsymbol{b}_1^{\mathsf{mix}} := \boldsymbol{b}_1^{\mathsf{mix}}(\boldsymbol{x}, \boldsymbol{Y}, \boldsymbol{V}, \mathcal{S}_0, \boldsymbol{\omega}) = \textstyle\sum_{k=1}^{K} \omega_{1k} \boldsymbol{b}_{1k} \tag{37}$$

$$\boldsymbol{S}_1^{\mathsf{mix}} := \boldsymbol{S}_1^{\mathsf{mix}}(\boldsymbol{x}, \boldsymbol{Y}, \boldsymbol{V}, \mathcal{S}_0, \boldsymbol{\omega}) = \textstyle\sum_{k=1}^{K} \omega_{1k}(\boldsymbol{b}_{1k} \boldsymbol{b}_{1k}^{\mathsf{T}} + \boldsymbol{S}_{1k}) - \boldsymbol{b}_1^{\mathsf{mix}} (\boldsymbol{b}_1^{\mathsf{mix}})^{\mathsf{T}}. \tag{38}$$

From the above remarks, the posterior quantities and Bayes factors for this model can be computed using the summary statistics $\boldsymbol{x}^{\mathsf{T}}\boldsymbol{x}$, $\boldsymbol{Y}^{\mathsf{T}}\boldsymbol{x}$ instead of using the full data $\boldsymbol{x}, \boldsymbol{Y}$. To formalize these computations with summary statistics, we introduce notation for Bayes factors and posteriors in terms of summary statistics:

$$\boldsymbol{b}_{1k}^{\mathsf{ss}}(\boldsymbol{x}^{\mathsf{T}}\boldsymbol{x}, \boldsymbol{Y}^{\mathsf{T}}\boldsymbol{x}, \boldsymbol{V}, \mathcal{S}_0, \boldsymbol{\omega}) := \boldsymbol{b}_{1k}(\boldsymbol{x}, \boldsymbol{Y}, \boldsymbol{V}, \mathcal{S}_0, \boldsymbol{\omega}) \tag{39}$$

$$\boldsymbol{S}_{1k}^{\mathsf{ss}}(\boldsymbol{x}^{\mathsf{T}}\boldsymbol{x}, \boldsymbol{Y}^{\mathsf{T}}\boldsymbol{x}, \boldsymbol{V}, \mathcal{S}_0, \boldsymbol{\omega}) := \boldsymbol{S}_{1k}(\boldsymbol{x}, \boldsymbol{Y}, \boldsymbol{V}, \mathcal{S}_0, \boldsymbol{\omega}) \tag{40}$$

$$\omega_{1k}^{\mathsf{ss}}(\boldsymbol{x}^{\mathsf{T}}\boldsymbol{x}, \boldsymbol{Y}^{\mathsf{T}}\boldsymbol{x}, \boldsymbol{V}, \mathcal{S}_0, \boldsymbol{\omega}) := \omega_{1k}(\boldsymbol{x}, \boldsymbol{Y}, \boldsymbol{V}, \mathcal{S}_0, \boldsymbol{\omega}) \tag{41}$$

$$\mathrm{BF}^{\mathsf{mix\text{-}ss}}(\boldsymbol{x}^{\mathsf{T}}\boldsymbol{x}, \boldsymbol{Y}^{\mathsf{T}}\boldsymbol{x}, \boldsymbol{V}, \mathcal{S}_0, \boldsymbol{\omega}) := \mathrm{BF}^{\mathsf{mix}}(\boldsymbol{x}, \boldsymbol{Y}, \boldsymbol{V}, \mathcal{S}_0, \boldsymbol{\omega}). \tag{42}$$

Updating a scaling factor in the prior. Similar to above, here we consider a special case of the mixture-of-normals prior in which the prior covariances are parameterized as $\boldsymbol{S}_{0k} = \sigma_0^2 \boldsymbol{U}_k$, $\boldsymbol{U}_k \in \mathbb{S}_+^R$, $k = 1, \ldots, K$, $\sigma_0 \geqslant 0$. We assume the $\boldsymbol{U}_1, \ldots, \boldsymbol{U}_K$ are fixed parameters and we would like to estimate the scaling factor σ_0 by maximizing the likelihood,

$$\hat{\sigma}_0^2 := \underset{\sigma_0^2}{\operatorname{argmax}}\; p(\boldsymbol{Y} \mid \boldsymbol{X}, \boldsymbol{V}, \mathcal{S}_0, \boldsymbol{\omega}), \tag{43}$$

This is the same as maximizing the BF since the denominator in the BF does not depend on σ_0, and the BF may be more convenient to compute:

$$\hat{\sigma}_0^2 = \underset{\sigma_0^2}{\operatorname{argmax}}\ \mathrm{BF}^{\mathsf{mix}}(\boldsymbol{x}, \boldsymbol{Y}, \boldsymbol{V}, \mathcal{S}_0, \boldsymbol{\omega}). \tag{44}$$

Again taking a simple EM approach to computing the maximum-likelihood estimate, the M-step update allowing for singular matrices is

$$\sigma_0^2 = \sum_{k=1}^{K} \frac{\omega_{1k}}{R_k} \times \mathrm{tr}(\boldsymbol{U}_k^{\dagger} \mathbb{E}[\boldsymbol{b}\boldsymbol{b}^{\mathsf{T}} \mid \xi = k]), \tag{45}$$

in which the E-step involves computing the posterior probabilities ω_{1k} and the posterior second moments,

$$\mathbb{E}[\boldsymbol{b}\boldsymbol{b}^{\mathsf{T}} \mid \xi = k] = \boldsymbol{b}_{1k}\boldsymbol{b}_{1k}^{\mathsf{T}} + \boldsymbol{S}_{1k}. \tag{46}$$

Here, $R_k \leqslant R$ denotes the rank of $\boldsymbol{U}_k$.

The multivariate single effect regression model

The single effect regression (SER) model is a multiple regression model in which exactly one of the regression coefficients is non-zero [6]. Here we define the multivariate single effect regression (MSER) model, which extends the SER model to the multivariate setting, and forms the basis for mvSuSiE.

The MSER model is

$$\begin{aligned} [m]\boldsymbol{Y} &\sim MN_{N\times R}(\boldsymbol{X}\boldsymbol{B}, \boldsymbol{I}_N, \boldsymbol{V}) \\ \boldsymbol{B} &= \boldsymbol{\gamma} \otimes \boldsymbol{b}, \\ \boldsymbol{\gamma} &\sim \mathrm{Multinom}(1, \boldsymbol{\pi}), \\ \boldsymbol{b} &\sim \sum_{k=1}^{K} \omega_k N_R(\boldsymbol{0}, \boldsymbol{S}_{0k}), \end{aligned} \tag{47}$$

in which $\boldsymbol{Y} \in \mathbb{R}^{N\times R}$ is a matrix storing N observations about R regression outcomes, $\boldsymbol{X} \in \mathbb{R}^{N\times J}$ is a matrix storing N observations about J regression variables, $\boldsymbol{B} \in \mathbb{R}^{J\times R}$ is a matrix of regression coefficients for the J variables and R outcomes, $\boldsymbol{b} \in \mathbb{R}^R$ is a vector of "single effect" regression coefficients, $\boldsymbol{V} \in \mathbb{S}_{++}^R$ is an invertible covariance matrix, $\boldsymbol{S}_{01}, \ldots, \boldsymbol{S}_{0K} \in \mathbb{S}_{+}^R$ are (possibly singular) prior covariance matrices, $\boldsymbol{\gamma} \in \{0,1\}^J$ is a random variable indicating which of the J variables explains the multivariate outcome (exactly one element is one, and the remaining are zero), $\boldsymbol{\pi} \in \Delta^J$ specifies the probabilities in the multinomial prior on $\boldsymbol{\gamma}$, $\boldsymbol{\omega} \in \Delta^K$ is a vector specifying the weights in the mixture-of-normals prior on $\boldsymbol{b}$, and Multinom$(m, \boldsymbol{\pi})$ denotes the multinomial distribution for m random trials with probabilities $\boldsymbol{\pi}$. Because $\boldsymbol{\gamma}$ is drawn from the multinomial distribution with a single random trial, $\boldsymbol{\gamma}$ has exactly one non-zero element; that is, $\gamma_k = 1$ for some k, and $\gamma_{k'} = 0$ for all other $k' \neq k$. From this property, it follows that the matrix $\boldsymbol{B}$ has at most one row containing non-zero elements, and this row is given by $\boldsymbol{b}$. The MSER model recovers the SER model when $R = 1$, $K = 1$, $\omega_1 = 1$ and $\boldsymbol{S}_{01} = 1$.

The posterior distribution of $\boldsymbol{B}, \boldsymbol{\gamma}$ is summarized by the following proposition.

Proposition 4. Let $\mathcal{S}_0 = \{\boldsymbol{S}_{01}, \ldots, \boldsymbol{S}_{0K}\}$ denote the K prior covariance matrices. Under the MSER model (47), the posterior distribution of $\boldsymbol{B} = \boldsymbol{\gamma}\otimes\boldsymbol{b}$ given parameter settings $\Theta := \{\boldsymbol{V}, \mathcal{S}_0, \boldsymbol{\omega}, \boldsymbol{\pi}\}$ is given by

$$\begin{aligned} [m]\boldsymbol{\gamma} \mid \boldsymbol{X}, \boldsymbol{Y}, \Theta &\sim \mathrm{Multinom}(1, \boldsymbol{\alpha}) \\ \boldsymbol{b} \mid \boldsymbol{X}, \boldsymbol{Y}, \Theta, \gamma_j = 1 &\sim \sum_{k=1}^{K} \omega_{1jk} N_R(\boldsymbol{b}_{1jk}, \boldsymbol{S}_{1jk}), \end{aligned} \tag{48}$$

where $\boldsymbol{\alpha} = (\alpha_1, \ldots, \alpha_J)$ is the vector of posterior inclusion probabilities (PIPs), which can be written using the Bayes factors (32) for the Bayesian multivariate simple regression model with a mixture of

normals prior,

$$\begin{aligned}\alpha_j &:= \alpha_j(\boldsymbol{x}_j, \boldsymbol{Y}, \boldsymbol{V}, \mathcal{S}_0, \boldsymbol{\omega}) \\ &= \Pr(\gamma_j = 1 \mid \boldsymbol{X}, \boldsymbol{Y}, \Theta) \\ &= \frac{\pi_j \mathrm{BF}^{\mathsf{mix}}(\boldsymbol{x}_j, \boldsymbol{Y}, \boldsymbol{V}, \mathcal{S}_0, \boldsymbol{\omega})}{\sum_{j'=1}^{J} \pi_{j'} \mathrm{BF}^{\mathsf{mix}}(\boldsymbol{x}_{j'}, \boldsymbol{Y}, \boldsymbol{V}, \mathcal{S}_0, \boldsymbol{\omega})},\end{aligned} \tag{49}$$

and where the means $\boldsymbol{b}_{1jk}$, variances $\boldsymbol{S}_{1jk}$ and posterior mixture weights ω_{1jk} are given by

$$\boldsymbol{b}_{1jk} = \boldsymbol{b}_{1k}(\boldsymbol{x}_j, \boldsymbol{Y}, \boldsymbol{V}, \mathcal{S}_0, \boldsymbol{\omega}) \tag{50}$$

$$\boldsymbol{S}_{1jk} = \boldsymbol{S}_{1k}(\boldsymbol{x}_j, \boldsymbol{Y}, \boldsymbol{V}, \mathcal{S}_0, \boldsymbol{\omega}) \tag{51}$$

$$\omega_{1jk} = \omega_{1k}(\boldsymbol{x}_j, \boldsymbol{Y}, \boldsymbol{V}, \mathcal{S}_0, \boldsymbol{\omega}), \tag{52}$$

using the definitions of ω_{1k}, $\boldsymbol{b}_{1k}$ and $\boldsymbol{S}_{1k}$ in eqs. 34–36. The posterior mean and covariance of $\boldsymbol{b}$ conditioned on $\boldsymbol{\gamma}$ are

$$\mathbb{E}[\boldsymbol{b} \mid \gamma_j = 1] = \boldsymbol{b}_1^{\mathsf{mix}}(\boldsymbol{x}_j, \boldsymbol{Y}, \boldsymbol{V}, \mathcal{S}_0, \boldsymbol{\omega}) \tag{53}$$

$$\mathrm{Cov}[\boldsymbol{b} \mid \gamma_j = 1] = \boldsymbol{S}_1^{\mathsf{mix}}(\boldsymbol{x}_j, \boldsymbol{Y}, \boldsymbol{V}, \mathcal{S}_0, \boldsymbol{\omega}), \tag{54}$$

using definitions (37,38), and therefore the posterior mean of $\boldsymbol{B}$ is

$$\mathbb{E}[\boldsymbol{B}] = \begin{bmatrix} \alpha_1 \boldsymbol{b}_1^{\mathsf{mix}}(\boldsymbol{x}_1, \boldsymbol{Y}, \boldsymbol{V}, \mathcal{S}_0, \boldsymbol{\omega})^{\mathsf{T}} \\ \vdots \\ \alpha_J \boldsymbol{b}_1^{\mathsf{mix}}(\boldsymbol{x}_J, \boldsymbol{Y}, \boldsymbol{V}, \mathcal{S}_0, \boldsymbol{\omega})^{\mathsf{T}} \end{bmatrix}. \tag{55}$$

For describing the model fitting algorithms (below), we define a function, MSER, that returns the posterior distribution of $\boldsymbol{b}, \boldsymbol{\gamma}$ under the MSER model given the data $\boldsymbol{X}, \boldsymbol{Y}$ and the model parameters Θ:

$$\mathrm{MSER}(\boldsymbol{X}, \boldsymbol{Y}; \Theta) := (\boldsymbol{\alpha}, \boldsymbol{\Omega}_1, \mathcal{B}_1, \mathcal{S}_1), \tag{56}$$

in which $\boldsymbol{\alpha} = (\alpha_1, \ldots, \alpha_J)$ is the vector of PIPs (49), $\boldsymbol{\Omega}_1$ is the $J \times R$ matrix of posterior mixture weights ω_{1jk} (52), $\mathcal{B}_1$ is the set of posterior means $\boldsymbol{b}_{1jk}$ (50) for all j, k, and $\mathcal{S}_1$ is the set of posterior covariances $\boldsymbol{S}_{1jk}$ (51) for all j, k.

The multivariate single effect regression model with summary statistics. From the above remarks, $\boldsymbol{X}^{\mathsf{T}}\boldsymbol{X}$ and $\boldsymbol{X}^{\mathsf{T}}\boldsymbol{Y}$ are sufficient to compute the posterior distribution of $\boldsymbol{b}, \boldsymbol{\gamma}$. (More precisely, only the diagonal elements of $\boldsymbol{X}^{\mathsf{T}}\boldsymbol{X}$ are necessary to compute the posterior distribution of $\boldsymbol{b}, \gamma$.) To formalize these computations with summary statistics, we also define Bayes factors and posterior quantities in terms of summary statistics:

$$\boldsymbol{b}_{1jk}^{\mathsf{ss}} := \boldsymbol{b}_{1k}^{\mathsf{ss}}(\boldsymbol{x}_j^{\mathsf{T}}\boldsymbol{x}_j, \boldsymbol{Y}^{\mathsf{T}}\boldsymbol{x}_j, \boldsymbol{V}, \mathcal{S}_0, \boldsymbol{\omega}) \tag{57}$$

$$\boldsymbol{S}_{1jk}^{\mathsf{ss}} := \boldsymbol{S}_{1k}^{\mathsf{ss}}(\boldsymbol{x}_j^{\mathsf{T}}\boldsymbol{x}_j, \boldsymbol{Y}^{\mathsf{T}}\boldsymbol{x}_j, \boldsymbol{V}, \mathcal{S}_0, \boldsymbol{\omega}) \tag{58}$$

$$\omega_{1jk}^{\mathsf{ss}} := \omega_{1k}^{\mathsf{ss}}(\boldsymbol{x}_j^{\mathsf{T}}\boldsymbol{x}_j, \boldsymbol{Y}^{\mathsf{T}}\boldsymbol{x}_j, \boldsymbol{V}, \mathcal{S}_0, \boldsymbol{\omega}), \tag{59}$$

and the PIPs,

$$\alpha_j^{\mathsf{ss}} = \frac{\mathrm{BF}^{\mathsf{mix\text{-}ss}}(\boldsymbol{x}_j^{\mathsf{T}}\boldsymbol{x}_j, \boldsymbol{Y}^{\mathsf{T}}\boldsymbol{x}_j, \boldsymbol{V}, \mathcal{S}_0, \boldsymbol{\omega})}{\sum_{j'=1}^{J} \pi_{j'} \mathrm{BF}^{\mathsf{mix\text{-}ss}}(\boldsymbol{x}_{j'}^{\mathsf{T}}\boldsymbol{x}_{j'}, \boldsymbol{Y}^{\mathsf{T}}\boldsymbol{x}_{j'}, \boldsymbol{V}, \mathcal{S}_0, \boldsymbol{\omega})}. \tag{60}$$

To describe the model fitting algorithms that work with summary statistics, we define a function, MSER-ss, that returns the posterior distribution of $\boldsymbol{b}, \boldsymbol{\gamma}$ under the MSER model given the summary statistics $\boldsymbol{X}^{\mathsf{T}}\boldsymbol{X}, \boldsymbol{X}^{\mathsf{T}}\boldsymbol{Y}$ and the model parameters Θ:

$$\text{MSER-ss}(\boldsymbol{X}^{\mathsf{T}}\boldsymbol{X}, \boldsymbol{X}^{\mathsf{T}}\boldsymbol{Y}; \Theta) := (\boldsymbol{\alpha}, \boldsymbol{\Omega}_1, \mathcal{B}_1, \mathcal{S}_1), \tag{61}$$

in which $\boldsymbol{\alpha} = (\alpha_1, \ldots, \alpha_J)$ is the vector of PIPs α_j^{ss} (60), $\boldsymbol{\Omega}_1$ is the $J \times R$ matrix of posterior mixture weights $\omega_{1jk}^{\mathsf{ss}}$ (59), $\mathcal{B}_1$ is the set of posterior means $\boldsymbol{b}_{1jk}^{\mathsf{ss}}$ (57) for all j, k, and $\mathcal{S}_1$ is the set of posterior covariances $\boldsymbol{S}_{1jk}^{\mathsf{ss}}$ (58) for all j, k.

Updating a scaling factor in the prior. Here we extend the MSER model to allow for estimating a scaling parameter, $\sigma_0^2 \geqslant 0$, in which $\boldsymbol{S}_{0k} = \sigma_0^2 \boldsymbol{U}_k, k = 1, \ldots, K$. As above, we estimate σ_0^2 by maximum likelihood,

$$\hat{\sigma}_0^2 := \underset{\sigma_0^2}{\operatorname{argmax}}\, p(\boldsymbol{Y} \mid \boldsymbol{X}, \Theta) \tag{62}$$

which is equivalent to maximizing a weighted sum of the Bayes factors,

$$\hat{\sigma}_0^2 = \underset{\sigma_0^2}{\operatorname{argmax}} \sum_{j=1}^{J} \pi_j \mathrm{BF}^{\mathsf{mix}}(\boldsymbol{x}_j, \boldsymbol{Y}, \boldsymbol{V}, \mathcal{S}_0, \boldsymbol{\omega}). \tag{63}$$

As before, the maximum-likelihood estimate can be computed using a simple EM algorithm. The M-step update in the EM algorithm is given by

$$\sigma_0^2 = \sum_{j=1}^{J} \sum_{k=1}^{K} \frac{\alpha_j \omega_{1jk}}{R_k} \times \operatorname{tr}(\boldsymbol{U}_k^{\dagger} \mathbb{E}[\boldsymbol{b}\boldsymbol{b}^{\mathsf{T}} \mid \gamma_j = 1, \xi = k]), \tag{64}$$

in which $R_k \leqslant R$ denotes the rank of $\boldsymbol{U}_k$. The E-step in the EM algorithm consists of computing the posterior mixture weights ω_{1jk}, the posterior inclusion probabilities α_j, and the posterior second moments

$$\mathbb{E}[\boldsymbol{b}\boldsymbol{b}^{\mathsf{T}} \mid \gamma_j = 1, \xi = k] = \boldsymbol{b}_{1jk}\boldsymbol{b}_{1jk}^{\mathsf{T}} + \boldsymbol{S}_{1jk} \tag{65}$$

at the current setting of σ_0^2.

The mvSuSiE IBSS algorithm

The Iterative Bayesian Stepwise Selection (IBSS) algorithm for fitting mvSuSiE is derived by extending the ideas of [6] to the multivariate setting. Similar to IBSS for SuSiE, the IBSS algorithm for mvSuSiE is a coordinate ascent algorithm for optimizing a variational approximation [7, 8] to the posterior distribution for $\boldsymbol{B}^{(1)}, \ldots, \boldsymbol{B}^{(L)}$ under the mvSuSiE model. The basic mvSuSiE IBSS algorithm is given in Algorithm 1.

Lines 6–11 compute the posterior mean regression coefficients $\boldsymbol{b}^{(l)}$ for the lth single effect conditioned on each of the variables $j = 1, \ldots, J$ being the "single effect variable." These posteriors are then stored in a $J \times R$ matrix (lines 10–11). Once these are computed and stored, the unconditional posterior means $\bar{\boldsymbol{B}}^{(l)}$ are simply the conditional posterior means, $\boldsymbol{\mu}_l$, weighted by the PIPs $\boldsymbol{\alpha}_l$.

The optional step of estimating the scaling factors σ_{0l}^2 (line 5) is mainly for pruning unneeded single effects (see "Choice of L"). This parameter estimation step can be viewed as an EM update in which the E-step is approximate [9, 10].

Derivation of the mvSuSiE IBSS algorithm. The IBSS algorithm fits an approximate posterior distribution for $\boldsymbol{B}^{(1)}, \ldots, \boldsymbol{B}^{(L)}$ by minimizing a Kullback-Leibler (KL) divergence from the approximate posterior to the exact posterior subject to constraints on the approximate posterior. More precisely, denoting the exact posterior by p_{post} and the approximate posterior by q, we seek a q that minimizes the KL-divergence $D_{\mathsf{KL}}(q \,\|\, p_{\mathsf{post}})$ [11] subject to constraints on the q. Since the KL-divergence itself is hard to compute, we instead maximize the "evidence lower bound" (ELBO), which is equivalent to minimizing the KL-divergence, but easier to compute. The ELBO for the mvSuSiE model is

$$F(q, g, \boldsymbol{V}; \boldsymbol{X}, \boldsymbol{Y}) = \mathbb{E}_q[\log p(\boldsymbol{Y} \mid \boldsymbol{X}, \boldsymbol{B}^{(1)}, \ldots, \boldsymbol{B}^{(L)}, \boldsymbol{V})] + \mathbb{E}_q\left[\log\left\{\frac{p(\boldsymbol{B}^{(1)}, \ldots, \boldsymbol{B}^{(L)})}{q(\boldsymbol{B}^{(1)}, \ldots, \boldsymbol{B}^{(L)})}\right\}\right], \tag{66}$$

in which the prior $p(\boldsymbol{B}^{(1)}, \ldots, \boldsymbol{B}^{(L)}) = \prod_{l=1}^{L} p_l(\boldsymbol{B}^{(l)})$ is defined when thee mvSuSiE model is defined.

Similar to [6], we constrain the approximate posterior so that it factorizes by the single effects:

$$q(\boldsymbol{B}^{(1)}, \ldots, \boldsymbol{B}^{(L)}) = \prod_{l=1}^{L} q_l(\boldsymbol{B}^{(l)}). \tag{67}$$

Algorithm 1 Iterative Bayesian Stepwise Selection (IBSS) for mvSuSiE

Require: Data $\boldsymbol{X} \in \mathbb{R}^{N \times J}$, $\boldsymbol{Y} \in \mathbb{R}^{N \times R}$.
Require: Maximum number of non-zero effects, $L \in \{1, \ldots, J\}$.
Require: Initial estimates of the posterior mean single effects, $\bar{\boldsymbol{B}}^{(l)} \in \mathbb{R}^{J \times R}$, $l = 1, \ldots, L$.
Require: Initial estimates of the prior scaling factors, $\sigma_{01}^2, \ldots, \sigma_{0L}^2 \geqslant 0$.
Require: Residual covariance matrix $\boldsymbol{V}$ (must be invertible); prior inclusion probabilities $\boldsymbol{\pi} = (\pi_1, \ldots, \pi_J)$; prior mixture weights $\boldsymbol{\omega} = (\omega_1, \ldots, \omega_K)$; and prior covariance matrices $\mathcal{U} = (\boldsymbol{U}_1, \ldots, \boldsymbol{U}_K)$ (these do not need to be invertible).
Require: A function $\mathrm{MSER}(\boldsymbol{X}, \boldsymbol{Y}; \Theta) \rightarrow (\boldsymbol{\alpha}, \boldsymbol{\Omega}_1, \mathcal{B}_1, \mathcal{S}_1)$ that returns the posterior distribution of $\boldsymbol{b}, \boldsymbol{\gamma}$ under the MSER model with data $\boldsymbol{X}, \boldsymbol{Y}$ and parameters Θ; see (56).

repeat
  $\bar{\boldsymbol{R}} \leftarrow \boldsymbol{Y} - \boldsymbol{X} \sum_{l=1}^{L} \bar{\boldsymbol{B}}^{(l)}$ ▷ Compute expected residuals.
  for l in $1, \ldots, L$ **do**
    $\bar{\boldsymbol{R}}_l \leftarrow \bar{\boldsymbol{R}} + \boldsymbol{X} \bar{\boldsymbol{B}}^{(l)}$ ▷ Disregard lth single effect in residuals.
    Update σ_{0l}^2 ▷ Optional; see (64).
    $\Theta \leftarrow \{\boldsymbol{V}, \sigma_{0l}^2 \mathcal{U}, \boldsymbol{\omega}, \boldsymbol{\pi}\}$ ▷ Set MSER parameters.
    $(\boldsymbol{\alpha}, \boldsymbol{\Omega}_1, \mathcal{B}_1, \mathcal{S}_1) \leftarrow \mathrm{MSER}(\boldsymbol{X}, \bar{\boldsymbol{R}}_l; \Theta)$ ▷ Fit MSER to residuals.
    $\boldsymbol{\alpha}_l \leftarrow \boldsymbol{\alpha}$ ▷ Store PIPs for lth single effect.
    Initialize $\boldsymbol{\mu}_l$ to a $J \times R$ matrix of zeros ▷ Compute conditional posterior means (53).
    for j in $1, \ldots, J$ **do**
      Store $\sum_{k=1}^{K} \omega_{1jk} \boldsymbol{b}_{1jk}$ in row j of $\boldsymbol{\mu}_l$
    $\bar{\boldsymbol{B}}^{(l)} \leftarrow (\boldsymbol{\alpha}_l \boldsymbol{1}_R^{\intercal}) \circ \boldsymbol{\mu}_l$ ▷ "∘" denotes elementwise multiplication.
    $\bar{\boldsymbol{R}} \leftarrow \bar{\boldsymbol{R}}_l - \boldsymbol{X} \bar{\boldsymbol{B}}^{(l)}$ ▷ Update expected residuals.
until convergence criterion is satisfied
return $\boldsymbol{\alpha}_1, \ldots, \boldsymbol{\alpha}_L, \boldsymbol{\mu}_1, \ldots, \boldsymbol{\mu}_L$

Under this constraint, $\boldsymbol{B}^{(1)}, \ldots, \boldsymbol{B}^{(L)}$ are independent *a posteriori* annd each factor $q_l(\boldsymbol{B}^{(l)})$ is also a marginal posterior, and the right-hand part of the ELBO immediately simplifies:

$$F(q, g, \boldsymbol{V}; \boldsymbol{X}, \boldsymbol{Y}) = \mathbb{E}_q[\log p(\boldsymbol{Y} \mid \boldsymbol{X}, \boldsymbol{B}^{(1)}, \ldots, \boldsymbol{B}^{(L)}, \boldsymbol{V})] + \sum_{l=1}^{L} \mathbb{E}_q\left[\log \frac{p_l(\boldsymbol{B}^{(l)})}{q_l(\boldsymbol{B}^{(l)})}\right], \tag{68}$$

In the next section, we show that this simple conditional independence assumption also results in tractable computations, and in particular the computations that make up Algorithm 1. Note that no other approximations or constraints are needed beyond the conditional independence assumption (67).

Fitting the MSER to the residuals in Algorithm 1 maximizes the ELBO for a single effect. The main result of this section is summarized by the following proposition.

PROPOSITION 5. Let $F(q, g, \boldsymbol{V}; \boldsymbol{Y})$ be the ELBO (66) under the mvSuSiE model, and suppose that q is constrained to factorize over the single effects $l = 1, \ldots, L$ as in (67). The setting of $q_l(\boldsymbol{B}^{(l)})$ that maximizes the ELBO (66) while $g, \boldsymbol{V}$ and the other factors $q_{l'}(\boldsymbol{B}^{(l')})$, for all $l' \neq l$, are fixed has the following closed-form solution:

$$\begin{aligned} \hat{q}_l &:= \mathrm{argmax}_{q_l} F(q, g, \boldsymbol{V}; \boldsymbol{X}, \boldsymbol{Y}) \\ &= \mathrm{MSER}(\boldsymbol{X}, \bar{\boldsymbol{R}}_l; \Theta), \end{aligned} \tag{69}$$

in which $\mathrm{MSER}(\boldsymbol{X}, \boldsymbol{Y}; \Theta)$ defined in (56) gives the posterior distribution of $\boldsymbol{B}$ under the multivariate single effect regression (MSER) model given data $\boldsymbol{X}, \boldsymbol{Y}$ and model parameters Θ, and we define

$$\begin{aligned} \bar{\boldsymbol{R}}_l &:= \mathbb{E}_q[\boldsymbol{Y} - \boldsymbol{X} \textstyle\sum_{l' \neq l} \boldsymbol{B}^{(l')}] \\ &= \boldsymbol{Y} - \boldsymbol{X} \textstyle\sum_{l' \neq l} \bar{\boldsymbol{B}}^{(l')} \end{aligned} \tag{70}$$

as the $N \times R$ matrix of expected residuals that ignore the lth single effect, and $\bar{\boldsymbol{B}}^{(l)} := \mathbb{E}_q[\boldsymbol{B}^{(l)}]$.

COROLLARY 1. A corollary of Proposition 5 is that the optimal $q(\boldsymbol{B})$ factorizing as (67) is a product of factors $q_l(\boldsymbol{B}^{(l)})$ in which each factor is a posterior distribution under an MSER model (47).

This proposition and corollary are multivariate generalizations of the results for the univariate regression setting given in [6] (Propositions 1 and A1 in that paper). The proof of Proposition 5 is sketched out in the next two sections.

Special case when L = 1. The mvSuSiE model with $L = 1$ is an MSER model (47). For this special case, the ELBO (66) is

$$\begin{aligned} F_{\mathsf{MSER}}(q, g, \boldsymbol{V}; \boldsymbol{X}, \boldsymbol{Y}) &= \mathbb{E}_q[\log p(\boldsymbol{Y} \mid \boldsymbol{X}, \boldsymbol{B}^{(1)}, \boldsymbol{V})] - D_{\mathsf{KL}}(q \,\|\, p) \\ &= -\tfrac{N}{2} \log |2\pi \boldsymbol{V}| - \tfrac{1}{2} \mathrm{tr}(\boldsymbol{V}^{-1} \mathrm{ERSS}) - D_{\mathsf{KL}}(q(\boldsymbol{B}^{(1)}) \,\|\, p(\boldsymbol{B}^{(1)})), \end{aligned} \tag{71}$$

in which “ERSS” is the multivariate expected residual sum of squares,

$$\mathrm{ERSS} := \mathbb{E}_q[(\boldsymbol{Y} - \boldsymbol{X}\boldsymbol{B}^{(1)})^\intercal (\boldsymbol{Y} - \boldsymbol{X}\boldsymbol{B}^{(1)})]. \tag{72}$$

In these expressions, we have dropped the “l” subscripts whenever they appear since there is only one single effect l. (We have kept the “(l)” superscript for $\boldsymbol{B}$ to avoid confusing $\boldsymbol{B}^{(1)}$ with $\boldsymbol{B}$ defined in the prior.)

The variational distribution maximizing the ELBO (71), $\hat{q} := \mathrm{argmax}_q\, F_{\mathsf{MSER}}(q, g, \boldsymbol{V}; \boldsymbol{X}, \boldsymbol{Y})$, is the true posterior, $\hat{q}(\boldsymbol{B}^{(1)}) = p_{\mathsf{post}}(\boldsymbol{B}^{(1)}) := p(\boldsymbol{B}^{(1)} \mid \boldsymbol{X}, \boldsymbol{Y}, \boldsymbol{V}, g)$ [7]; that is, q is the posterior distribution under an MSER model. At $\hat{q}$, the ELBO (71) is equal to the marginal log-likelihood; that is, $F_{\mathsf{MSER}}(\hat{q}, g, \boldsymbol{V}; \boldsymbol{X}, \boldsymbol{Y}) = \log p(\boldsymbol{Y} \mid \boldsymbol{X}, \boldsymbol{V}, g)$.

Coordinate ascent update for lth single effect. To allow for posterior computations that are tractable, we restricted q to the set of posterior distributions that factorize over the single effects $l = 1, \ldots, L$ (67). With this approximation, we can now divide and conquer; we consider the problem of finding a $q_l(\boldsymbol{B}^{(l)})$ that maximizes the ELBO (66) while the remaining factors are fixed.

Expanding terms involving q_l, the ELBO (66) is

$$F(q, g, \boldsymbol{V}; \boldsymbol{X}, \boldsymbol{Y}) = -\tfrac{N}{2} \log |2\pi \boldsymbol{V}| - \tfrac{1}{2} \mathrm{tr}(\boldsymbol{V}^{-1} \mathrm{ERSS}) - D_{\mathsf{KL}}(q_l(\boldsymbol{B}^{(l)}) \,\|\, g_l(\boldsymbol{B}^{(l)})) + \mathrm{const}, \tag{73}$$

where the “const” is a placeholder for terms in the ELBO not involving q_l, and the exprected residual sum of squares (ERSS) in this expression is

$$\mathrm{ERSS} := \mathbb{E}_q[(\boldsymbol{Y} - \boldsymbol{X}\boldsymbol{B})^\intercal (\boldsymbol{Y} - \boldsymbol{X}\boldsymbol{B})]. \tag{74}$$

As a reminder, $\boldsymbol{B} = \sum_{l=1}^{L} \boldsymbol{B}^{(l)}$. Expanding the ERSS further, and making use of the property from (67) that the covariances are zero between all $\boldsymbol{B}^{(l)}$ and $\boldsymbol{B}^{(l')}$ whenever $l \neq l'$, the ELBO can be rewritten as

$$F(q, g, \boldsymbol{V}; \boldsymbol{X}, \boldsymbol{Y}) = -\tfrac{N}{2} \log |2\pi \boldsymbol{V}| - \tfrac{1}{2} \mathrm{tr}[\boldsymbol{V}^{-1} \mathrm{ERSS}(l)] - D_{\mathsf{KL}}(q_l(\boldsymbol{B}^{(l)}) \,\|\, g_l(\boldsymbol{B}^{(l)})) + \mathrm{const}, \tag{75}$$

in which

$$\mathrm{ERSS}(l) := \mathbb{E}_q[(\bar{\boldsymbol{R}}_l - \boldsymbol{X}\boldsymbol{B}^{(l)})^\intercal (\bar{\boldsymbol{R}}_l - \boldsymbol{X}\boldsymbol{B}^{(l)})], \tag{76}$$

and $\bar{\boldsymbol{R}}_l$ is defined in (70). Assuming $l = 1$ without loss of generality, (75) is of the exact same form as (71) after ignoring terms that do not involve q_l, and after replacing $\boldsymbol{Y}$ with $\bar{\boldsymbol{R}}_l$.

In summary, the ELBO for mvSuSiE with $L > 1$ can be rearranged to exactly match the expression for the mvSuSiE ELBO with $L = 1$ if we ignore terms not involving q_l.

Computing the ELBO

While computing the ELBO (68) is not strictly needed to implement the IBSS algorithm, in practice it is useful for monitoring progress of the IBSS algorithm and for comparing different mvSuSiE model fits. Here we explain how we compute the ELBO.

Starting from (74), the "ERSS" part of the ELBO is

$$\begin{aligned}\mathrm{ERSS} &:= \mathbb{E}_q[(\boldsymbol{Y}-\boldsymbol{X}\boldsymbol{B})^\intercal(\boldsymbol{Y}-\boldsymbol{X}\boldsymbol{B})] \\ &= (\boldsymbol{Y}-\boldsymbol{X}\bar{\boldsymbol{B}})^\intercal(\boldsymbol{Y}-\boldsymbol{X}\bar{\boldsymbol{B}}) - \textstyle\sum_{l=1}^L (\bar{\boldsymbol{B}}^{(l)})^\intercal \boldsymbol{X}^\intercal\boldsymbol{X}\bar{\boldsymbol{B}}^{(l)} + \mathbb{E}_q[(\boldsymbol{B}^{(l)})^\intercal\boldsymbol{X}^\intercal\boldsymbol{X}\boldsymbol{B}^{(l)}]. \end{aligned} \tag{77}$$

From the definition of the mvSuSiE model, for a given single effect l only one row of $\boldsymbol{B}^{(l)}$ contains nonzero values, so $\mathbb{E}_q[b_{jr}b_{kr'}] = b_{jr}b_{kr'} = 0$ for any $j \neq k$ and $r, r' \in \{1,\ldots,R\}$, where b_{jr} is an entry of $\boldsymbol{B}^{(l)}$. Therefore, the ERSS simplifies to

$$\mathrm{ERSS} = (\boldsymbol{Y}-\boldsymbol{X}\bar{\boldsymbol{B}})^\intercal(\boldsymbol{Y}-\boldsymbol{X}\bar{\boldsymbol{B}}) - \sum_{l=1}^L (\bar{\boldsymbol{B}}^{(l)})^\intercal\boldsymbol{X}^\intercal\boldsymbol{X}\bar{\boldsymbol{B}}^{(l)} + \sum_{l=1}^L (\bar{\boldsymbol{B}}^{(l)})^\intercal\boldsymbol{D}\bar{\boldsymbol{B}}^{(l)} + \sum_{l=1}^L\sum_{j=1}^J d_{jj}\boldsymbol{C}_j^{(l)}, \tag{78}$$

where $\boldsymbol{D}$ is a $J \times J$ diagonal matrix with diagonal entries $d_{jj} := (\boldsymbol{X}^\intercal\boldsymbol{X})_{jj}$ and $\boldsymbol{C}_j^{(l)}$ is the $R \times R$ covariance matrix for the jth row of $\boldsymbol{B}^{(l)}$ with respect to the approximate posterior q_l, which can be asily computed from the results of Proposition 4.

The remaining terms in the ELBO (68) are KL-divergences,

$$D_{\mathsf{KL}}(q_l \,\|\, p_l) = -\mathbb{E}_q\left[\log\frac{p_l(\boldsymbol{B}^{(l)})}{q_l(\boldsymbol{B}^{(l)})}\right]. \tag{79}$$

It is most convenient to compute this when $q_l(\boldsymbol{B}^{(l)})$ is updated, as we show next.

Computing the KL-divergence when L = 1. From (71), the KL-divergence for the mvSuSiE model with $L = 1$ is

$$D_{\mathsf{KL}}(q \,\|\, p) = -\tfrac{N}{2}\log|2\pi\boldsymbol{V}| - \tfrac{1}{2}\mathrm{tr}(\boldsymbol{V}^{-1}\mathrm{ERSS}) - F_{\mathsf{MSER}}(q, g, \boldsymbol{V}; \boldsymbol{X}, \boldsymbol{Y}), \tag{80}$$

where the ERSS is given in (72). Recall, the optimal variational distribution is equal to the true posterior, $\hat{q}(\boldsymbol{B}^{(1)}) = p(\boldsymbol{B}^{(1)} \mid \boldsymbol{X}, \boldsymbol{Y}, \boldsymbol{V}, g)$. And when the optimal variational distribution is attained, the ELBO is equal to the marginal log-likelihood. Therefore, we have

$$\begin{aligned} D_{\mathsf{KL}}(\hat{q} \,\|\, p) &= -\tfrac{N}{2}\log|2\pi\boldsymbol{V}| - \tfrac{1}{2}\mathrm{tr}(\boldsymbol{V}^{-1}\mathrm{ERSS}) - \log p(\boldsymbol{Y} \mid \boldsymbol{X}, \boldsymbol{V}, g) \\ &= -\tfrac{N}{2}\log|2\pi\boldsymbol{V}| - \tfrac{1}{2}\mathrm{tr}(\boldsymbol{V}^{-1}\mathrm{ERSS}) - \log\textstyle\sum_{j=1}^J \mathrm{BF}^{\mathsf{mix}}(\boldsymbol{x}_j, \boldsymbol{Y}, \boldsymbol{V}, \sigma_0^2\mathcal{U}, \boldsymbol{\omega}) \\ &\quad - \log p(\boldsymbol{Y} \mid \boldsymbol{X}, \boldsymbol{B} = \boldsymbol{0}, \boldsymbol{V}) \\ &= -\tfrac{1}{2}\mathrm{tr}[\boldsymbol{V}^{-1}(\mathrm{ERSS} - \boldsymbol{Y}^\intercal\boldsymbol{Y})] - \log\textstyle\sum_{j=1}^J \mathrm{BF}^{\mathsf{mix}}(\boldsymbol{x}_j, \boldsymbol{Y}, \boldsymbol{V}, \sigma_0^2\mathcal{U}, \boldsymbol{\omega}), \end{aligned} \tag{81}$$

in which the Bayes factor $\mathrm{BF}^{\mathsf{mix}}$ was derived in Proposition 4. Finally, to arrive at the desired KL-divergence $D_{\mathsf{KL}}(\hat{q}_l \,\|\, p_l)$, we substitute $\bar{\boldsymbol{R}}_l$ for $\boldsymbol{Y}$ in (81). A similar approach to computing the ELBO was taken in [6].

The IBSS algorithm for mvSuSiE with sufficient statistics

Algorithm 2 outlines the IBSS algorithm for mvSuSiE with sufficient statistics in which the computations are rearranged so that they only require the sufficient statistics $\boldsymbol{X}^\intercal\boldsymbol{Y}$ and $\boldsymbol{X}^\intercal\boldsymbol{X}$.

Proofs and extended derivations

Proof of Proposition 2. First we give the proof from the maximum-likelihood estimation perspective in which we treat $\boldsymbol{\mu}.$ as a free parameter to be optimized.

Algorithm 2 IBSS for mvSuSiE with sufficient statistics (IBSS-ss)

Require: Data $\boldsymbol{X}^\intercal \boldsymbol{Y} \in \mathbb{R}^{J \times R}, \boldsymbol{X}^\intercal \boldsymbol{X} \in \mathbb{R}^{J \times J}$.
Require: Maximum number of non-zero effects, $L \in \{1, \ldots, J\}$.
Require: Initial estimates of the posterior mean single effects, $\bar{\boldsymbol{B}}^{(l)} \in \mathbb{R}^{J \times R}$, $l = 1, \ldots, L$.
Require: Initial estimates of the prior scaling factors, $\sigma_{01}^2, \ldots, \sigma_{0L}^2 \geqslant 0$.
Require: Residual covariance matrix $\boldsymbol{V}$ (must be invertible); prior inclusion probabilities $\boldsymbol{\pi} = (\pi_1, \ldots, \pi_J)$; prior mixture weights $\boldsymbol{\omega} = (\omega_1, \ldots, \omega_K)$; and prior covariance matrices $\mathcal{U} = (\boldsymbol{U}_1, \ldots, \boldsymbol{U}_K)$ (these do not need to be invertible).
Require: A function MSER-ss$(\boldsymbol{X}^\intercal \boldsymbol{X}, \boldsymbol{X}^\intercal \boldsymbol{Y}; \Theta) \rightarrow (\boldsymbol{\alpha}, \boldsymbol{\omega}_1, \mathcal{B}_1, \mathcal{S}_1)$ that returns the posterior distribution of $\boldsymbol{b}, \boldsymbol{\gamma}$ under the MSER model with data $\boldsymbol{X}^\intercal \boldsymbol{X}, \boldsymbol{X}^\intercal \boldsymbol{Y}$ and parameters Θ.

repeat
  $\bar{\boldsymbol{P}} \leftarrow \boldsymbol{X}^\intercal \boldsymbol{Y} - \boldsymbol{X}^\intercal \boldsymbol{X} \sum_{l=1}^{L} \bar{\boldsymbol{B}}^{(l)}$ ▻ Compute expected residuals.
  for l in $1, \ldots, L$ **do**
    $\bar{\boldsymbol{P}}_l \leftarrow \bar{\boldsymbol{P}} + \boldsymbol{X}^\intercal \boldsymbol{X} \bar{\boldsymbol{B}}^{(l)}$ ▻ Disregard lth single effect in residuals.
    Update σ_{0l}^2 ▻ Optional; see (64).
    $\Theta \leftarrow \{\boldsymbol{V}, \sigma_{0l}^2 \mathcal{U}, \boldsymbol{\omega}, \boldsymbol{\pi}\}$ ▻ Set MSER parameters.
    $(\boldsymbol{\alpha}, \boldsymbol{\Omega}_1, \mathcal{B}_1, \mathcal{S}_1) \leftarrow$ MSER-ss$(\boldsymbol{X}^\intercal \boldsymbol{X}, \bar{\boldsymbol{P}}_l; \Theta)$ ▻ Fit MSER to residuals.
    $\boldsymbol{\alpha}_l \leftarrow \boldsymbol{\alpha}$ ▻ Store PIPs for lth single effect.
    Initialize $\boldsymbol{\mu}_l$ to a $J \times R$ matrix of zeros ▻ Compute conditional posterior means (53).
    for j in $1, \ldots, J$ **do**
      Store $\sum_{k=1}^{K} \omega_{1jk} \boldsymbol{b}_{1jk}$ in row j of $\boldsymbol{\mu}_l$
    $\bar{\boldsymbol{B}}^{(l)} \leftarrow (\boldsymbol{\alpha}_l \boldsymbol{1}_R^\intercal) \circ \boldsymbol{\mu}_l$ ▻ "∘" denotes elementwise multiplication.
    $\bar{\boldsymbol{P}} \leftarrow \bar{\boldsymbol{P}}_l - \boldsymbol{X}^\intercal \boldsymbol{X} \bar{\boldsymbol{B}}^{(l)}$ ▻ Update expected residuals.
until convergence criterion is satisfied
return $\boldsymbol{\alpha}_1, \ldots, \boldsymbol{\alpha}_L, \boldsymbol{\mu}_1, \ldots, \boldsymbol{\mu}_L$

The log-likelihood for $\boldsymbol{\mu}$ and $\boldsymbol{b}$ is

$$\log \ell(\boldsymbol{\mu}, \boldsymbol{b}; \boldsymbol{x}, \boldsymbol{Y}, \boldsymbol{V}) = -\tfrac{1}{2} \mathrm{tr}[\boldsymbol{V}^{-1} (\boldsymbol{Y} - \boldsymbol{1}\boldsymbol{\mu}^\intercal - \boldsymbol{x}\boldsymbol{b}^\intercal)^\intercal (\boldsymbol{Y} - \boldsymbol{1}\boldsymbol{\mu}^\intercal - \boldsymbol{x}\boldsymbol{b}^\intercal)] + \text{const},$$

in which the "const" includes additional terms that do not involve $\boldsymbol{\mu}$ or $\boldsymbol{b}$. Differentiating with respect to $\boldsymbol{\mu}$, setting the partial derivatives to zero, and solving for $\boldsymbol{\mu}$ yields

$$\boldsymbol{V}^{-1} \boldsymbol{\mu} = \boldsymbol{V}^{-1} (\bar{\boldsymbol{y}} - \bar{x} \boldsymbol{b}),$$

and so

$$\hat{\boldsymbol{\mu}} = \bar{\boldsymbol{y}} - \bar{x} \boldsymbol{b}.$$

The profile likelihood for $\boldsymbol{b}$ is therefore

$$\begin{aligned} \ell^*(\boldsymbol{b}; \boldsymbol{x}, \boldsymbol{Y}, \boldsymbol{V}) &:= \max_{\boldsymbol{\mu}} \ell(\boldsymbol{\mu}, \boldsymbol{b}; \boldsymbol{x}, \boldsymbol{Y}, \boldsymbol{V}) \\ &= |2\pi \boldsymbol{V}|^{-n/2} \exp\{-\tfrac{1}{2} \mathrm{tr}[\boldsymbol{V}^{-1} (\boldsymbol{Y} - \boldsymbol{1}\hat{\boldsymbol{\mu}}^\intercal - \boldsymbol{x}\boldsymbol{b}^\intercal)^\intercal (\boldsymbol{Y} - \boldsymbol{1}\hat{\boldsymbol{\mu}}^\intercal - \boldsymbol{x}\boldsymbol{b}^\intercal)]\} \\ &= |2\pi \boldsymbol{V}|^{-n/2} \exp\{-\tfrac{1}{2} \mathrm{tr}[\boldsymbol{V}^{-1} ((\boldsymbol{Y} - \boldsymbol{1}\bar{\boldsymbol{y}}^\intercal) - (\boldsymbol{x} - \bar{x}\boldsymbol{1})\boldsymbol{b}^\intercal)^\intercal ((\boldsymbol{Y} - \boldsymbol{1}\bar{\boldsymbol{y}}^\intercal) - (\boldsymbol{x} - \bar{x}\boldsymbol{1})\boldsymbol{b}^\intercal)]\} \\ &= \ell(\boldsymbol{b}; \tilde{\boldsymbol{x}}, \tilde{\boldsymbol{Y}}, \boldsymbol{V}). \end{aligned}$$

The conditional posterior for $\boldsymbol{\mu}$ given $\boldsymbol{b}$ is

$$p(\boldsymbol{\mu} \mid \boldsymbol{x}, \boldsymbol{Y}, \boldsymbol{V}, \boldsymbol{S}_{0\mu}, \boldsymbol{b}) \propto \exp\{-\tfrac{1}{2} \boldsymbol{\mu}_1 \boldsymbol{S}_{1\mu}^{-1} \boldsymbol{\mu}_1\},$$

which is the multivariate normal density with mean

$$\boldsymbol{\mu}_1 := N(\boldsymbol{S}_{0\mu}^{-1} + N\boldsymbol{V}^{-1})^{-1} \boldsymbol{V}^{-1} (\bar{\boldsymbol{y}} - \bar{x}\boldsymbol{b}) = \boldsymbol{S}_{1\mu} \hat{\boldsymbol{S}}^{-1} \hat{\boldsymbol{\mu}}$$

and covariance

$$\boldsymbol{S}_{1\mu} := (\boldsymbol{S}_{0\mu}^{-1} + N\boldsymbol{V}^{-1})^{-1} = (\boldsymbol{S}_{0\mu}^{-1} + \hat{\boldsymbol{S}}^{-1})^{-1}.$$

The marginal likelihood for $\boldsymbol{b}$ obtained by integrating over $\boldsymbol{\mu}$ is

$$\begin{aligned}\ell^\star(\boldsymbol{b};\boldsymbol{x},\boldsymbol{Y},\boldsymbol{V},\boldsymbol{S}_{0a}) &= \textstyle\int \ell(\boldsymbol{\mu},\boldsymbol{b};\boldsymbol{x},\boldsymbol{Y},\boldsymbol{V})\,p(\boldsymbol{\mu}\mid\boldsymbol{S}_{0\mu})\,d\boldsymbol{\mu} \\ &= \textstyle\int |2\pi\boldsymbol{S}_{0\mu}|^{-1/2}|2\pi\boldsymbol{V}|^{-N/2} \\ &\qquad\times\exp\{-\tfrac{1}{2}\mathrm{tr}[\boldsymbol{V}^{-1}(\boldsymbol{Y}-\boldsymbol{x}\boldsymbol{b}^\intercal-\boldsymbol{1}\boldsymbol{\mu}^\intercal)^\intercal(\boldsymbol{Y}-\boldsymbol{x}\boldsymbol{b}^\intercal-\boldsymbol{1}\boldsymbol{\mu}^\intercal)+\boldsymbol{S}_{0\mu}^{-1}\boldsymbol{\mu}\boldsymbol{\mu}^\intercal]\}\,d\boldsymbol{\mu}.\end{aligned}$$

Expanding terms, we get

$$\begin{aligned}&\ell^\star(\boldsymbol{b};\boldsymbol{x},\boldsymbol{Y},\boldsymbol{V},\boldsymbol{S}_{0\mu}) \\ &= |2\pi\boldsymbol{S}_{0\mu}|^{-1/2}|2\pi\boldsymbol{V}|^{-N/2}\textstyle\int\exp\{-\tfrac{1}{2}\mathrm{tr}[\boldsymbol{S}_{1\mu}^{-1}\boldsymbol{\mu}\boldsymbol{\mu}^\intercal-2\boldsymbol{V}^{-1}(\boldsymbol{Y}-\boldsymbol{x}\boldsymbol{b}^\intercal)^\intercal\boldsymbol{1}\boldsymbol{\mu}^\intercal \\ &\qquad\qquad+\boldsymbol{V}^{-1}(\boldsymbol{Y}-\boldsymbol{x}\boldsymbol{b}^\intercal)^\intercal(\boldsymbol{Y}-\boldsymbol{x}\boldsymbol{b}^\intercal)]\}\,d\boldsymbol{\mu} \\ &= |2\pi\boldsymbol{S}_{0\mu}|^{-1/2}|2\pi\boldsymbol{V}|^{-N/2}\textstyle\int\exp\{-\tfrac{1}{2}\mathrm{tr}[\boldsymbol{S}_{1\mu}^{-1}(\boldsymbol{\mu}-\boldsymbol{\mu}_1)(\boldsymbol{\mu}-\boldsymbol{\mu}_1)^\intercal \\ &\qquad\qquad-N\boldsymbol{V}^{-1}(\bar{\boldsymbol{y}}-\bar{x}\boldsymbol{b})(\bar{\boldsymbol{y}}-\bar{x}\boldsymbol{b})^\intercal N\boldsymbol{V}^{-1}\boldsymbol{S}_{1\mu} \\ &\qquad\qquad+\boldsymbol{V}^{-1}(\boldsymbol{Y}-\boldsymbol{x}\boldsymbol{b}^\intercal)^\intercal(\boldsymbol{Y}-\boldsymbol{x}\boldsymbol{b}^\intercal)]\}\,d\boldsymbol{\mu} \\ &= |\boldsymbol{S}_{0\mu}|^{-1/2}|2\pi\boldsymbol{V}|^{-N/2}|\boldsymbol{S}_{1\mu}|^{1/2}\exp\{-\tfrac{1}{2}\mathrm{tr}[\boldsymbol{V}^{-1}(\boldsymbol{Y}-\boldsymbol{x}\boldsymbol{b}^\intercal)^\intercal(\boldsymbol{Y}-\boldsymbol{x}\boldsymbol{b}^\intercal) \\ &\qquad\qquad-N\boldsymbol{V}^{-1}(\bar{\boldsymbol{y}}-\bar{x}\boldsymbol{b})(\bar{\boldsymbol{y}}-\bar{x}\boldsymbol{b})^\intercal N\boldsymbol{V}^{-1}\boldsymbol{S}_{1\mu}]\} \\ &= |2\pi\boldsymbol{V}|^{-N/2}|\boldsymbol{S}_{0\mu}^{-1}\boldsymbol{S}_{1\mu}|^{1/2}\exp\{\tfrac{1}{2}\boldsymbol{\mu}_1^\intercal\boldsymbol{S}_{1\mu}^{-1}\boldsymbol{\mu}_1-\tfrac{1}{2}\mathrm{tr}[\boldsymbol{V}^{-1}(\boldsymbol{Y}-\boldsymbol{x}\boldsymbol{b}^\intercal)^\intercal(\boldsymbol{Y}-\boldsymbol{x}\boldsymbol{b}^\intercal)]\}.\end{aligned}$$

When $\boldsymbol{S}_{0\mu}^{-1}\to\boldsymbol{0}$, the marginal likelihood simplifies to

$$\begin{aligned}&\ell^\star(\boldsymbol{b};\boldsymbol{x},\boldsymbol{Y},\boldsymbol{V},\boldsymbol{S}_{0\mu}) \\ &= |2\pi\boldsymbol{V}|^{-N/2}|\boldsymbol{S}_{0\mu}^{-1}\hat{\boldsymbol{S}}_\mu|^{1/2}\exp\{-\tfrac{1}{2}\mathrm{tr}\left[\boldsymbol{V}^{-1}(\boldsymbol{Y}-\boldsymbol{x}\boldsymbol{b}^\intercal)^\intercal(\boldsymbol{Y}-\boldsymbol{x}\boldsymbol{b}^\intercal)-N\boldsymbol{V}^{-1}(\bar{\boldsymbol{y}}-\bar{x}\boldsymbol{b})(\bar{\boldsymbol{y}}-\bar{x}\boldsymbol{b})^\intercal\right]\} \\ &= |2\pi\boldsymbol{V}|^{-N/2}|\boldsymbol{S}_{0\mu}^{-1}\hat{\boldsymbol{S}}_\mu|^{1/2}\exp\{-\tfrac{1}{2}\mathrm{tr}\left[\boldsymbol{V}^{-1}(\boldsymbol{Y}-\boldsymbol{1}\bar{\boldsymbol{y}}^\intercal-(\boldsymbol{x}-\bar{x}\boldsymbol{1})\boldsymbol{b}^\intercal)^\intercal(\boldsymbol{Y}-\boldsymbol{1}\bar{\boldsymbol{y}}^\intercal-(\boldsymbol{x}-\bar{x}\boldsymbol{1})\boldsymbol{b}^\intercal)\right]\} \\ &= |\boldsymbol{S}_{0\mu}^{-1}\hat{\boldsymbol{S}}_\mu|^{1/2}\times\ell(\boldsymbol{b};\tilde{\boldsymbol{x}},\tilde{\boldsymbol{Y}},\boldsymbol{V}).\end{aligned}$$

The marginal likelihood for $\boldsymbol{b}$ in the model with an intercept (and $\boldsymbol{x},\boldsymbol{Y}$ are not centered) is therefore proportional to the likelihood for the model without an intercept and when $\boldsymbol{x},\boldsymbol{Y}$ are centered.

References

[1] Zou, Y., P. Carbonetto, G. Wang, and M. Stephens (2022). Fine-mapping from summary data with the "Sum of Single Effects" model. *PLoS Genetics 18*(7), e1010299.

[2] Dawid, A. P. (1981). Some matrix-variate distribution theory: notational considerations and a Bayesian application. *Biometrika 68*(1), 265–274.

[3] Gupta, A. and D. Nagar (2000). *Matrix variate distributions.* Boca Raton, FL: Chapman & Hall.

[4] Dempster, A. P., N. M. Laird, and D. B. Rubin (1977). Maximum likelihood from incomplete data via the EM algorithm. *Journal of the Royal Statistical Society, Series B 39*(1), 1–22.

[5] Chipman, H., E. I. George, and R. E. McCulloch (2001). The practical implementation of Bayesian model selection. In P. Lahiri (Ed.), *Model Selection*, Volume 38 of *IMS Lecture Notes*, pp. 65–116. Beachwood, OH: Institute of Mathematical Statistics.

[6] Wang, G., A. Sarkar, P. Carbonetto, and M. Stephens (2020). A simple new approach to variable selection in regression, with application to genetic fine mapping. *Journal of the Royal Statistical Society, Series B 82*(5), 1273–1300.

[7] Blei, D. M., A. Kucukelbir, and J. D. McAuliffe (2017). Variational inference: a review for statisticians. *Journal of the American Statistical Association 112*(518), 859–877.

[8] Jordan, M. I., Z. Ghahramani, T. S. Jaakkola, and L. K. Saul (1999). An introduction to variational methods for graphical models. *Machine Learning 37*(2), 183–233.

[9] Heskes, T., O. Zoeter, and W. Wiegerinck (2004). Approximate expectation maximization. In S. Thrun, L. K. Saul, and B. Schölkopf (Eds.), *Advances in Neural Information Processing Systems 16*, pp. 353–360. MIT Press.

[10] Neal, R. M. and G. E. Hinton (1998). A view of the EM algorithm that justifies incremental, sparse, and other variants. In M. I. Jordan (Ed.), *Learning in Graphical Models*, pp. 355–368. New York, NY: Springer.

[11] Cover, T. M. and J. A. Thomas (2006). *Elements of Information Theory* (2nd ed.). Wiley-Interscience.

[12] Schmidt, E. M., J. Zhang, W. Zhou, J. Chen, K. L. Mohlke, Y. E. Chen, and C. J. Willer (2015). GREGOR: evaluating global enrichment of trait-associated variants in epigenomic features using a systematic, data-driven approach. *Bioinformatics 31*(16), 2601–2606.

[13] Finucane, H. K., B. Bulik-Sullivan, A. Gusev, G. Trynka, Y. Reshef, P. R. Loh, V. Anttila, H. Xu, C. Zang, K. Farh, S. Ripke, F. R. Day, S. Purcell, E. Stahl, S. Lindstrom, J. R. Perry, Y. Okada, S. Raychaudhuri, M. J. Daly, N. Patterson, B. M. Neale, and A. L. Price (2015). Partitioning heritability by functional annotation using genome-wide association summary statistics. *Nature Genetics 47*(11), 1228–1235.

[14] Gusev, A., S. H. Lee, G. Trynka, H. Finucane, B. J. Vilhjálmsson, H. Xu, C. Zang, S. Ripke, B. Bulik-Sullivan, E. Stahl, A. K. Kähler, C. M. Hultman, S. M. Purcell, S. A. McCarroll, M. Daly, B. Pasaniuc, P. F. Sullivan, B. M. Neale, N. R. Wray, S. Raychaudhuri, and A. L. Price (2014). Partitioning heritability of regulatory and cell-type-specific cariants across 11 common diseases. *American Journal of Human Genetics 95*(5), 535–552.

[15] Vasquez, Y. M., E. C. Mazur, X. Li, R. Kommagani, L. Jiang, R. Chen, R. B. Lanz, E. Kovanci, W. E. Gibbons, and F. J. DeMayo (2015). FOXO1 is required for binding of PR on IRF4, novel transcriptional regulator of endometrial stromal decidualization. *Molecular Endocrinology 29*(3), 421–433.

[16] Hormozdiari, F., S. Gazal, B. van de Geijn, H. K. Finucane, C. J. Ju, P.-R. Loh, A. Schoech, Y. Reshef, X. Liu, L. O'Connor, A. Gusev, E. Eskin, and A. L. Price (2018). Leveraging molecular quantitative trait loci to understand the genetic architecture of diseases and complex traits. *Nature Genetics 50*(7), 1041–1047.

[17] Ulirsch, J. C., C. A. Lareau, E. L. Bao, L. S. Ludwig, M. H. Guo, C. Benner, A. T. Satpathy, V. K. Kartha, R. M. Salem, J. N. Hirschhorn, H. K. Finucane, M. J. Aryee, J. D. Buenrostro, and V. G. Sankaran (2019). Interrogation of human hematopoiesis at single-cell and single-variant resolution. *Nature Genetics 51*(4), 683–693.

[18] Vuckovic, D., E. L. Bao, P. Akbari, C. A. Lareau, A. Mousas, et al. (2020). The polygenic and monogenic basis of blood traits and diseases. *Cell 182*(5), 1214–1231.e11.

[19] Stephens, M. (2017). False discovery rates: a new deal. *Biostatistics 18*(2), 275–294.

Supplementary Figures

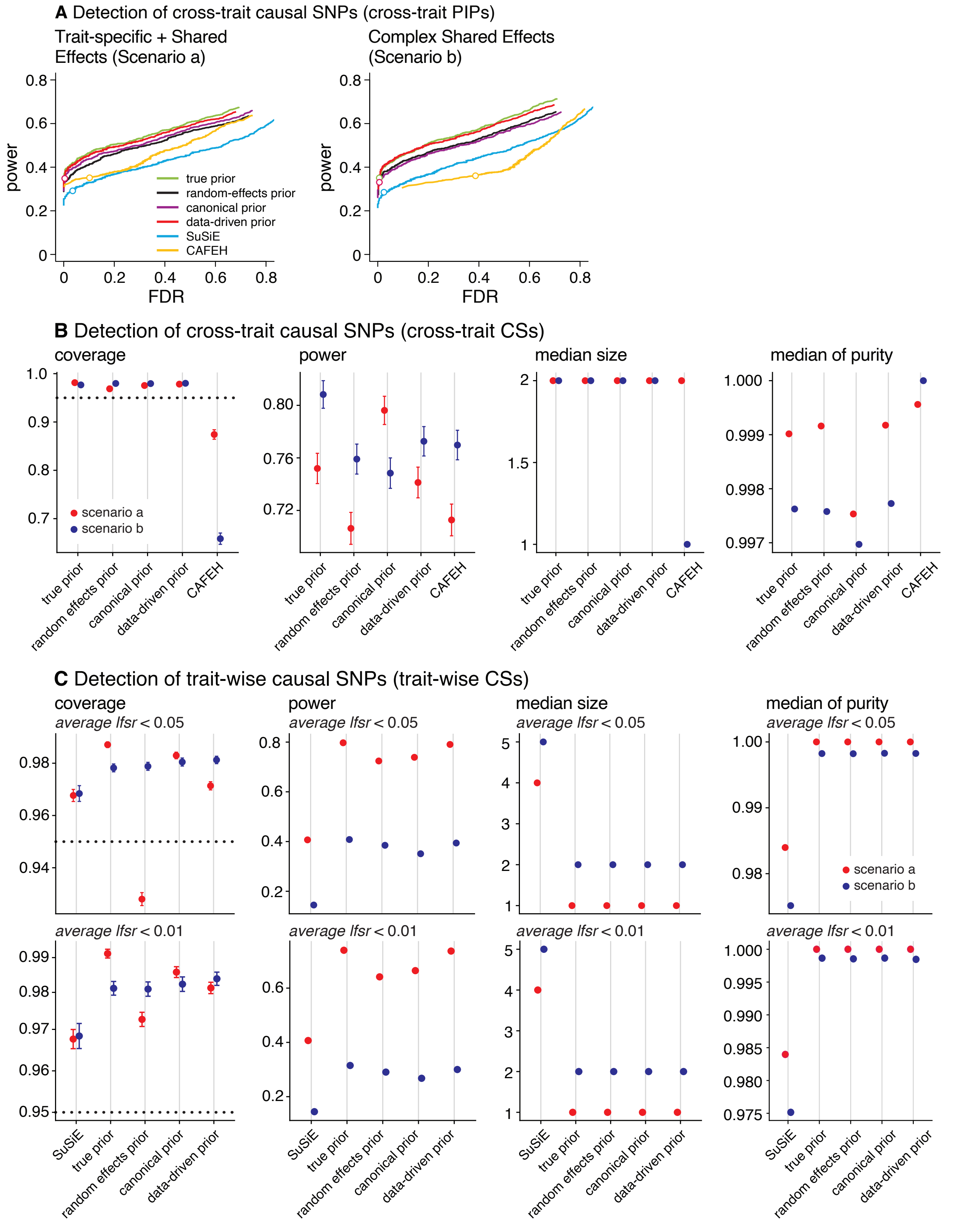

Supplementary Figure 1. Comparison of mvSuSiE variants with different priors. See the next page for details.

Supplementary Figure 1 (previous page). Comparison of mvSuSiE variants with different priors. This figure is a companion to Fig. 2 giving more detail about the performance of mvSuSiE with different priors. In addition to SuSiE and CAFEH, four variants of mvSuSiE are compared: mvSuSiE with random effects prior; canonical prior; data-driven prior; and mvSuSiE with the true prior, that is the prior used to simulate the true effects. See the Fig. 2 caption for additional explanations of the plots.

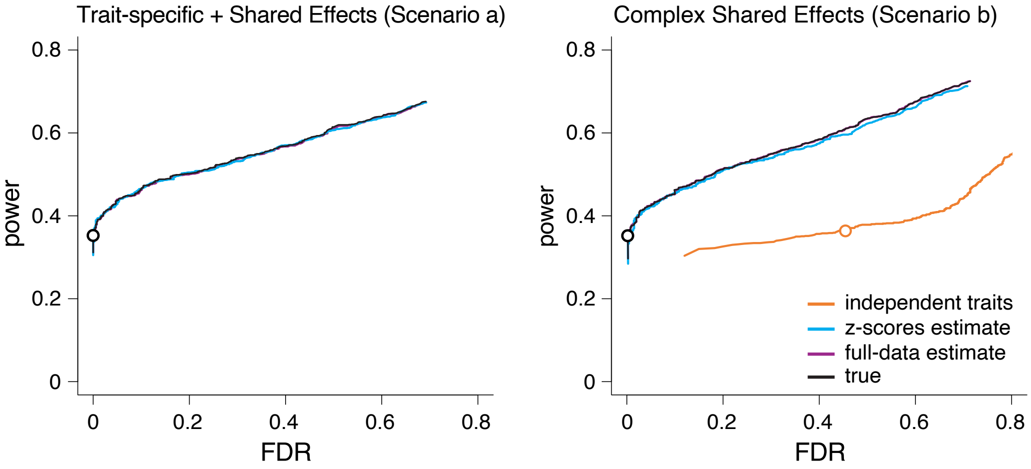

Supplementary Figure 2. Comparison of mvSuSiE variants with different residual covariances. The following choices of $\boldsymbol{V}$ are compared: identity matrix ("independent traits"); $\boldsymbol{V}$ estimated from the individual-level data ("full-data estimate"); $\boldsymbol{V}$ estimated from the *z*-scores ("*z*-scores estimate"); the $\boldsymbol{V}$ used to simulate the data ("true"). Note that in Scenario a, the true residual covariance matrix was the identity matrix, so the results are exactly the same for both "true" and "independent traits." FDR and power were calculated as the cross-trait PIP was varied from 0 to 1 (n = 600 simulations). Open circles are drawn at a PIP threshold of 0.95. For these comparisons only, the prior was set to the mixture distribution used to simulate the effects. In Scenario a, in which the traits were simulated with $\boldsymbol{V} = \boldsymbol{I}_R$, there was almost no difference in assuming or not assuming $\boldsymbol{V} = \boldsymbol{I}_R$ in the mvSuSiE analysis; all the power-FDR curves closely overlap in the plot. In the right-hand plot (Scenario b), $\boldsymbol{Y}$ was simulated with $\boldsymbol{V} \neq \boldsymbol{I}_R$, and in that case there was a substantial reduction in performance when $\boldsymbol{V} = \boldsymbol{I}_R$ was assumed in the mvSuSiE analysis ("independent traits").

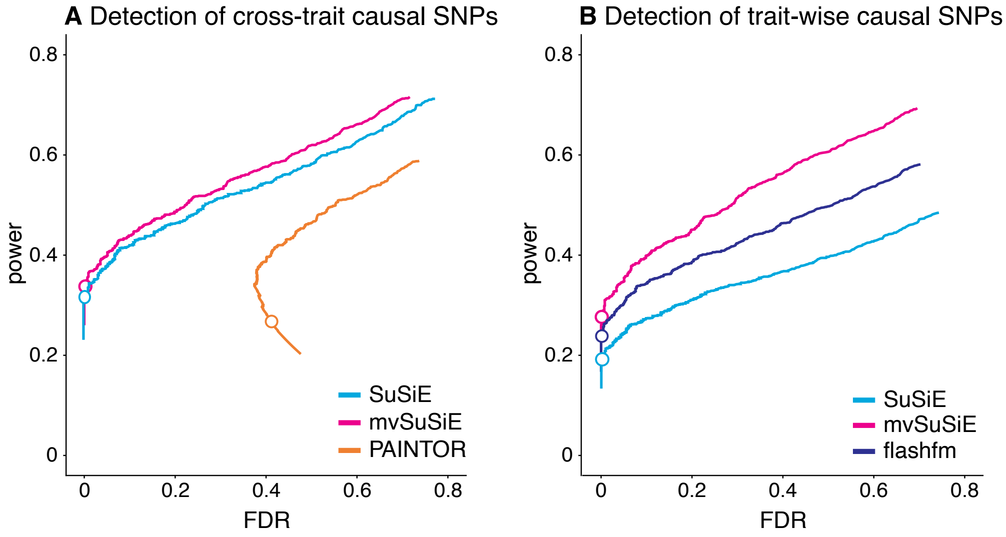

Supplementary Figure 3. Comparison of fine-mapping methods in simulations with two independent traits and independent effects. Panel A shows power vs. FDR in identifying cross-trait causal SNPs using PIPs (or max-PIP for SuSiE). In A, FDR and power were calculated in the 2-trait scenario as the threshold was varied from 0 to 1 (n = 600 simulations). Open circles are drawn at a threshold of 0.95. Note that flashfm does not provide a cross-trait measure so it is not included in A. Panel B shows power vs. FDR in identifying trait-wise causal SNPs. In B, FDR and power are calculated from the 600 simulations as the *marginal posterior probability (MPP)* threshold (flashfm), *PIP* (SuSiE), or *minimum lfsr* (mvSuSiE) is varied from 0 to 1. Open circles are drawn at a *minimum lfsr* threshold of 0.05 or a PIP/MPP threshold of 0.95. Note that PAINTOR does not provide a trait-wise measure so was not included in B.

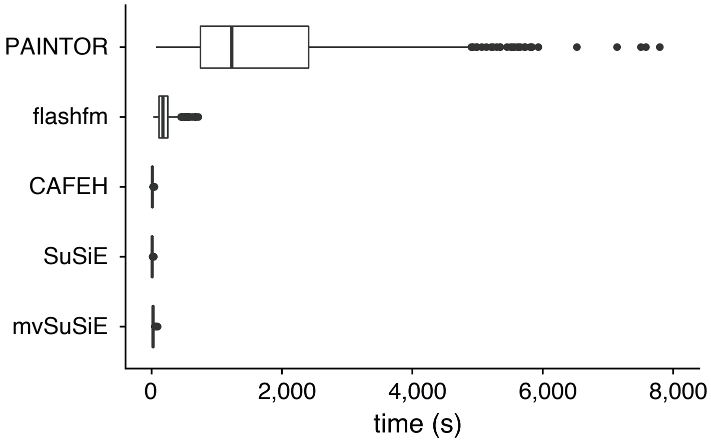

Supplementary Figure 4. Analysis runtimes in simulations with two independent traits and independent effects. The box plots summarize the analysis runtimes for PAINTOR, flashfm, CAFEH, SuSiE and mvSuSiE in the 2-trait simulations (n = 600 simulations). The box plot whiskers depict 1.5× the interquartile range, the box bounds represent the upper and lower quartiles (25th and 75th percentiles), the center line represents the median (50th percentile), and points represent outliers. The flashfm runtimes include FINEMAP computations. Note that a single run of PAINTOR taking longer than 8 h (30,000 s) is not shown in the plot.

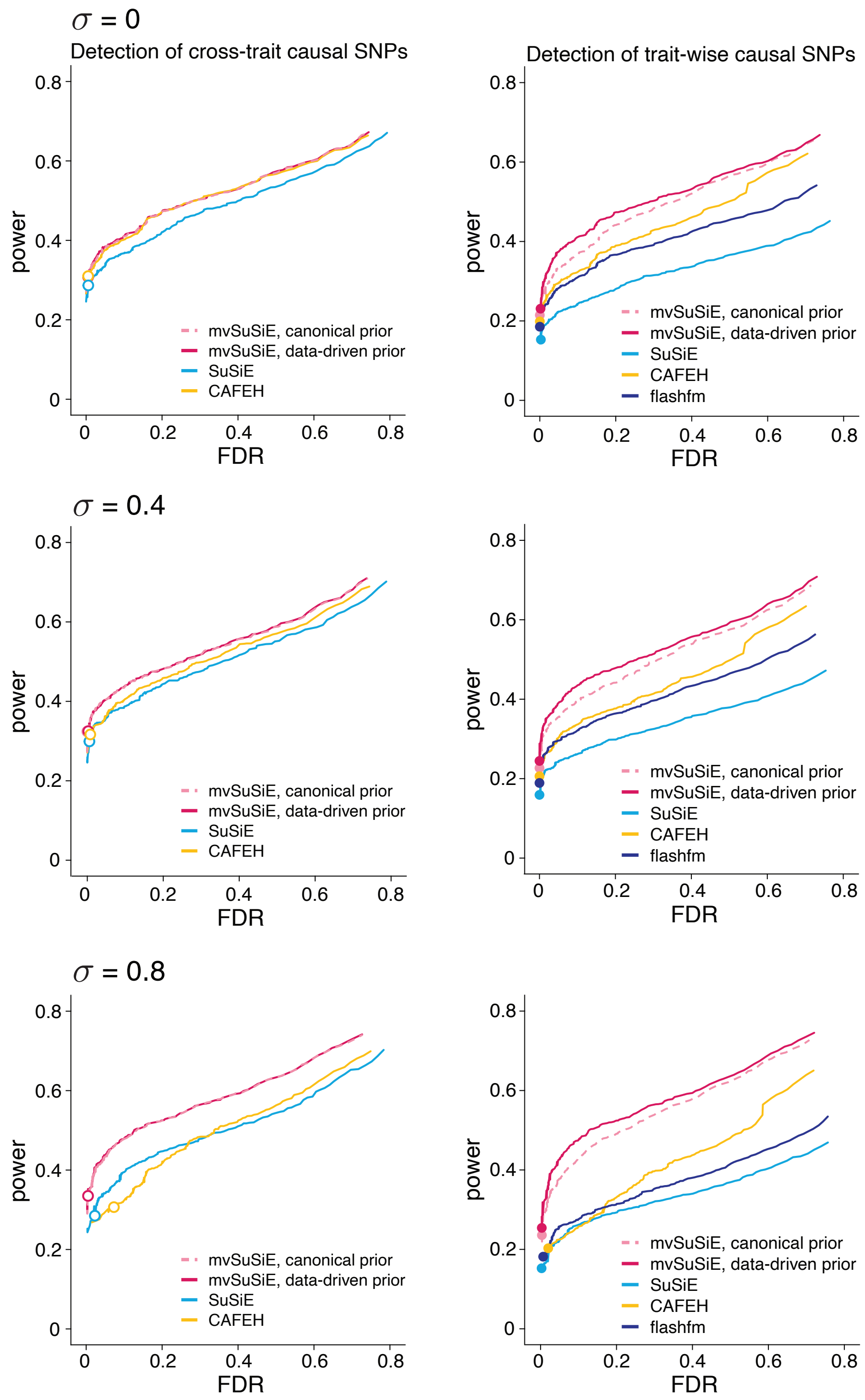

Supplementary Figure 5. Comparison of fine-mapping methods in simulations with two correlated traits and independent effects, Part A: detection of cross-trait and trait-wise causal SNPs using SNP-wise measures. In these simulations, two traits were simulated with correlated residuals, with correlation σ = 0, 0.4, 0.8. For each setting of σ, 600 data sets were simulated. The plots on the left-hand side show power vs. FDR in identifying cross-trait causal SNPs using PIPs (or max-PIP for SuSiE). FDR and power were calculated in as the threshold was varied from 0 to 1 (n = 600 simulations). Open circles are drawn at a threshold of 0.95. Note that flashfm does not provide a cross-trait measure so it was not included in the left-hand plots. The plots on the right-hand side show power vs. FDR in identifying trait-wise causal SNPs. FDR and power were calculated from the 600 simulations as the *marginal posterior probability (MPP)* threshold (flashfm), *PIP* (SuSiE), or *minimum lfsr* (mvSuSiE) is varied from 0 to 1. Closed circles are drawn at a *minimum lfsr* threshold of 0.01 or a PIP/MPP threshold of 0.99. Also note that the results shown here for σ = 0 are the same as the top row of Supplementary Fig. 8 and (for some methods) Supplementary Fig. 3.

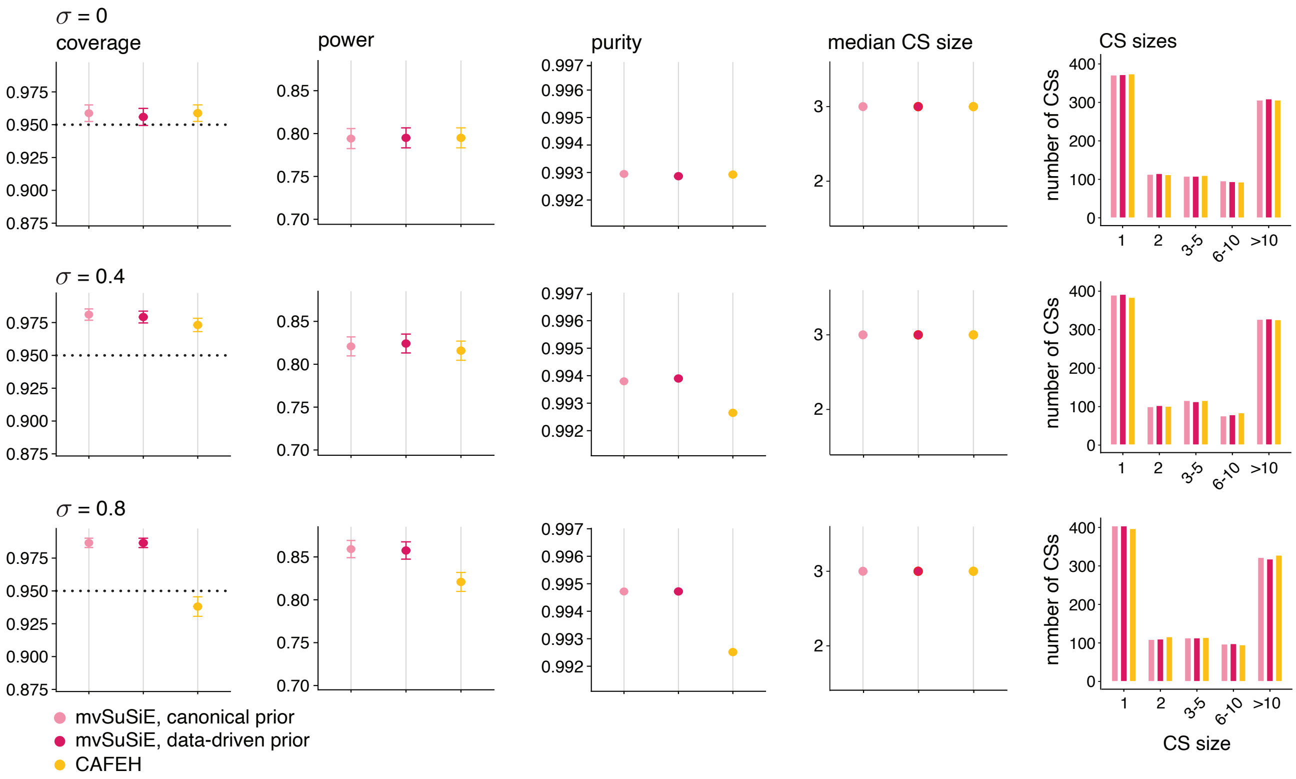

Supplementary Figure 6. Comparison of fine-mapping methods in simulations with two correlated traits and independent effects, Part B: detection of cross-trait causal SNPs (cross-trait CSs). The dotted horizontal lines show the target coverage (95%). Error bars show 2 times the empirical s.e. from the results across the n = 600 simulations. Note that flashfm does not provide cross-trait CSs and therefore was not included in these plots. See Supplementary Fig. 5 for Part A of these results, and for more details. Also note that the plots shown here for $\sigma = 0$ are the same as the plots in the top row of Supplementary Fig. 9.

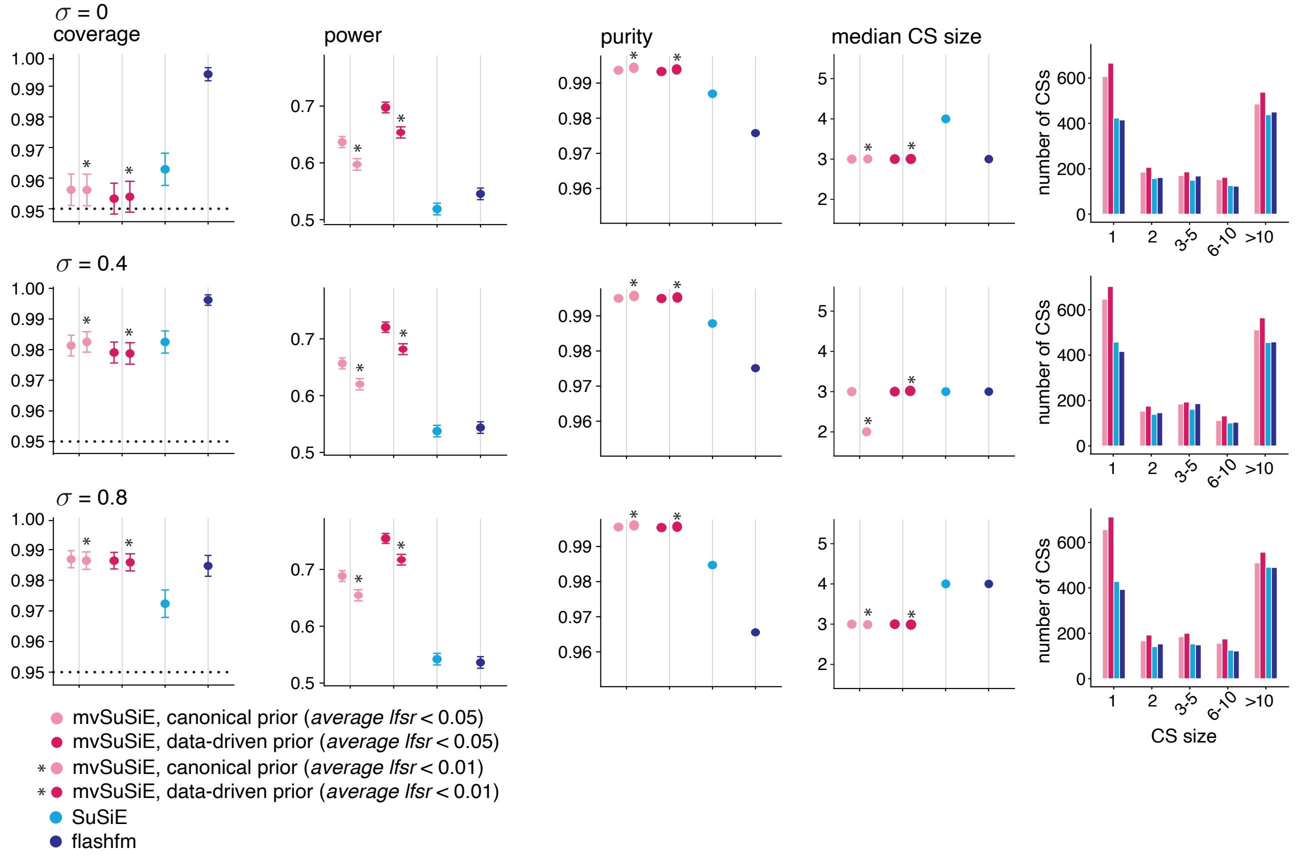

Supplementary Figure 7. Comparison of fine-mapping methods in simulations with two correlated traits and independent effects, Part C: detection of trait-wise causal SNPs (trait-wise significant CSs). See Supplementary Fig. 5 for Part A of these results, and for more details. CAFEH does not provide trait-wise CSs so was not included in these plots. Also note that the plots shown here for $\sigma = 0$ are the same as the plots in the top row of Supplementary Fig. 10.

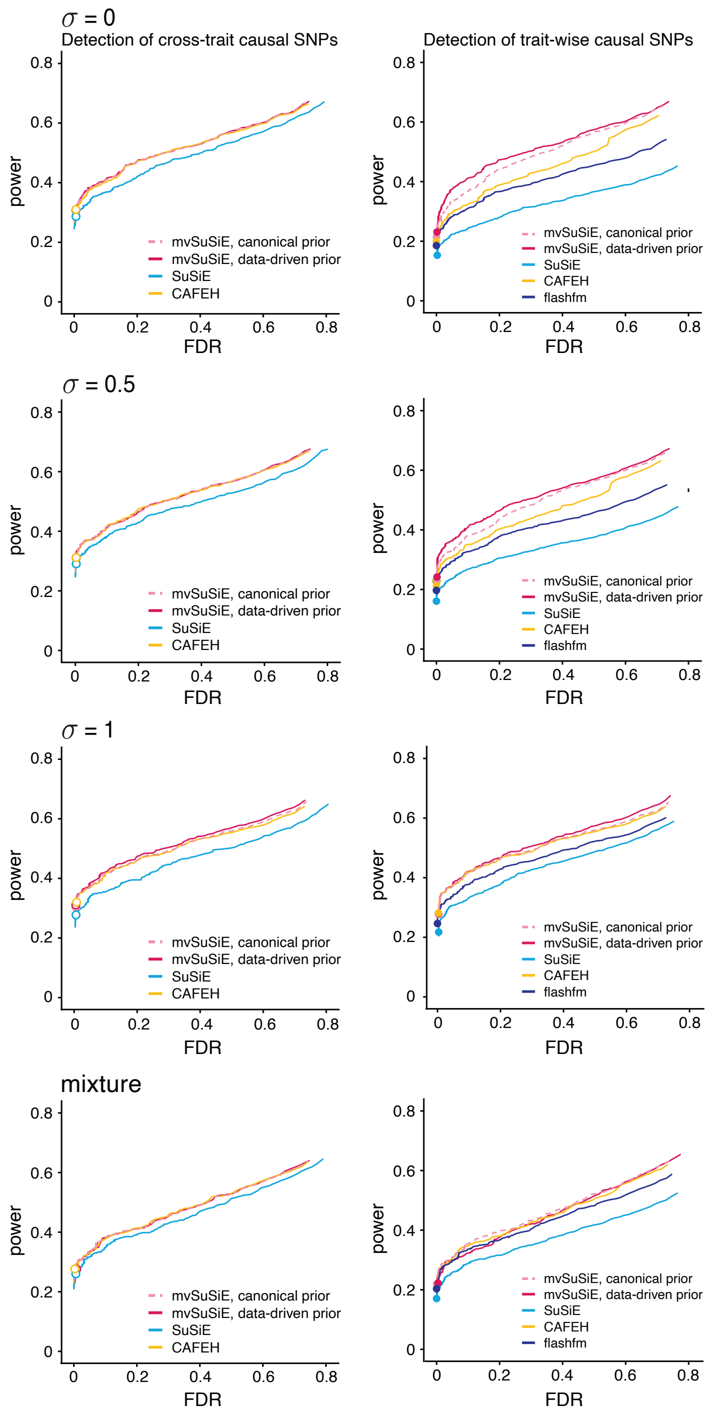

Supplementary Figure 8. Comparison of fine-mapping methods in simulations with two independent traits and correlated effects, Part A: detection of cross-trait and trait-wise causal SNPs using SNP-wise measures. In these simulations, two independent traits were simulated with correlated effects, with correlation σ = 0, 0.5, 1. 600 data sets were simulated for each choice of σ. In a fourth set of simulations (bottom row), the effects were simulated from a mixture of multivariate normals with different covariances (see "Simulation Scenarios" in the Online Methods for details). The plots on the left-hand side show power vs. FDR in identifying cross-trait causal SNPs using PIPs (or max-PIP for SuSiE). FDR and power were calculated as the threshold was varied from 0 to 1 (n = 600 simulations). Open circles are drawn at a threshold of 0.95. Note that flashfm does not provide a cross-trait measure so it is not included in the left-hand plots. The plots on the right-hand side show power vs. FDR in identifying trait-wise causal SNPs. FDR and power were calculated from the 600 simulations as the *marginal posterior probability (MPP)* threshold (flashfm), *PIP* (SuSiE), or *minimum lfsr* (mvSuSiE) was varied from 0 to 1. Closed circles are drawn at a *minimum lfsr* threshold of 0.01 or a PIP/MPP threshold of 0.99. Also note that the results shown here for σ = 0 are the same as the top row of Supplementary Fig. 5 and (for some methods) Supplementary Fig. 3.

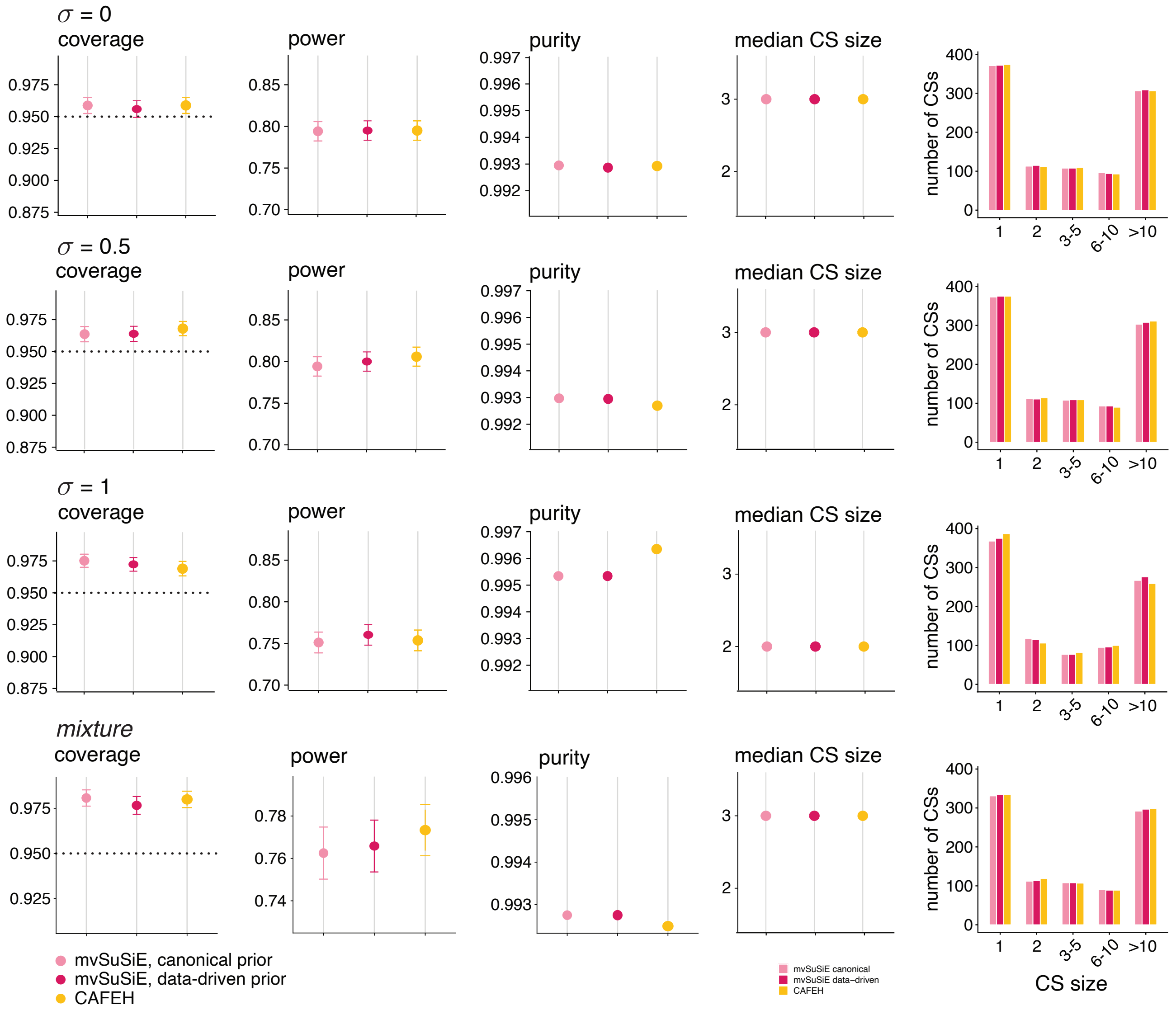

Supplementary Figure 9. Comparison of fine-mapping methods in simulations with two independent traits and correlated effects, Part B: detection of cross-trait causal SNPs (cross-trait CSs). See Supplementary Fig. 8 for Part A of these results, and for further explanations. The dotted horizontal lines show the target coverage (95%) and error bars show 2 times the empirical s.e. from the results in the $n = 600$ simulations. Note that flashfm does not provide cross-trait CSs and therefore was not included in these plots. Also note that the plots in the top row are the same as the $\sigma = 0$ plots in Supplementary Fig. 6.

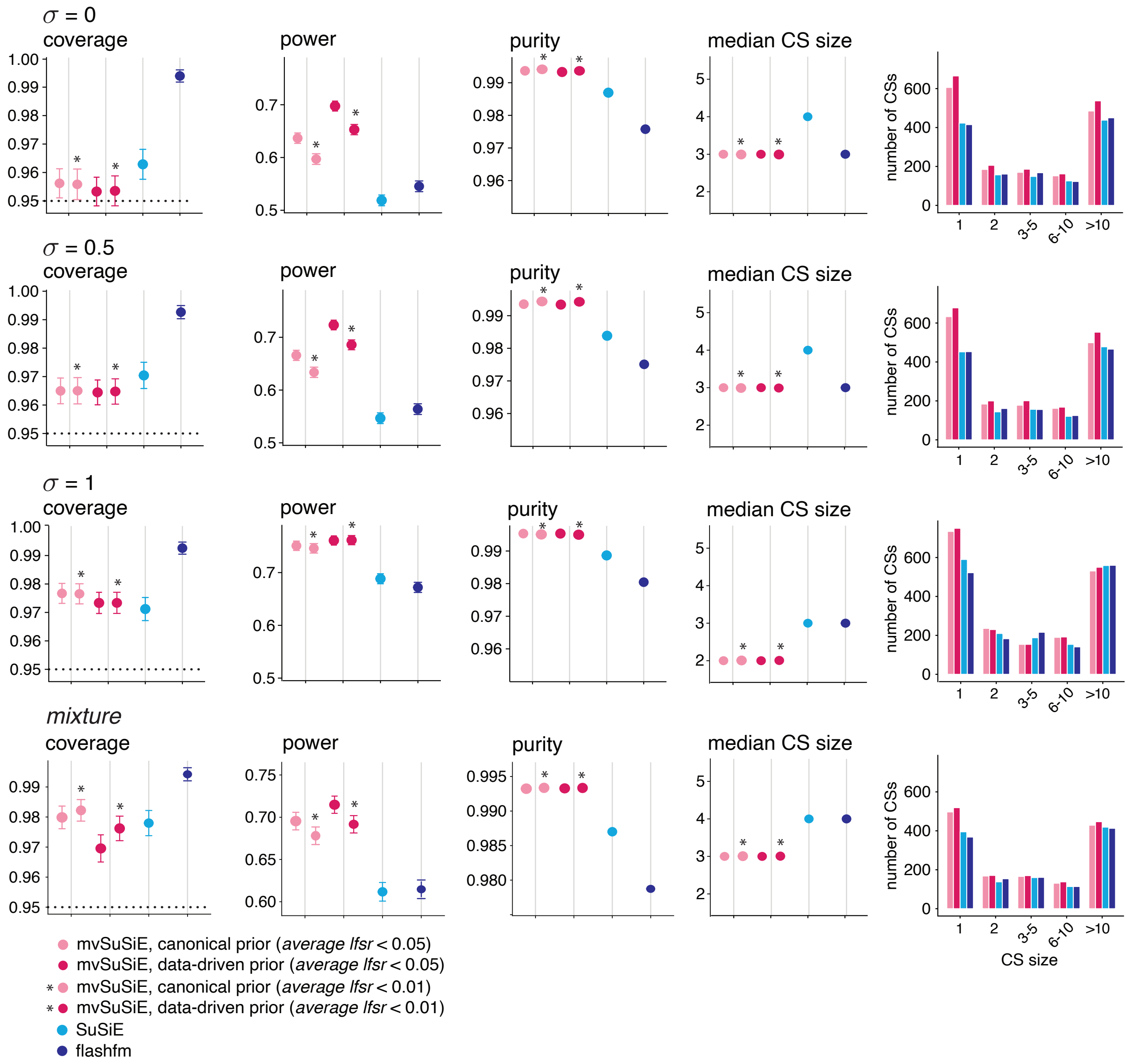

Supplementary Figure 10. Comparison of fine-mapping methods in simulations with two independent traits and correlated effects, Part C: detection of trait-wise causal SNPs (trait-wise significant CSs). See Supplementary Fig. 8 for Part A of these results, and for further explanations. Note that CAFEH does not provide trait-wise CSs so was not included in in these plots. Also note that the plots in the top row are the same as the $\sigma = 0$ plots in Supplementary Fig. 7.

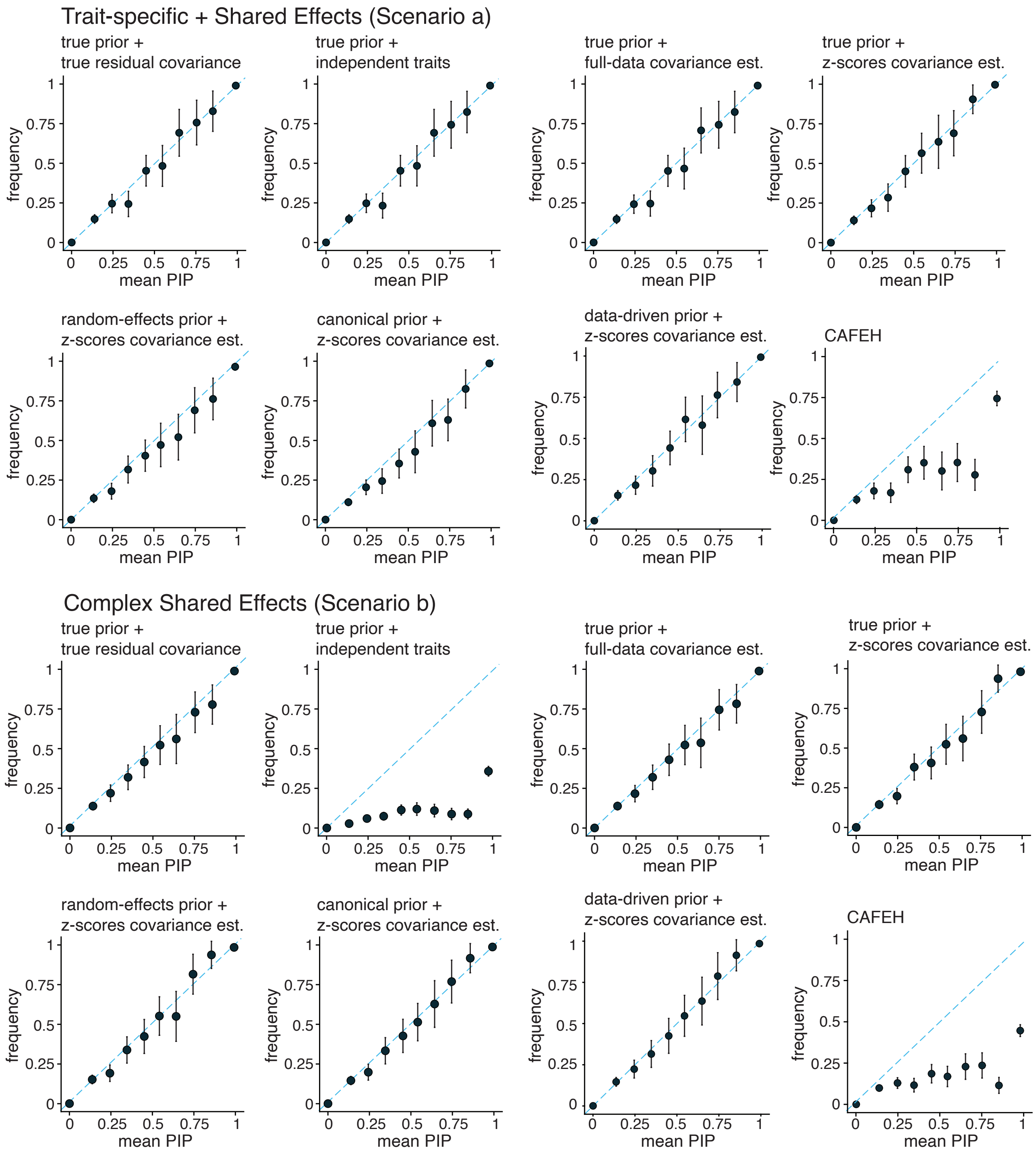

Supplementary Figure 11. Assessment of mvSuSiE and CAFEH PIP calibration. In each scenario, SNPs from all simulations (n = 600) were grouped into bins according to their reported PIP (10 equally spaced bins from 0 to 1).The plots show the average PIP from each bin (X axis) against the proportion of SNPs in that bin that are causal (Y axis). For a given bin, the error bar depicts 2 times the empirical s.e. from all n = 600 simulations. A well-calibratedmethod should produce points near the diagonal. See Supplementary Figures 1 and 2 for details on the mvSuSiE variants compared.

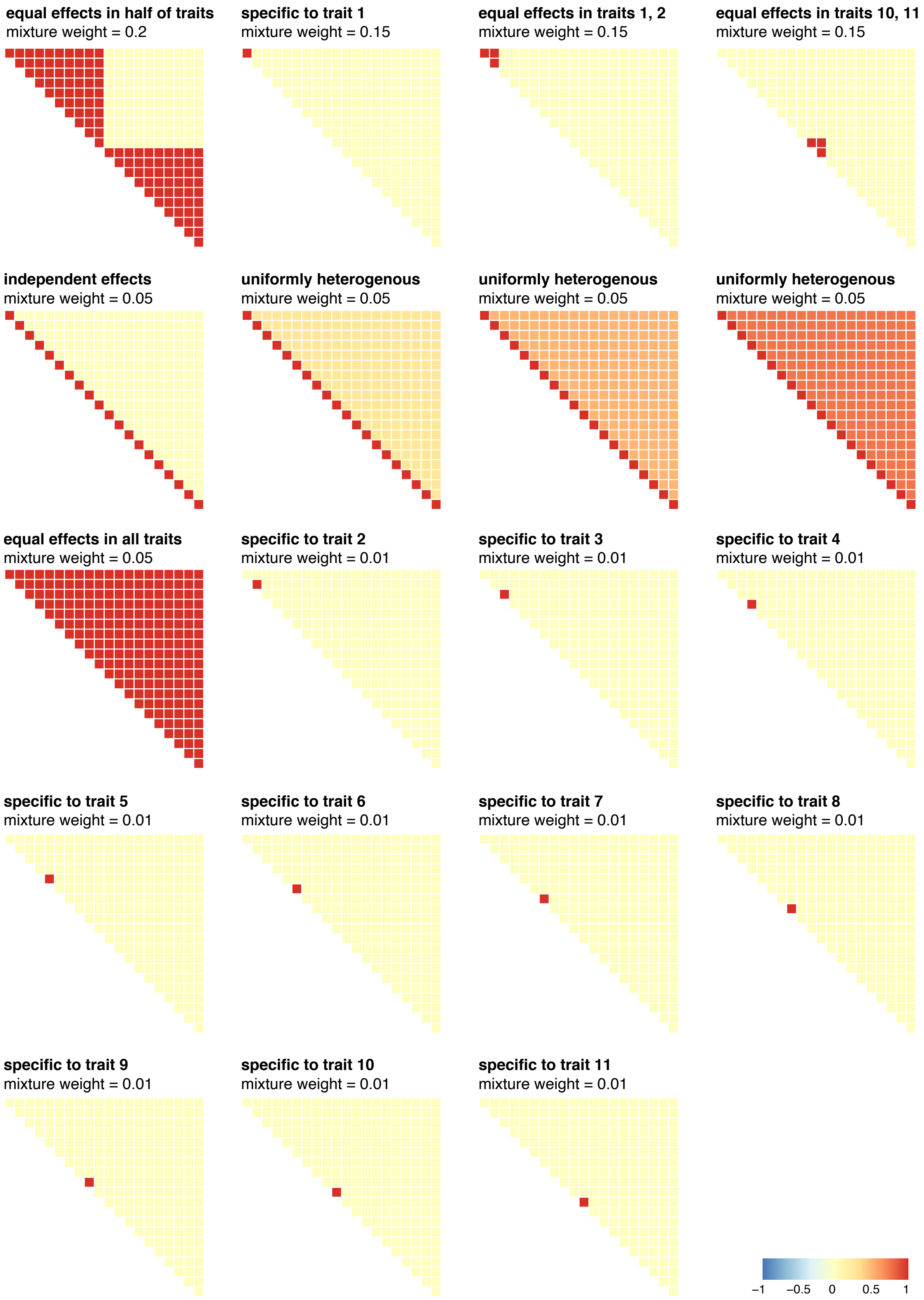

Supplementary Figure 12. Covariance matrices used to simulate the effects of the causal SNPs in Scenario a. Each plot shows a 20 × 20 covariance matrix, $\boldsymbol{U}_k$, and its corresponding mixture weight, ω_k, in the mixture-of-multivariate normals distribution used to simulate the effects of the causal SNPs. Note all covariance matrices contain elements spanning the range 0 to 1, and none of these matrices contain negative elements.

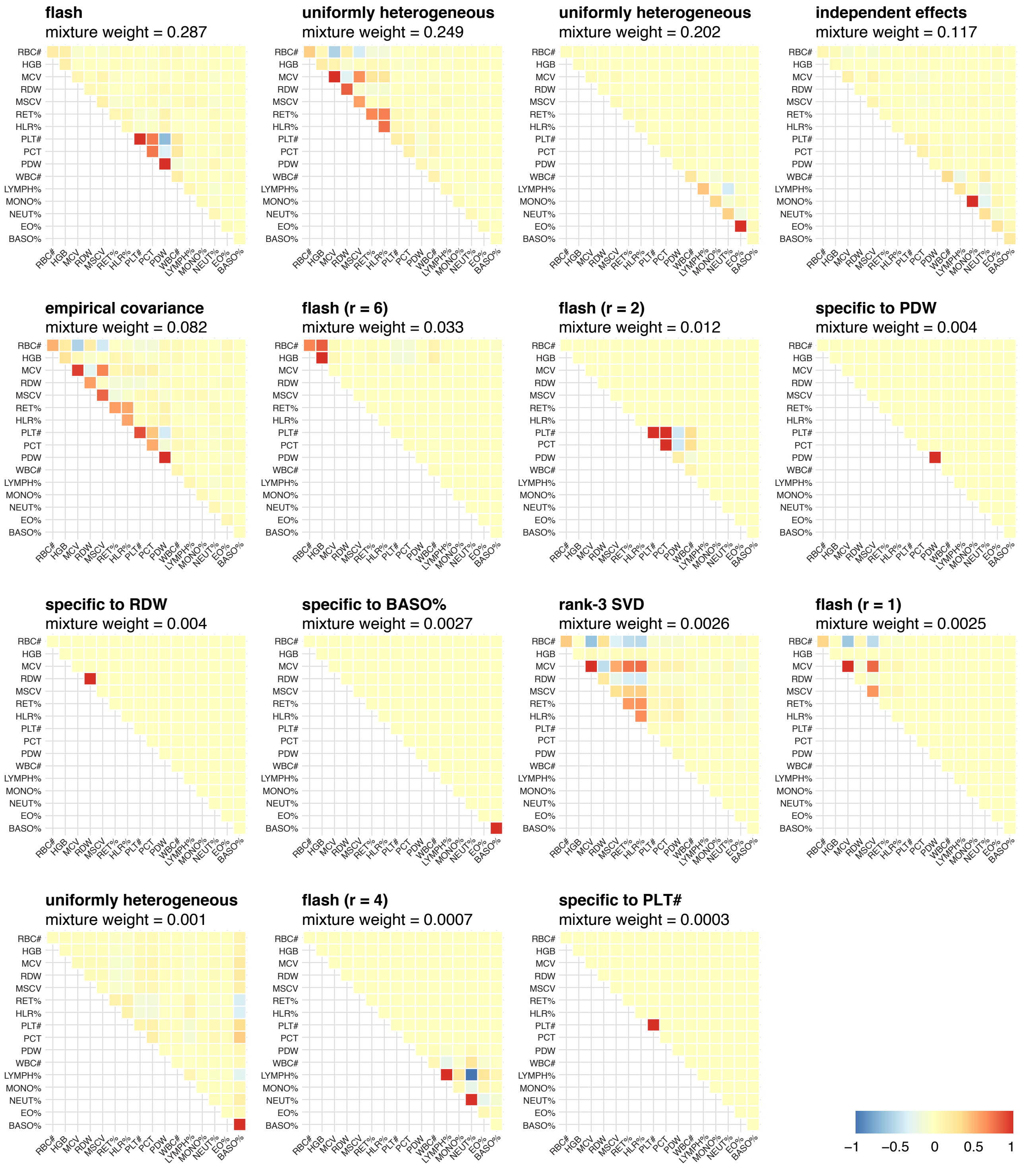

Supplementary Figure 13. Prior on multivariate SNP effects estimated from the UK Biobank blood cell traits. Each plot shows a 16 × 16 scaled covariance matrix $\boldsymbol{U}_k$ and its corresponding estimated mixture weight ω_k. These covariance matrices and mixture weights specify the mixture-of-multivariate normals prior used in the mvSuSiE analyses of the UK Biobank blood cell traits. For visualization purposes only, each plot shows the scaled covariance matrix $\boldsymbol{U}_k/s_k^2$, where s_k^2 is the absolute value of the largest (in magnitude) entry of $\boldsymbol{U}_k$, so that all of the plotted values lie between -1 and 1. Each covariance matrix is labeled by how the covariance estimate was initialized (see “Data-driven prior” in Online Methods).

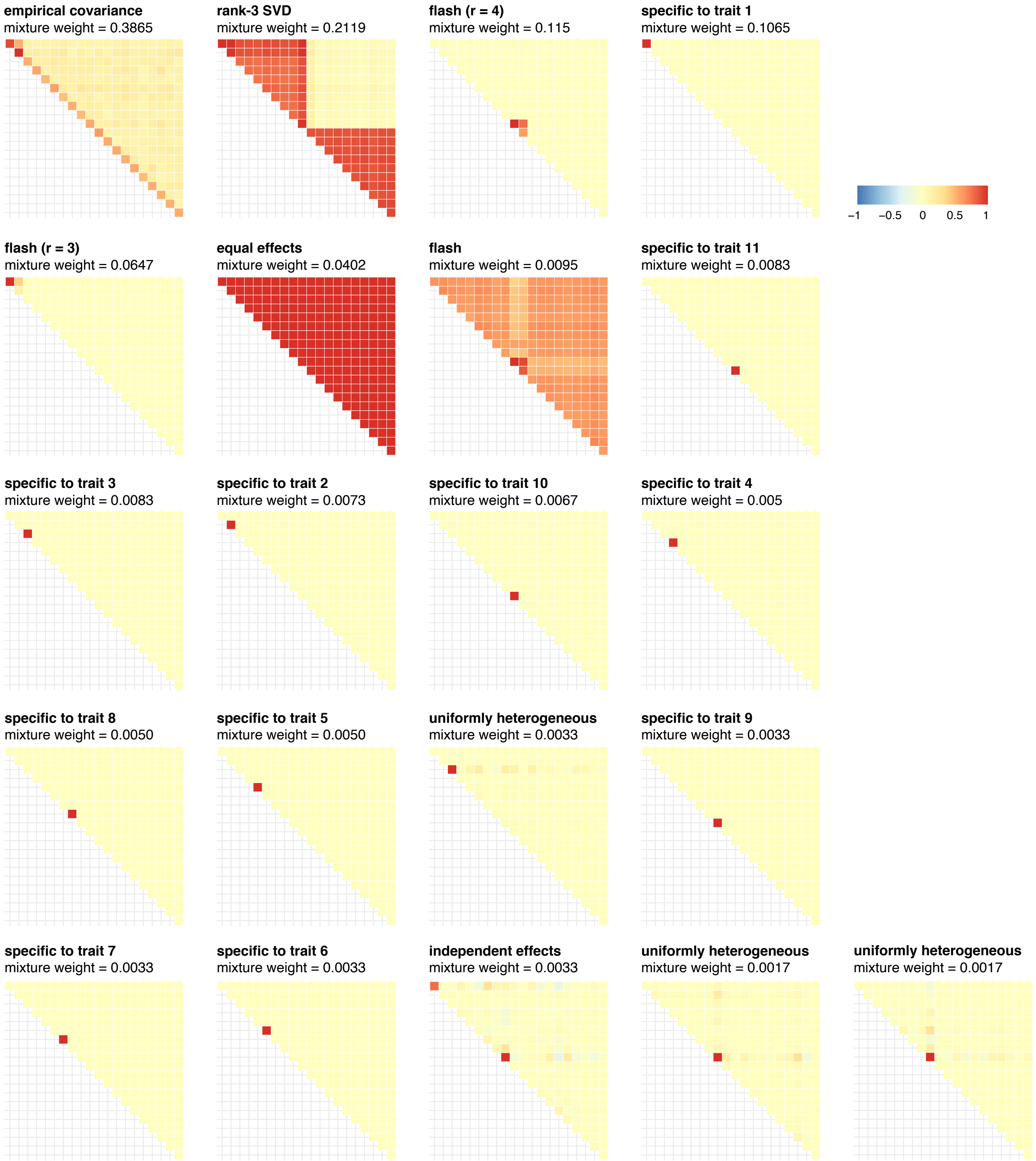

Supplementary Figure 14. Data-driven prior estimated in Scenario "a" simulations. Each plot shows a 20 × 20 scaled covariance matrix $\boldsymbol{U}_k$ and its corresponding estimated mixture weight ω_k. These covariance matrices and mixture weights describe the "data-driven" mixture-of-multivariate normals prior used in the mv-SuSiE analyses of the Scenario a simulated data sets. These covariance matrices capture many of the main effect sharing patterns used to simulate the data (compare to Supplementary Fig. 12) including tissue-specific effects (e.g., mixture components 8–18), independent effects (component 1), effects shared in subgroups (e.g., component 2), and effects shared equally across all tissues (component 6). For visualization purposes only, each plot shows the scaled covariance matrix $\boldsymbol{U}_k/s_k^2$, where s_k^2 is the absolute value of the largest (in magnitude) entry of $\boldsymbol{U}_k$, so that all of the plotted values lie between -1 and 1. Each covariance matrix is labeled by how the covariance estimate was initialized (see "Data-driven prior" in Online Methods).

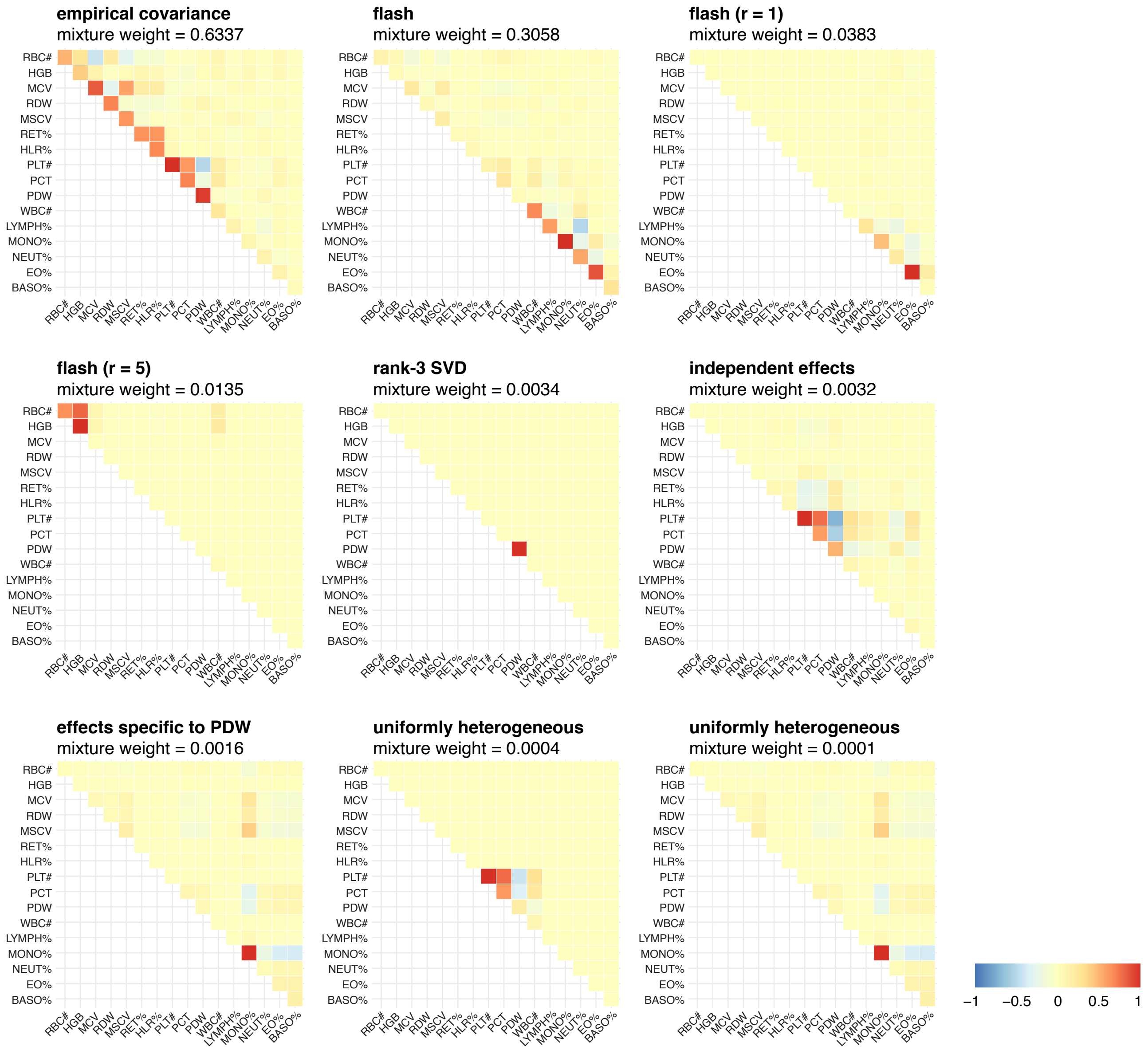

Supplementary Figure 15. Data-driven prior used in Scenario b. Each plot shows a 16 × 16 scaled covariance $\boldsymbol{U}_k$ and its corresponding estimated mixture weight ω_k. These covariance matrices and mixture weights describe the "data-driven" mixture-of-multivariate normals prior used in the mvSuSiE analyses of the Scenario b simulated data sets. Compare the covariances and mixture weights shown here to those in Supplementary Fig. 13, which were the covariances and weights used to simulate the data. For visualization purposes only, each plot shows the scaled covariance matrix $\boldsymbol{U}_k/s_k^2$, where s_k^2 is the absolute value of the largest (in magnitude) entry of $\boldsymbol{U}_k$, so that all of the plotted values lie between -1 and 1. Each covariance matrix is labeled by how the covariance estimate was initialized (see "Data-driven prior" in Online Methods).

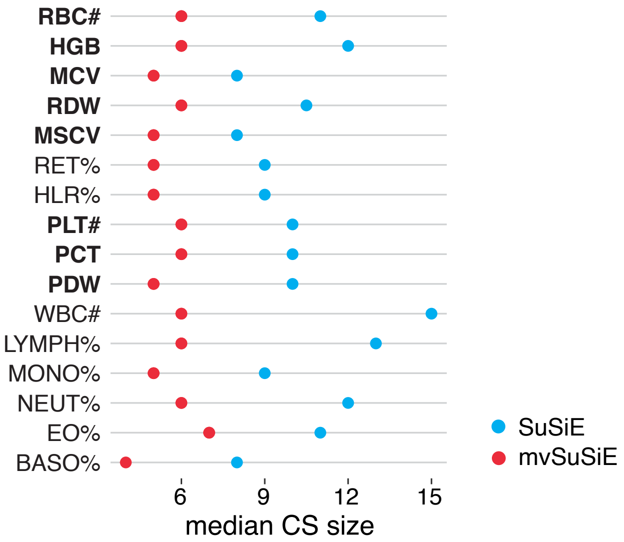

Supplementary Figure 16. Sizes of trait-wise CSs in SuSiE and mvSuSiE fine-mapping of UK Biobank blood cell traits. The plot compares the median sizes of the trait-wise CSs identified by SuSiE and mvSuSiE after removing CSs with purity less than 0.5.

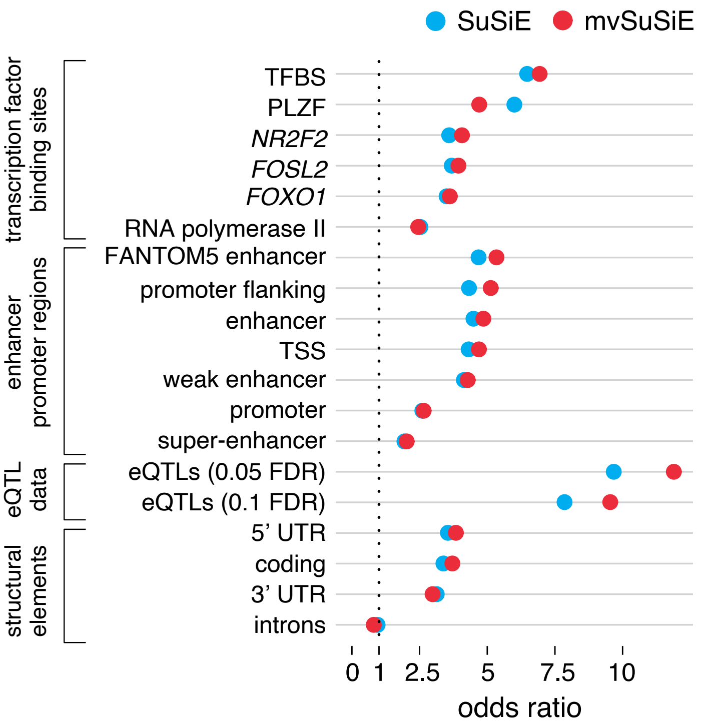

Supplementary Figure 17. Regulatory enrichment from SuSiE and mvSuSiE fine-mapping of blood cell traits. The plot shows enrichment odds ratios for SuSiE and mvSuSiE cross-trait fine-mapping results in non-cell-type-specific genomic regulatory annotations [12–16].

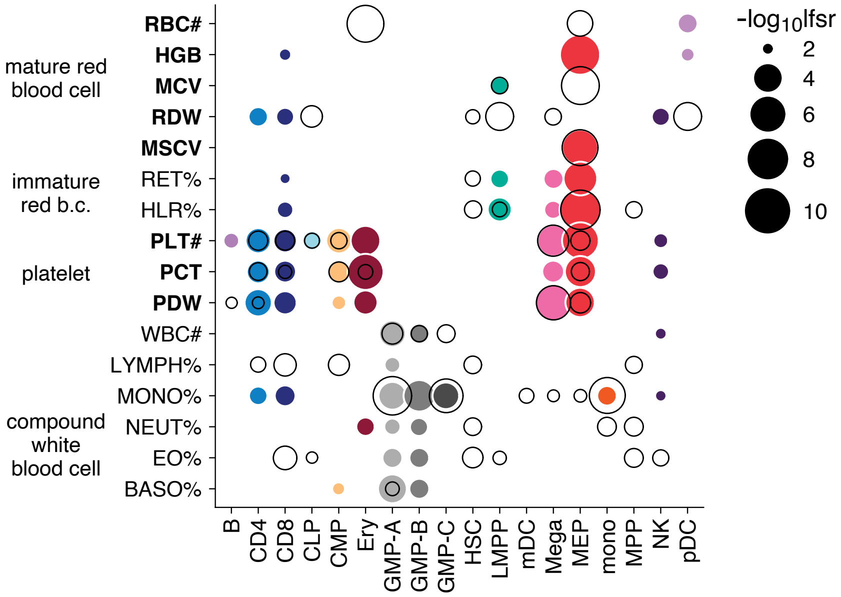

Supplementary Figure 18. Hematopoietic cell-type enrichment from SuSiE and mvSuSiE fine-mapping of blood cell traits. The show shows enrichment analysis results for accessible chromatin in hematopoietic cell populations [17]. SuSiE-based enrichments are shown as open circles, and mvSuSiE-based enrichments are colored according to the hematopoietic cell types, similar to [17]; only enrichments with *lfsr* < 0.01 are shown. TFBS = transcription factor binding site; PLZF = promyelocytic leukemia zinc finger protein; mono = monocyte; gran = granulocyte; ery = erythroid; mega = megakaryocyte; CD4 = CD4+ T cell; CD8 = CD8+ T cell; B = B cell; NK = natural killer cell; mDC = myeloid dendritic cell; pDC, = plasmacytoid dendritic cell; MPP = multipotent progenitor; LMPP = lymphoid-primed multipotent progenitor; CMP = common myeloid progenitor; CLP = common lymphoid progenitor; GMP = granulocyte-macrophage progenitor; MEP = megakaryocyte-erythroid progenitor.

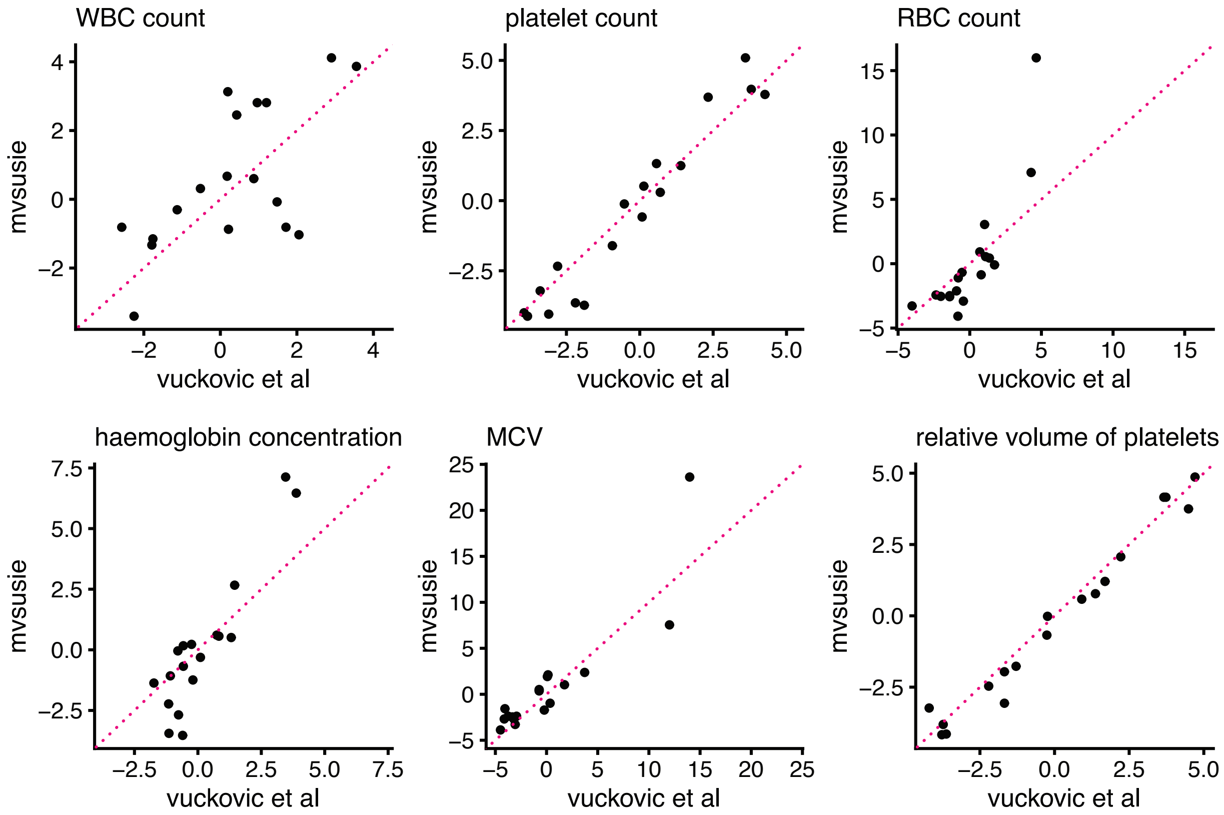

Supplementary Figure 19. Comparison of gchromVAR enrichments based on mvSuSiE vs. Vuckovic et al analysis. Here we compare the 6 blood cell traits that were included in both our mvSuSiE fine-mapping analyses and in the fine-mapping analyses of Vuckovic *et al.* [18]. Each plot shows the posterior *z*-scores (posterior means divided by posterior standard deviations) computed from the Vuckovic *et al* enrichment results against the posterior *z*-scores from our enrichment analysis. The Vuckovic *et al* *z*-scores were downloaded from `https://github.com/bloodcellgwas/manuscript_code`, then posterior *z*-scores were computed using adaptive shrinkage [19].
